# In Vitro and In Vivo Biological Evaluation of Indole-thiazolidine-2,4-dione Derivatives as Tyrosinase Inhibitors

**DOI:** 10.3390/molecules28227470

**Published:** 2023-11-07

**Authors:** Li Lu, Chunmei Hu, Xiaofeng Min, Zhong Liu, Xuetao Xu, Lishe Gan

**Affiliations:** 1 Guangdong Provincial Key Laboratory of Large Animal Models for Biomedicine, School of Biotechnology and Health Sciences, Wuyi University, Jiangmen 529020, China; wyuchemluli@126.com (L.L.); 15875066033@163.com (C.H.); m154650@163.com (X.M.); 2 College of Life Science and Technology, Jinan University, Guangzhou 510632, China; tliuzh@jnu.edu.cn

**Keywords:** tyrosinase inhibitor, indole, thiazolidine-2,4-dione, anti-melanogenic activity

## Abstract

Tyrosinase is an important rate-limiting enzyme in melanin biosynthesis. To find potential tyrosinase inhibitors with anti-melanogenic activity, a series of indole-thiazolidine-2,4-dione derivatives **5a**~**5z** were synthesized by incorporating indole with thiazolidine-2,4-dione into one compound and assayed for their biological activities. All compounds displayed tyrosinase inhibitory activities and **5w** had the highest anti-tyrosinase inhibitory activity with an IC_50_ value of 11.2 μM. Inhibition kinetics revealed **5w** as a mixed-type tyrosinase inhibitor. Fluorescence quenching results indicated that **5w** quenched tyrosinase fluorescence in a static process. CD spectra and 3D fluorescence spectra results suggested that the binding of **5w** with tyrosinase could change the conformation and microenvironment of tyrosinase. Molecular docking also represented the binding between **5w** and tyrosinase. Moreover, **5w** could inhibit tyrosinase activity and melanogenesis both in B16F10 cells and the zebrafish model. Therefore, compound **5w** could serve as a tyrosinase inhibitor with anti-melanogenic activity.

## 1. Introduction

Melanin, as a crucial biopolymer, is produced by melanocytes and is responsible for eye, skin, and hair color [1,2,3]. One important role of melanin is to protect the skin against ultraviolet damage [4,5,6]. Excessive ultraviolet exposure typically leads to abnormal melanin production and deposition, which generally results in several pigmentation skin diseases, including lentigo, freckles, and melisma [7,8,9]. 

Tyrosinase (EC 1.14.18.1) is one important rate-limiting enzyme for melanin production and is responsible for regulating the synthetic pathway of melanin production [10,11]. Tyrosinase is also a metalloenzyme, containing two bifunctional copper ions in its catalytic site [12,13]. In the process of melanin production, tyrosinase modulates the first two steps by the catalytic oxidation of L-tyrosine to dopaquinone [14]. In the first step, tyrosinase catalyzes L-tyrosine to convert it into 3,4-dihydroxyphenylalanine (L-DOPA), and in the second step, tyrosinase further oxidizes L-DOPA to dopaquinone, which is the important intermediate in melanin biosynthesis and is converted to melanin pigments by subsequent related chemical reactions [15,16,17]. Therefore, inhibiting tyrosinase activity is essential for the reduction in melanin synthesis [18]. Apart from their application in pigmentation disorders, tyrosinase inhibitors play important roles in the food industry. Tyrosinase can also catalyze the oxidation of phenolic molecules in fruit and vegetables into quinones, leading to enzymatic browning. Therefore, controlling tyrosinase activity is important for the quality of fruit and vegetables. Multitudinous natural and synthetic tyrosinase inhibitors displaying wide structural diversity have been identified [19,20,21]. But only a few tyrosinase inhibitors are used as efficacy agents in the pharmaceutical, cosmetic, and food industries, such as arbutin, paeonol, glabridin, and kojic acid. However, they still present some drawbacks like poor efficacy, skin irritation, and low stability. Therefore, it is urgent to develop safer and more effective tyrosinase inhibitors. 

**Figure 1:**
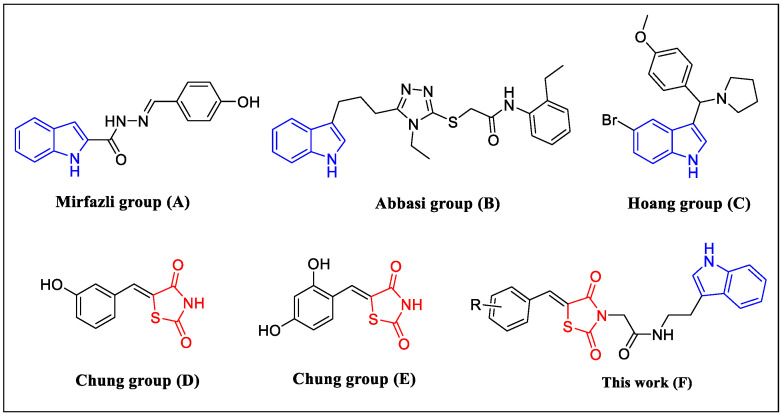
The reported indole or thiazolidine-2,4-dione based tyrosinase inhibitors, and the target indole-thiazolidine-2,4-dione derivatives in this work.

Indole scaffold with a structure of pyrrole in parallel with benzene has been one of the key structural nuclei in drug development due to its rich pharmacological activity and specific property of imitating peptide structure to bind with enzymes [22,23,24,25,26]. Previous studies have reported some indole derivatives as potential tyrosinase inhibitors. For example, the Mirfazli group introduced carbohydrazide into an indole scaffold to obtain substituted indole-carbohydrazides (Figure 1A), which presented excellent tyrosinase inhibitory activity [24]. The Abbasi group also synthesized the indole-N-ethyltriazole hybrids amalgamated with N-arylated ethanamides (Figure 1B) as tyrosinase inhibitors [25]. In another study, the Hoang group found 3-aminoalkylated indoles (Figure 1C) presented potential tyrosinase inhibitory activities [26]. Moreover, indole and its homologues widely exist in nature, showing good safety. The above investigations indicate that indole can serve as a core scaffold for developing potential tyrosinase inhibitors.

On the other hand, thiazolidine-2,4-diones (TZDs) are an important kind of antidiabetic agent and have been approved by the FDA to treat type 2 diabetes [27]. Their therapeutic target is peroxisome proliferator-activated receptor-γ (PPARγ). Recently, TZD derivatives have been reported to be potent inhibitors against tyrosinase, such as benzylidene-TZD derivatives (Figure 1D) [28] and 2,4-dihydroxybenzylidene-TZD (Figure 1E) [29]. The above analysis suggests that TZD can also serve as a core framework for designing potential tyrosinase inhibitors.

Drug design based on molecular hybridization involves combining two or more active molecules into a completely new molecule, which can inherit the advantageous structure of the parent molecule. This strategy can enhance the affinity or other performance of the molecule. Hence, to continue our efforts in the development of potential anti-tyrosinase agents [30,31,32], we report herein the synthesis of indole-thiazolidine-2,4-dione derivatives by incorporating indole with thiazolidine-2,4-dione to construct target compounds (Figure 1F). After determining their inhibitory activities against tyrosinase and the subsequent inhibitory mechanism, the effects on tyrosinase activity and melanogenesis in B16F10 cells and the zebrafish model were gradually evaluated.

## 2. Results and Discussion

### 2.1. Chemistry

**Scheme 1:**
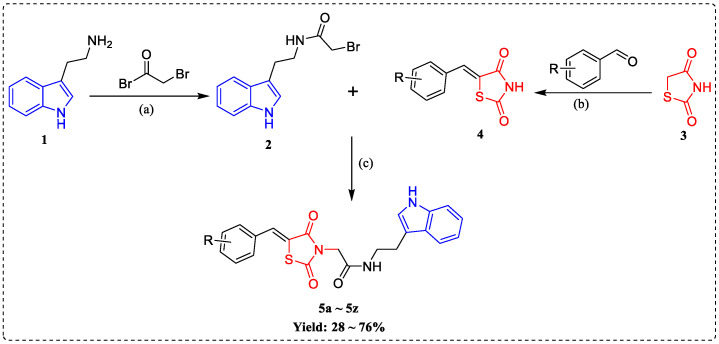
Synthetic route of compounds **5a**~**5z**. Reagents and conditions: (**a**) K_2_CO_3_, DCM, r.t., 1 h, yield 70%; (**b**) NaOH, EtOH, r.t., 6 h, yield 65%; (**c**) K_2_CO_3_, TBAB, DMF, r.t., 2 h, yield 28~76%.

Indole-thiazolidine-2,4-dione derivatives **5a**~**5z** were synthesized using a convergent approach according to Scheme 1. Tryptamine (**1**) underwent an amidation reaction with bromoacetyl bromide in the presence of potassium carbonate in DCM solution to produce intermediate (**2**). Thiazolidine-2,4-dione (**3**) reacted with substituted benzaldehyde in the presence of sodium hydroxide in EtOH solution to give intermediate (**4**). Then, compounds (**2**) and (**4**) underwent substitution reactions in the presence of tetrabutylammonium bromide and potassium carbonate in DMF solution to provide target products **5a**~**5z**. 

The detailed synthesis steps and NMR and HRMS data are shown in the Supplementary Materials. For example, the ^1^H NMR spectrum of compound **5a** exhibited a single peak at 10.85 ppm due to the imine proton of the indole ring; triple peaks at 8.43 ppm due to the imine proton of amide group; a single peak at 7.98 ppm attributed to vinyl protons; a single peak at 7.67 ppm; double peaks at 7.66 ppm (*J* = 1.1 Hz); double peaks at 7.58 ppm (*J* = 7.0 Hz); double peaks at 7.56 ppm (*J* = 5.9 Hz); double peaks at 7.53 ppm (*J* = 1.5 Hz); double peaks at 7.51 ppm (*J* = 7.5 Hz); multiple peaks at 7.36–7.31 ppm; a single peak at 7.16 ppm; multiple peaks at 7.09–7.04 ppm (*J* = 7.5 Hz); and multiple peaks at 7.01–6.96 ppm, all attributed to the aromatic protons of the benzene ring, respectively; and a single peak at 4.28 ppm; multiple peaks at 3.38–3.33 ppm; and triple peaks at 2.83 ppm (*J* = 7.4 Hz), all due to the methylene, respectively. The ^13^C NMR spectrum of **5a** showed two characteristic peaks at 167.63 and 165.83 ppm due to two carbonyl carbons of TZD; a characteristic peak at 165.34 ppm attributed to the carbonyl carbon of the amide group; a characteristic peak at 136.70 ppm due to the vinyl carbon; characteristic peaks at 133.77, 133.40, 131.25, 130.66, 129.92, 127.65, 123.24, 121.72, 121.41, 118.76, 118.69, and 111.86 ppm attributed to the aromatic carbon of the benzene ring; a characteristic peak at 112.01 ppm due to the carbon atom of TZD; characteristic peaks at 44.00 and 25.47 ppm attributed to methylene carbons. For the HRMS of compound **5a**, its molecular ion peak appeared at m/z 406.1224 as [M + H]^+^.

### 2.2. Tyrosinase Inhibitory Activity Assay

Based on the preliminary experimental results of the positive control, kojic acid, the anti-tyrosinase inhibitory activities of all compounds **5a**~**5z** were first screened at the initial concentration of 32 μM (Table 1). Among them, compounds **5l** and **5w** displayed the strongest tyrosinase inhibitory activities with inhibition ratios higher than 70%, while those of other compounds were unsatisfactory and below 50%. Their 50% inhibitory concentration (IC_50_) values were not further assayed, while compounds **5l** and **5w** were further detected for IC_50_ values of 13.3 and 11.2 μM, respectively, which were better than that of kojic acid (15.6 μM). That was to say that the hybridization strategy of indole with thiazolidine-2,4-dione into one compound would be an effective method for obtaining potential tyrosinase inhibitors.

The preliminary structure–activity relationship was analyzed based on the tyrosinase inhibitory activities of all compounds. It could be observed that substitution groups on the benzene ring resulted in differences in tyrosinase inhibitory activity. Compound **5a**, with no substituent group on the benzene ring, was selected as the parent molecule. The introduction of substituent groups (like methyl, fluorine, chlorine, bromine, hydroxyl, cyano, and nitro) would enhance the inhibitory activities, respectively, while the introduction of substituent groups, i.e., trifluoromethyl and methoxy groups would reduce the inhibitory activities. For the same substituent groups at different positions of the benzene ring, the meta-position contributes more to the inhibitory activity than the ortho- and para-position. In addition, for all substituent groups at the meta-position of the benzene ring, the contribution order of substituent groups on inhibitory activity is cyanide > bromine > chlorine > hydroxyl > methyl > fluorine > nitro > hydrogen > trifluoromethyl > methoxy. Cyanide groups contributed most to the tyrosinase inhibitory activity. These results will guide our further research works on the development of tyrosinase inhibitors.

### 2.3. Inhibition Kinetics 

**Figure 2:**
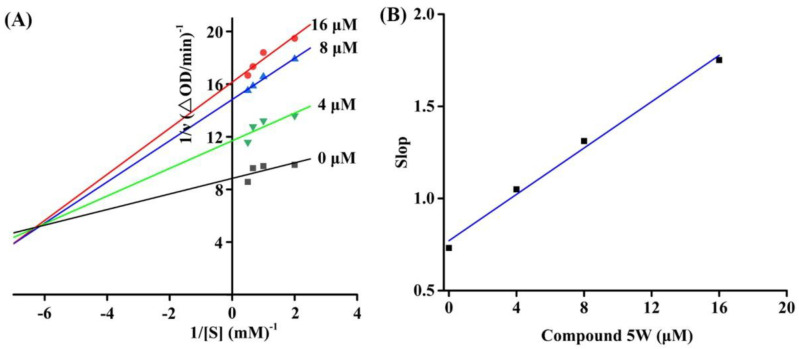
(**A**) The Lineweaver–Burk plots of **5w** against tyrosinase; (**B**) The plots of the slope versus **5w** concentration.

To understand the inhibition mechanism of this kind of compound on tyrosinase, the inhibition kinetics of the preferable compound **5w** were investigated. As displayed in Figure 2A, the Lineweaver–Burk plots of 1/v vs. 1/[S] produced a group of straight lines, which intersect in the second quadrant. With the increasing concentrations of **5w**, the Y-intercept of corresponding lines increased and the X-intercept decreased, meaning the decrease of V_max_ and K_m_. Compound **5w** could then be identified as a mixed-type inhibitor. This result also suggested that compound **5w** could bind with both free enzyme and enzyme–substrate complex. In addition, the inhibition constant (K_i_) of compound **5w** to tyrosinase was determined to be 11.73 μM from the plots of the slope vs. inhibitor concentration (Figure 2B). 

### 2.4. Fluorescence Quenching

**Figure 3:**
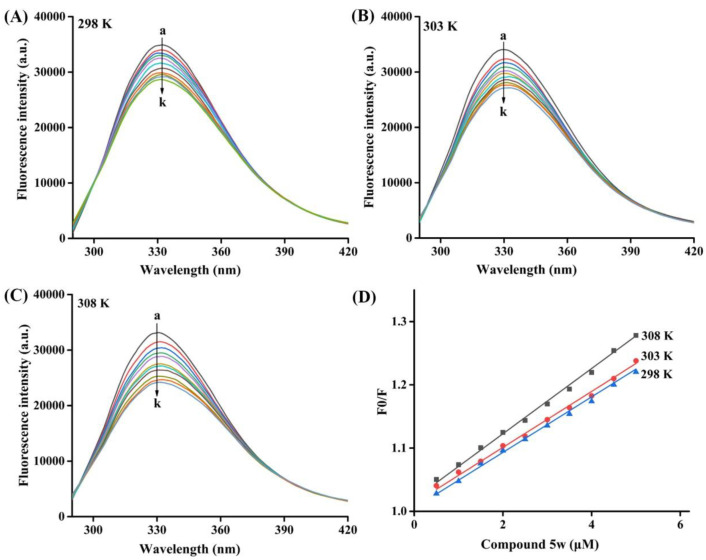
(**A**–**C**) The fluorescence quenching of **5w** against tyrosinase; curves a to k stood for **5w** concentrations of 0, 0.5, 1, 1.5, 2, 2.5, 3, 3.5, 4, 4.5, and 5 μM, respectively; (**D**) the Stern–Volmer plots of **5w**.

Fluorescence quenching, an effective method for monitoring the binding of a compound to its target protein, was operated to examine the binding of compound **5w** with tyrosinase. As presented in Figure 3A–C, tyrosinase demonstrated a fluorescence characteristic peak (332 nm) at 298, 303, and 308 K, respectively, due to the chromophore of tyrosinase. When compound **5w** was added by titration, the fluorescence spectra of tyrosinase gradually decreased. The results indicated that the binding between compound **5w** and tyrosinase occurred. Moreover, the fluorescence characteristic peak of tyrosinase slightly red-shifted with the titration addition of compound **5w**, which suggested there were microenvironmental changes in fluorescent chromophores.

For a further understanding of the binding process of compound **5w** with tyrosinase, the fluorescence quenching manner was assayed using the Stern–Volmer equation. In the Stern–Volmer plots of compound **5w** (Figure 3D), the lines at different temperatures presented a good linear, which illustrated only one quenching manner in their binding process. Although the quenching constant (*K*_sv_) values were slightly proportional to temperature, the quench rate constant (*K*_q_) values at different temperatures were all higher than 2 × 10^10^ L mol^−1^ S^−1^, which is the maximum scattering collision quenching constant (Table 2). The fluorescence quenching process of compound **5w** to tyrosinase was then a static process. In addition, the number of binding sites (n) was obtained to be about one, meaning there was only one binding site between compound **5w** and tyrosinase (Table 2). 

### 2.5. CD Spectra

**Figure 4:**
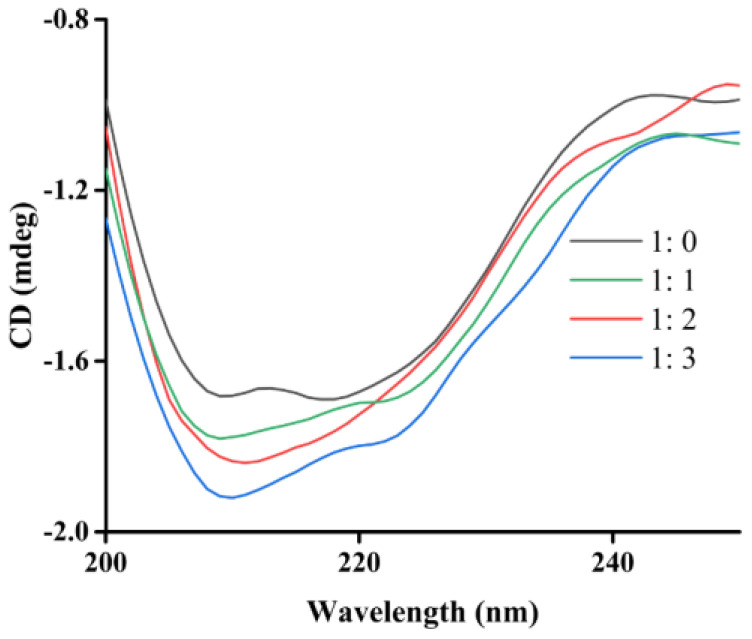
CD spectra of tyrosinase–compound **5w** complex.

CD spectra, one widely used method for determining the secondary structure of protein, were applied to assay the effects of **5w** on the conformation change of tyrosinase. The CD spectra of the tyrosinase–compound **5w** mixture were monitored and two negative characteristic bands around 210 and 222 nm appeared (Figure 4), which represented typical features of α-helixes. The addition of compound **5w** caused a significant increase in negative peak intensity and changes in characteristic bands, which suggested the conformation change of tyrosinase by binding with compound **5w**. The secondary structure contents of tyrosinase were then obtained from the CD spectra data and are listed in Table 3. The results indicated that treatment of compound **5w** could result in content changes in the secondary structure of tyrosinase. Compound **5w** especially (molar ratio of 3:1) could cause the content increase in the α-helix (from 8.7 to 9.0%); β-turn (from 19.8 to 20.1%); and random coil (from 30.6 to 35.5%); and the content decrease in the β-Sheet (from 34.0 to 32.7%), respectively.

### 2.6. Three-Dimensional Fluorescence Spectra 

**Figure 5:**
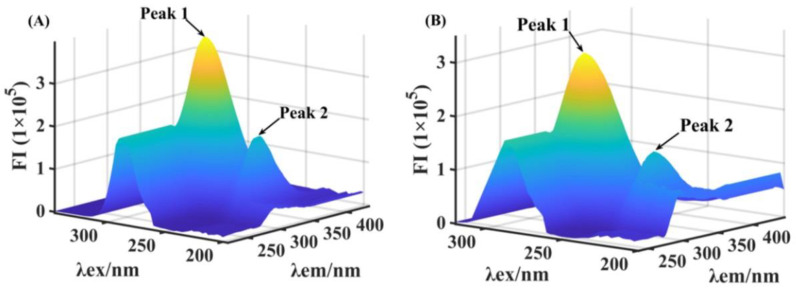
The 3D fluorescence spectra of tyrosinase (**A**) and tyrosinase–**5w** mixture (**B**).

Three-dimensional fluorescence spectra were employed to monitor the effects of compound **5w** on the conformation and microenvironment changes in tyrosinase. In the 3D fluorescence spectra of tyrosinase (Figure 5A), two characteristic peaks **1** and **2** appeared, which corresponded to Tyr and Trp residues and polypeptide strand transition, respectively. After being treated with compound **5w** (8.5 μM), the characteristic peaks **1** and **2** of tyrosinase reduced significantly accompanied by a slight shift, which indicated that the binding of **5w** to tyrosinase would result in conformation and microenvironment changes in tyrosinase. These results were inconsistent with the results of fluorescence quenching and CD spectra. 

### 2.7. Molecular Docking

**Figure 6:**
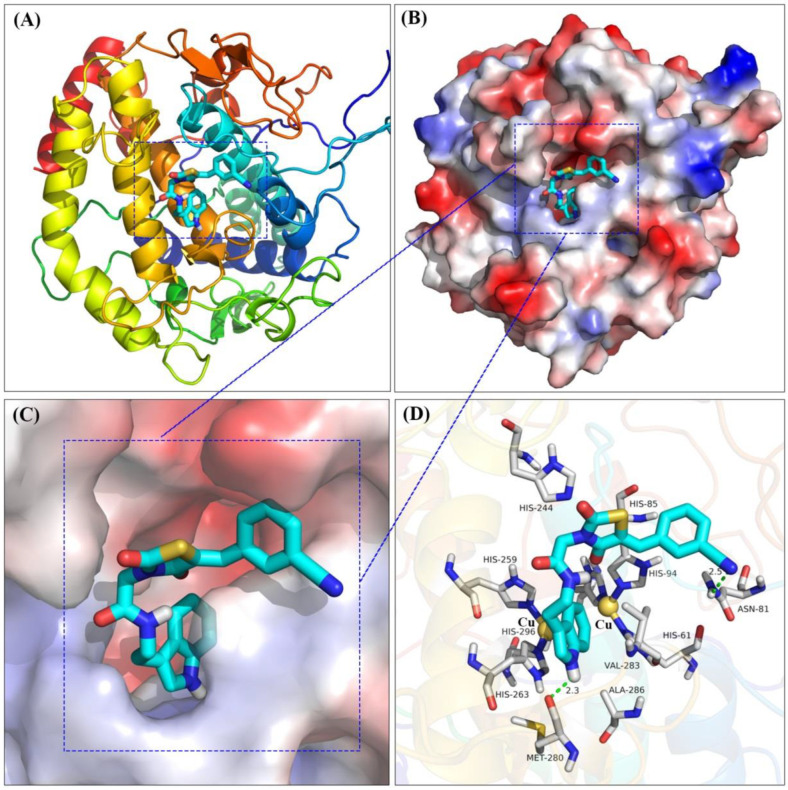
The molecular docking between compound **5w** and tyrosinase. (**A**–**C**) Binding pose of **5w** in the active site; (**D**) binding details of **5w**.

The binding information between compound **5w** and tyrosinase (PDB: 2Y9X) was investigated using SYBYL software (2.0). After compound **5w** and tyrosinase were prepared using the self-taking program, molecular docking was conducted, and the results are shown in Figure 6. From the docking results (Figure 6A–C), compound **5w** closely bound itself to the active site, especially with the indole ring section located in the copper ion catalytic region and the benzene ring section with a cyanide group located outside the active site. Then, the specific binding information was analyzed (Figure 6D) and it was found that the nitrogen hydrogen group in the indole ring formed one hydrogen bond with Met280 (2.3 Å) and the cyanide group in the benzene ring formed another hydrogen bond with Asn81 (2.5 Å). Moreover, compound 5w made hydrophobic interactions with His244, His263, Val283, and Ala286. The existence of the above interactions between compound **5w** and tyrosinase affected the structure of tyrosinase active sites, leading to a decrease in its enzyme activity.

### 2.8. Tyrosinase Activity and Melanogenesis Assay in B16F10 Cells

**Figure 7:**
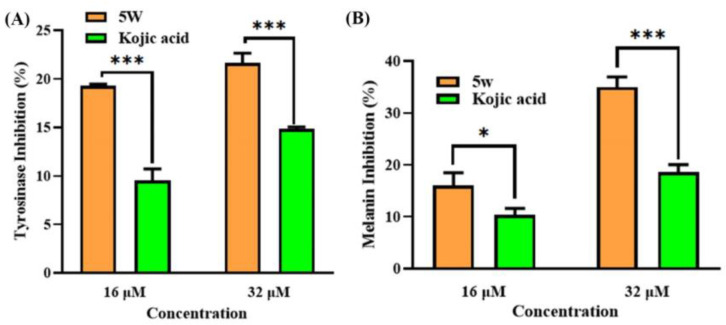
(**A**) The effects of **5w** on tyrosinase activity in B16F10 cells; (**B**) the effects of **5w** on melanogenesis in B16F10 cells. * *p* < 0.05, *** *p* < 0.001.

Although the synthesized compounds presented good activities against mushroom tyrosinase, due to the differences between mushroom tyrosinase and human tyrosinase [33], their effects on tyrosinase activity and melanogenesis both in B16F10 cells and zebrafish were assayed. The cytotoxicities of **5w** and kojic acid on B16F10 cells were assayed and the results showed that **5w** and kojic acid both had obvious effects on the cells’ viability up to 32 μM (Supplementary Materials). Therefore, the effects of **5w** and kojic acid on cellular tyrosinase activity and melanogenesis in B16F10 cells were detected at concentrations of 16 and 32 μM. As displayed in Figure 7A,B, **5w** and kojic acid could inhibit both cellular tyrosinase activity and melanogenesis in B16F10 cells, and **5w** presented obvious higher cellular anti-tyrosinase activity and anti-melanogenesis activity than kojic acid at the test concentrations. 

### 2.9. Tyrosinase Activity and Melanogenesis Assay in Zebrafish

**Figure 8:**
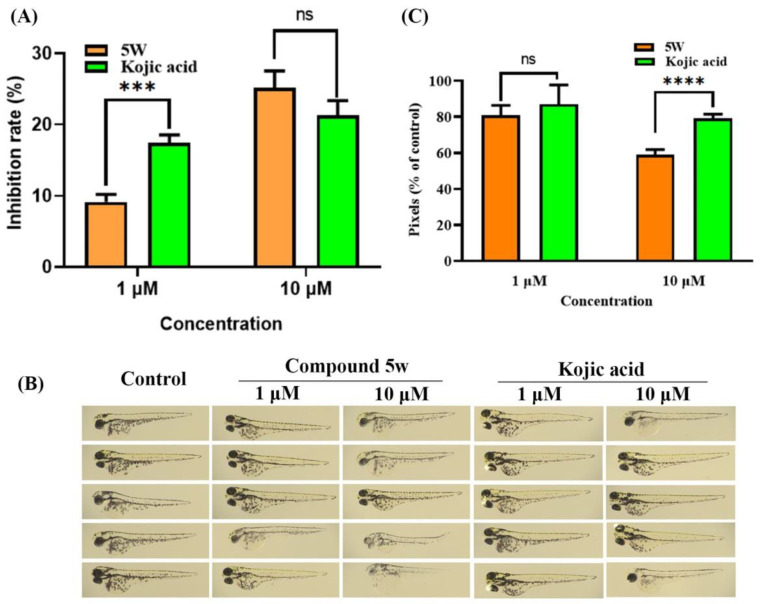
(**A**) The effects of **5w** on tyrosinase activity in zebrafish; (**B**) the embryo photos of each group; (**C**) the effects of **5w** on melanogenesis in zebrafish. “ns” means no significant difference (*p* > 0.05); *** *p* < 0.001, **** *p* < 0.0001.

Finally, the effects of **5w** on tyrosinase activity and the melanogenesis assay in zebrafish were investigated. The acute toxicity results showed that compound **5w** and kojic acid had an obvious toxicity to the zebrafish embryos up to 20 μM (Supplementary Materials). Then, the tyrosinase activity and melanogenesis in zebrafish treated with **5w** and kojic acid were assayed, and the results are displayed in Figure 8. It was observed that **5w** and kojic acid treatment could reduce the tyrosinase activity in zebrafish, and the compound **5w** had higher anti-tyrosinase activity than kojic acid at a concentration of 10 μM. From the embryo photos (Figure 8B), treatment with **5w** and kojic acid results in a pigmentation decrease in the embryo’s eyes, yolk sac, and notum compared to the control embryo. The grayscale values of whole embryos were determined using Image J software (1.8.0) (Figure 8C), and it was found that **5w** and kojic acid treatment also led to a decrease in grayscale values compared to the control embryo, and **5w** displayed a higher decrease. All the above results show that **5w** could inhibit the tyrosinase activity in vitro and in vivo, therefore reducing melanogenesis both in B16F10 cells and zebrafish.

## 3. Materials and Methods

### 3.1. Chemistry

Mushroom tyrosinase and L-DOPA were supplied by Sigma-Aldrich (Shanghai, China). All other reagents were commercially available. NMR spectra were recorded on a Bruker (500 MHz) NMR spectrometer. HRMS data were measured on Apex II by means of the ESI technique. 

### 3.2. Tyrosinase Inhibition and Kinetics Study

The tyrosinase inhibitory activities of compounds **5a**~**5z** were determined according to previous reports [34,35]. In brief, 10 μL of compound was added into 140 μL of tyrosinase, incubated for 5 min at room temperature. Then, 50 μL of L-DOPA was added, followed by the measurement of absorbance change at 475 nm. The inhibition rate at different concentrations was calculated and compared to the blank control. 

The kinetics studies of **5w** were conducted using the above experimental procedure. The absorbance changes in the mixture containing **5w** (0, 4, 8, and 16 μM), tyrosinase (66.7 U/mL), and L-DOPA (2, 4, 6, and 8 mM) were recorded, respectively. The data were analyzed using Lineweaver–Burk plots [36].

### 3.3. Fluorescence Quenching

According to a previous report [37], the fluorescence quenching titration was run. In total, 1 μL of **5w** solution was progressively titrated into 2 mL of tyrosinase (0.17 mg/mL), followed by fluorescence spectra determination (λex = 280 nm) at temperatures of 298, 303, and 308 K, respectively. The quenching parameters were obtained using the Stern–Volmer equation.

### 3.4. CD Spectra

The CD spectra of tyrosinase (33 μM) and tyrosinase–compound **5w** mixture were measured at room temperature, respectively, based on previous reports [38]. The test wavelengths were 195~250 nm. 

### 3.5. Three-Dimensional Fluorescence Spectra 

Based on a previous report [39], the 3D fluorescence spectra of tyrosinase (0.17 mg/mL) and tyrosinase–compound **5w** mixture were measured, respectively, at room temperature. The test wavelengths were 200–400 nm.

### 3.6. Molecular Docking

The molecular docking of compound **5w** with tyrosinase was conducted using SYBYL software based on previous reports [40,41]. Compound **5w** was treated with energy minimization accompanied by charge addition with the Gasteiger–Huckel model. The crystal structure of Agaricus bisporus tyrosinase (PDBID: 2Y9X) was obtained from the RCSB. Tyrosinase protein was then treated by removing the ligand and water, adding hydrogen atoms and charge, and repairing end residues, followed by the generation of the active pocket. Finally, the docking of compound **5w** with tyrosinase was run.

### 3.7. Cell Assay

#### 3.7.1. Cell Cytotoxicity

The effects of compound **5w** and kojic acid on the cells’ viability were determined using the MTT method. B16F10 cells (5000 cells/well) were seeded into the 96-well plate for 24 h, followed by the treatment of compound **5w** or kojic acid (4~32 μM) for another 48 h. Then, MTT solution (0.5 mg/mL) was added and incubated for 4 h. DMSO (100 μL) was used to dissolve the violet precipitates, and then the mixture was measured for its absorbance at 570 nm. 

#### 3.7.2. Tyrosinase Activity in B16F10 Cells

The tyrosinase inhibitory activity of **5w** in B16F10 cells was determined. B16F10 cells (1 × 10^5^ cells/well) were seeded into the 6-well plate for 24 h and subsequently treated with compound **5w** or kojic acid for another 48 h. Then, the harvested cells were suspended into the appropriate amount of Triton X-100 (1%) and frozen for 1 h at −80 °C. After it had thawed, the mixture was centrifuged to collect the supernatant. Then, its protein concentration was determined using a BCA Kit. After 20 μL of L-DOPA (10 mM) was added into 80 μL of protein solution, the absorbance change at 475 nm was measured. 

#### 3.7.3. Melanin Content in B16F10 Cells

B16F10 cell treatment was consistent with the above method. The harvested cells were suspended into NaOH (1 M, 200 μL). After boiling for 1 h, the mixture was measured to determine the absorbance change at wavelength of 405 nm. 

### 3.8. Zebrafish Assay

#### 3.8.1. Acute Toxicity

In this experiment, 6~8 hpf embryos (1 embryo per well) were seeded in 24-well plates, then the compound **5w** or kojic acid (0~40 μM) was added, followed by observation of the survival rate at 72 hpf. 

#### 3.8.2. Tyrosinase Activity in Zebrafish

The embryos treated with compound **5w** or kojic acid were lysed in PBS, followed by centrifugation to obtain the supernatant. The appropriate amount of supernatant, equal L-DOPA (5 mg/mL), was added and the absorbance at 475 nm was determined. 

#### 3.8.3. Melanin Content in Zebrafish

The embryos treated with compound **5w** or kojic acid were observed under a microscope and the melanin content was assayed using Image J software.

## 4. Conclusions

In summary, a series of indole-thiazolidine-2,4-dione derivatives **5a**~**5z** were synthesized by incorporating indole with thiazolidine-2,4-dione into the target compounds, and their potential anti-tyrosinase and anti-melanogenic activities were evaluated. All compounds displayed tyrosinase inhibitory activities, and **5w** had the highest anti-tyrosinase inhibitory activity with an IC_50_ value of 11.2 μM compared to the positive control kojic acid (IC_50_ = 15.6 μM). Inhibition kinetics indicated **5w** as a mixed-type inhibitor of tyrosinase. Fluorescence quenching results illustrated that the binding of **5w** to tyrosinase could quench the fluorescence of tyrosinase with a static quenching process. CD spectra and 3D fluorescence spectra suggested that the binding of **5w** to tyrosinase could change the conformation and microenvironment of tyrosinase. Molecular docking revealed the detailed binding between **5w** and tyrosinase. Moreover, compound **5w** could inhibit tyrosinase activity and melanogenesis both in B16F10 cells and the zebrafish model. Therefore, compound **5w** could serve as a tyrosinase inhibitor with anti-melanogenic activity.

## Figures and Tables

**Table 1:** Tyrosinase inhibitory activities of compounds **5a**~**5z**.

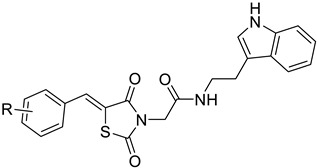							
No.	R	Inhibition (%)	IC_50_ (μM)	No.	R	Inhibition (%)	IC_50_ (μM)
**5a**	H	14.2	-	**5n**	2-CF_3_	3.8	-
**5b**	2-Me	19.3	-	**5o**	3-CF_3_	4.5	-
**5c**	3-Me	33.2	-	**5p**	4-CF_3_	3.0	-
**5d**	4-Me	15.1	-	**5q**	2-OMe	1	-
**5e**	2-F	16.5	-	**5r**	3-OMe	2	-
**5f**	3-F	32.6	-	**5s**	4-OMe	1	-
**5g**	4-F	27.4	-	**5t**	2-OH	26.9	-
**5h**	2-Cl	16.8	-	**5u**	3-OH	36.2	-
**5i**	3-Cl	37.6	-	**5v**	4-OH	24.6	-
**5j**	4-Cl	36.6	-	**5w**	3-CN	71.5	11.2
**5k**	2-Br	31.7	-	**5x**	4-CN	24.6	-
**5l**	3-Br	70.3	13.3	**5y**	3-NO_2_	19.8	-
**5m**	4-Br	19.5	-	**5z**	4-NO_2_	19.1	-
Kojic acid			15.6				

“-” means not determined.

**Table 2:** Quenching parameters between compound **5w** and tyrosinase.

T	*K*_SV_ (×10^4^ Lmol^−1^)	*K*_q_ (×10^12^ Lmol^−1^)	n
298	3.77	3.77	0.72
303	3.84	3.84	0.74
308	4.47	4.47	0.82

**Table 3:** The secondary structure content of tyrosinase.

[Enzyme]: [5w]	α-Helix (%)	β-Sheet (%)	β-Turn (%)	Random Coil (%)
1: 0	8.7	34.0	19.8	30.6
1: 1	8.8	33.6	19.9	32.5
1: 2	8.9	33.1	20.0	34.5
1: 3	9.0	32.7	20.1	35.5

## Data Availability

The data are contained within the article.

## Supplementary Materials

The following supporting information can be downloaded at: https://www.mdpi.com/article/10.3390/molecules28227470/s1, Synthesis of indole-thiazolidine-2,4-dione derivatives **5a**~**5z**; ^1^H NMR and ^13^C NMR of compounds **5a**~**5z**; HRMS of compounds **5a**~**5z**; Cytotoxicity of 5w on B16F10 cells and zebrafish model [42,43,44].
