# Supplementary material for: In Vitro and In Vivo Biological Evaluation of Indole-thiazolidine-2,4-dione Derivatives as Tyrosinase Inhibitors
Source: Molecules. 2023 Nov 7;28(22):7470. doi: 10.3390/molecules28227470 (PMC10673563; doi:10.3390/molecules28227470)
Supplement: Supplementary file 1 [file molecules-28-07470-s001.zip › molecules-2691757-supplementary.pdf]

Supplementary material

In Vitro and In Vivo Biological Evaluation of Indole-thiazolidine-2,4-dione Derivatives as Tyrosinase Inhibitors

Li Lu [1,†], Chunmei Hu [1,†], Xiaofeng Min [1], Zhong Liu [2], Xuetao Xu [1,*] and Lishe Gan [1,*]

[1] Guangdong Provincial Key Laboratory of Large Animal Models for Biomedicine, School of Biotechnology and Health Sciences, Wuyi University, Jiangmen 529020, China; wyuchemluli@126.com (L.L.); 15875066033@163.com (C.H.); m154650@163.com (X.M.)
[2] College of Life Science and Technology, Jinan University, Guangzhou 510632, China; tliuzh@jnu.edu.cn
* Correspondence: wyuchemxxt@126.com (X.X.); ganlishe@163.com (L.G.)
† These authors contributed equally to this work.

*1. Synthesis of indole-thiazolidine-2,4-dione derivatives **5a~5z**…………..2-10*

*2. ^{1}H NMR and ^{13}C NMR of compounds **5a~5z**……………………………….11-36*

*3. HRMS of compounds **5a~5z**…………………………………………….………….37-49*

4. Cytotoxicity of 5w on B16F10 cells and zebrafish model……………….50

5. References……………………………………………………………………….51

1. Synthesis of indole-thiazolidine-2,4-dione derivatives ***5a~5z***

Tryptamine **1** (1 mmol, 160.2 mg, purity: 99%), bromoacetyl bromide (2 mmol, 403.6 mg, purity: 98%) and potassium carbonate (3 mmol, 414.6 mg, purity: 99%) were added into DCM (5 mL) in steps and stirred at room temperature until the reaction completed. After the reaction was quenched with water, the mixture was extracted with DCM, followed by the dry to obtain intermediate **2** [1].

Thiazolidine-2,4-dione **3** (1 mmol, 117.1 mg, purity: 97%), substituted benzaldehyde (1 mmol, purity: 99%) and sodium hydroxide (2 mmol, 80.0 mg, purity: 95%) were added into EtOH (35 mL) in steps and stirred at room temperature until the reaction completed. Then the mixture was filtered and the crude product was washed with hexane to produce intermediate **4** [2,3].

Compound **2** (0.5 mmol), compound **4** (0.5 mmol), tetrabutylammonium bromide (0.05 mmol, 16.1 mg, purity: 99%), and potassium carbonate (1.5 mmol, 207.3 mg, purity: 99%) were added into DMF (5 mL) in steps and stirred at room temperature. After the reaction was completed, the mixture was adjusted the pH to 7 and filtered, followed by the recrystallization of crude product using DMF to yield target compounds **5a** ~ **5z.**

(**5a**, $C_{22}H_{19}N_3O_3S$). Yellow solid; Yield 67%; m.p. 202.3 - 205.3 °C.^{1}H NMR (500 MHz, DMSO-*d6*) δ 10.85 (s, 1H), 8.43 (t, *J* = 5.7 Hz, 1H), 7.98 (s, 1H), 7.67 (s, 1H), 7.66 (d, *J* = 1.1 Hz, 1H), 7.58 (d, *J* = 7.0 Hz, 1H), 7.56 (d, *J* = 5.9 Hz, 1H), 7.53 (d, *J* = 1.5 Hz, 1H), 7.51 (d, *J* = 7.5 Hz, 1H), 7.36 - 7.31 (m, 1H), 7.16 (s, 1H), 7.09 - 7.04 (m, *J* = 7.5 Hz, 1H), 7.01 - 6.96 (m, 1H), 4.28 (s, 2H), 3.38 - 3.33 (m, 2H), 2.83 (t, *J* = 7.4 Hz, 2H).^{13}C NMR (126 MHz, DMSO-*d6*) δ 167.63, 165.83, 165.34, 136.70, 133.77, 133.40, 131.25, 130.66, 129.92, 127.65, 123.24, 121.72, 121.41, 118.76, 118.69, 112.01, 111.86, 44.00, 25.47. HRMS (ESI-MS) m/z: $[M+H]^+$ calcd for $C_{22}H_{19}N_3O_3S$: 406.1224; found: 406.1224.

(**5b**, $C_{23}H_{21}N_3O_3S$). White solid; Yield 73%; m.p. 223.8 - 226.3 °C. ^{1}H NMR (500 MHz, DMSO-*d6*) δ 10.86 (s, 1H), 8.44 (t, *J* = 5.7 Hz, 1H), 8.07 (s, 1H), 7.54 (d, *J* = 7.8 Hz, 1H), 7.48 (d, *J* = 7.6 Hz, 1H), 7.41 (d, *J* = 7.5 Hz, 1H), 7.39 (s, 1H), 7.37 (d, *J* = 3.6 Hz, 1H), 7.35 (d, *J* = 8.1 Hz, 1H), 7.17 (s, 1H), 7.10 - 7.05 (m, 1H), 7.01 -

6.97 (m, 1H), 4.28 (s, 2H), 3.40 - 3.35 (m, 2H), 2.84 (t, *J* = 7.4 Hz, 2H), 2.43 (s, 3H). ^{13}C NMR (126 MHz, DMSO-*d6*) δ 167.87, 165.57, 165.35, 139.32, 136.70, 132.54, 131.68, 131.54, 131.08, 127.70, 127.66, 127.23, 123.44, 123.25, 121.41, 118.76, 118.69, 112.01, 111.86, 43.93, 25.47, 19.88. HRMS (ESI-MS) m/z: $[M+H]^+$ calcd for $C_{23}H_{21}N_3O_3S$: 420.1381; found: 420.1379.

(**5c**, *$C_{23}H_{21}N_3O_3S$*). Yellow solid; Yield 76%; m.p. 229.3 - 230.1 °C. ^{1}H NMR (500 MHz, DMSO-*d6*) δ 10.85 (s, 1H), 8.43 (t, *J* = 5.7 Hz, 1H), 7.93 (s, 1H), 7.53 (d, *J* = 7.9 Hz, 1H), 7.46 (s, 1H), 7.45 (s, 1H), 7.34 (d, *J* = 3.1 Hz, 1H), 7.33 (s, 1H), 7.16 (s, 1H), 7.07 (t, *J* = 7.5 Hz, 1H), 6.98 (t, *J* = 7.4 Hz, 1H), 4.27 (s, 2H), 3.37 - 3.33 (m, 2H), 2.83 (t, *J* = 7.4 Hz, 2H), 2.38 (s, 3H). ^{13}C NMR (126 MHz, DMSO-*d6*) δ 167.66, 165.84, 165.36, 139.29, 136.70, 133.86, 133.38, 132.01, 131.26, 129.83, 127.68, 127.65, 123.25, 121.50, 121.41, 118.77, 118.69, 112.01, 111.86, 43.98, 25.48, 21.41. HRMS (ESI-MS) m/z: $[M+H]^+$ calcd for $C_{23}H_{21}N_3O_3S$: 420.1381; found: 420.1381.

(**5d**, *$C_{23}H_{21}N_3O_3S$*). Yellow solid; Yield 69%; m.p. 279.3 - 280.1 °C. ^{1}H NMR (500 MHz, DMSO-*d6*) δ 10.86 (s, 1H), 8.42 (t, *J* = 5.7 Hz, 1H), 7.94 (s, 1H), 7.56 (s, 1H), 7.55 (s, 1H), 7.53 (d, *J* = 7.8 Hz, 1H), 7.39 (s, 1H), 7.37 (s, 1H), 7.34 (d, *J* = 8.1 Hz, 1H), 7.16 (s, 1H), 7.09 - 7.04 (m, 1H), 6.98 (t, *J* = 7.4 Hz, 1H), 4.27 (s, 2H), 3.37 - 3.33 (m, 2H), 2.83 (t, *J* = 7.4 Hz, 2H), 2.37 (s, 3H).^{13}C NMR (126 MHz, DMSO-*d6*) δ 167.66, 165.90, 165.37, 141.62, 136.70, 133.84, 130.74, 130.66, 130.55, 127.65, 123.24, 121.41, 120.46, 118.76, 118.69, 112.01, 111.86, 43.96, 25.47, 21.61. HRMS (ESI-MS) m/z: $[M+H]_+$ calcd for $C_{23}H_{21}N_3O_3S$: 420.1381; found: 420.1379.

(**5e**, *$C_{22}H_{18}FN_3O_3S$*). Yellow solid; Yield 63%; m.p. 187.4 - 188.1 °C. ^{1}H NMR (500 MHz, DMSO-*d6*) δ 10.85 (s, 1H), 8.43 (s, 1H), 7.95 (s, 1H), 7.63 (d, *J* = 5.5 Hz, 1H), 7.60 (d, *J* = 8.1 Hz, 1H), 7.53 (d, *J* = 7.8 Hz, 1H), 7.42 (t, *J* = 8.5 Hz, 2H), 7.34 (d, *J* = 8.2 Hz, 1H), 7.16 (s, 1H), 7.07 (t, *J* = 7.6 Hz, 1H), 6.98 (t, *J* = 7.4 Hz, 1H), 4.28 (s, 2H), 3.40 - 3.35 (m, 2H), 2.84 (t, *J* = 7.5 Hz, 2H). ^{13}C NMR (126 MHz, DMSO-*d6*) δ 167.32, 165.54, 165.25, 162.01, 160.00, 136.70, 133.61, 133.54, 129.52, 127.65, 126.01, 125.98, 124.92, 124.87, 124.52, 123.23, 123.19, 121.40, 121.32, 121.22, 118.75, 118.71, 118.68, 116.90, 116.73, 112.00, 111.85, 55.39, 44.09, 25.46. HRMS (ESI-MS) m/z: $[M+H]^+$ calcd for $C_{22}H_{18}FN_3O_3S$: 424.1130; found: 424.1130.

(**5f**, *C22H18FN3O3S*). Yellow solid; Yield 69%; m.p. 321.6 - 322.7 °C. ^{1}H NMR (500 MHz, DMSO-*d6*) δ 10.86 (s, 1H), 8.44 (t, *J* = 5.7 Hz, 1H), 7.99 (s, 1H), 7.65 - 7.58 (m, 1H), 7.54 (d, *J* = 3.0 Hz, 1H), 7.52 (d, *J* = 5.0 Hz, 1H), 7.49 (d, *J* = 7.7 Hz, 1H), 7.37 (t, *J* = 8.6 Hz, 1H), 7.34 (d, *J* = 8.2 Hz, 1H), 7.16 (d, *J* = 2.6 Hz, 1H), 7.07 (t, *J* = 7.5 Hz, 1H), 6.99 (t, *J* = 7.4 Hz, 1H), 4.28 (s, 2H), 3.38 - 3.34 (m, 2H), 2.84 (t, *J* = 7.4 Hz, 2H). ^{13}C NMR (126 MHz, DMSO-*d6*) δ 167.36, 165.68, 165.29, 163.75, 161.81, 136.70, 135.78, 135.71, 132.38, 132.03, 131.96, 127.65, 126.13, 123.39, 123.24, 121.42, 118.77, 118.69, 118.10, 117.93, 117.55, 117.37, 112.01, 111.86, 44.06, 25.47. HRMS (ESI-MS) m/z: $[M+H]^+$ calcd for $C_{22}H_{18}FN_3O_3S$: 424.1130; found: 424.1130.

(**5g**, *$C_{22}H_{18}FN_3O_3S$*). White solid; Yield 74%; m.p. 222.4 - 223.7 °C. ^{1}H NMR (500 MHz, DMSO-*d6*) δ 10.85 (s, 1H), 8.43 (t, *J* = 5.7 Hz, 1H), 8.00 (s, 1H), 7.75 (d, *J* = 5.5 Hz, 1H), 7.73 (s, 1H), 7.53 (d, *J* = 7.8 Hz, 1H), 7.43 (d, *J* = 8.8 Hz, 1H), 7.40 (s, 1H), 7.34 (d, *J* = 8.1 Hz, 1H), 7.16 (s, 1H), 7.06 (t, *J* = 6.9 Hz, 1H), 6.98 (t, *J* = 7.4 Hz, 1H), 4.27 (s, 2H), 3.37 - 3.33 (m, 2H), 2.83 (t, *J* = 7.4 Hz, 2H). ^{13}C NMR (126 MHz, DMSO-*d6*) δ 167.38, 165.73, 165.30, 136.70, 135.84, 132.47, 132.30, 132.08, 130.00, 129.86, 127.65, 123.24, 122.47, 121.41, 118.76, 118.69, 112.01, 111.86, 44.05, 25.47. HRMS (ESI-MS) m/z: $[M+H]^+$ calcd for $C_{22}H_{18}FN_3O_3S$: 424.1130; found: 424.1130.

(**5h**, *$C_{22}H_{18}ClN_3O_3S$*). Yellow solid; Yield 34%; m.p. 189.8 - 190.4 °C. ^{1}H NMR (500 MHz, DMSO-*d6*) δ 10.86 (s, 1H), 8.45 (t, *J* = 5.7 Hz, 1H), 8.07 (s, 1H), 7.69 - 7.65 (m, 1H), 7.65 - 7.62 (m, 1H), 7.59 - 7.56 (m, 1H), 7.54 (d, *J* = 4.3 Hz, 1H), 7.52 (d, *J* = 4.9 Hz, 1H), 7.34 (d, *J* = 8.1 Hz, 1H), 7.16 (s, 1H), 7.07 (t, *J* = 7.5 Hz, 1H), 6.98 (t, *J* = 7.4 Hz, 1H), 4.28 (s, 2H), 3.38 - 3.34 (m, 2H), 2.83 (t, *J* = 7.4 Hz, 2H). ^{13}C NMR (126 MHz, DMSO-*d6*) δ 167.38, 165.45, 165.26, 136.71, 135.00, 132.65, 132.31, 131.39, 130.91, 130.83, 129.49, 129.36, 128.86, 128.70, 128.58, 127.66, 127.14, 125.46, 123.24, 121.41, 118.77, 118.69, 112.01, 111.87, 44.09, 25.47. HRMS (ESI-MS) m/z: $[M+H]^+$ calcd for $C_{22}H_{18}ClN_3O_3S$: 440.0834; found: 440.0834.

(**5i**, *$C_{22}H_{18}ClN_3O_3S$*). Yellow solid; Yield 45%; m.p. 238.7 - 239.4 °C. ^{1}H NMR (500 MHz, DMSO-*d6*) δ 10.85 (s, 1H), 8.43 (t, *J* = 5.7 Hz, 1H), 7.98 (s, 1H), 7.76 (s,

1H), 7.61 (s, 1H), 7.60 (d, J = 2.5 Hz, 1H), 7.58 (s, 1H), 7.53 (d, J = 7.8 Hz, 1H), 7.34 (d, J = 8.1 Hz, 1H), 7.16 (d, J = 2.4 Hz, 1H), 7.06 (d, J = 7.7 Hz, 1H), 6.98 (t, J = 7.5 Hz, 1H), 4.28 (s, 2H), 3.37 - 3.33 (d, J = 6.8 Hz, 2H), 2.82 (d, J = 7.5 Hz, 2H). ^{13}C NMR (126 MHz, DMSO-*d6*) δ 167.29, 165.64, 165.27, 136.70, 135.58, 134.51, 132.19, 131.75, 130.83, 130.68, 128.28, 127.65, 123.52, 123.24, 121.41, 118.76, 118.69, 112.00, 111.86, 44.07, 25.47. HRMS (ESI-MS) m/z: $[M+H]^+$ calcd for $C_{22}H_{18}ClN_3O_3S$: 440.0834; found: 440.0835.

(**5j**, $C_{22}H_{18}ClN_3O_3S$). White solid; Yield 62%; m.p. 209.8 - 211.1 °C. ^{1}H NMR (500 MHz, DMSO-*d6*) δ 10.85 (s, 1H), 8.43 (t, J = 5.7 Hz, 1H), 7.98 (s, 1H), 7.69 (s, 1H), 7.68 (s, 1H), 7.64 (s, 1H), 7.63 (s, 1H), 7.62 - 7.59 (m, 1H), 7.53 (d, J = 7.8 Hz, 1H), 7.34 (d, J = 8.0 Hz, 1H), 7.16 (s, 1H), 7.09 - 7.04 (m, 1H), 6.98 (t, J = 7.4 Hz, 1H), 4.27 (s, 2H), 3.37 - 3.33 (d, J = 6.4 Hz, 2H), 2.83 (t, J = 7.4 Hz, 2H).^{13}C NMR (126 MHz, DMSO-*d6*) δ 167.38, 165.73, 165.30, 136.70, 135.84, 132.47, 132.30, 132.08, 130.00, 129.86, 127.65, 123.24, 122.47, 121.41, 118.76, 118.69, 112.01, 111.86, 44.05, 25.47. HRMS (ESI-MS) m/z: $[M+H]^+$ calcd for $C_{22}H_{18}ClN_3O_3S$: 440.0834; found: 440.0834.

(**5k**, $C_{22}H_{18}BrN_3O_3S$). Yellow solid; Yield 32%; m.p. 326.4 - 327.8 °C. ^{1}H NMR (500 MHz, DMSO-*d6*) δ 10.86 (s, 1H), 8.44 (t, J = 5.7 Hz, 1H), 8.03 (s, 1H), 7.84 (d, J = 7.0 Hz, 1H), 7.63 - 7.61 (m, 1H), 7.58 (d, J = 8.0 Hz, 1H), 7.53 (d, J = 7.8 Hz, 1H), 7.47 - 7.43 (m, 1H), 7.34 (d, J = 8.1 Hz, 1H), 7.16 (s, 1H), 7.06 (t, J = 7.0 Hz, 1H), 6.98 (t, J = 7.6 Hz, 1H), 4.28 (s, 2H), 3.38 - 3.34 (m, 2H), 2.83 (t, J = 7.4 Hz, 2H). ^{13}C NMR (126 MHz, DMSO-*d6*) δ 167.39, 165.39, 165.24, 136.69, 134.16, 133.14, 132.78, 131.55, 129.61, 129.23, 127.64, 125.77, 125.48, 123.23, 121.40, 118.75, 118.68, 111.99, 111.85, 44.09, 25.46. HRMS (ESI-MS) m/z: $[M+H]^+$ calcd for $C_{22}H_{18}BrN_3O_3S$: 486.0307; found: 486.0307.

(**5l**, $C_{22}H_{18}BrN_3O_3S$). Yellow solid; Yield 46%; m.p. 253.5 - 254.7 °C. ^{1}H NMR (500 MHz, DMSO-*d6*) δ 10.84 (s, 1H), 8.42 (t, J = 5.8 Hz, 1H), 7.97 (s, 1H), 7.89 (s, 1H), 7.71 (d, J = 8.1 Hz, 1H), 7.64 (d, J = 7.8 Hz, 1H), 7.53 (d, J = 5.2 Hz, 1H), 7.51 (d, J = 5.3 Hz, 1H), 7.34 (d, J = 8.1 Hz, 1H), 7.16 (s, 1H), 7.06 (t, J = 7.5 Hz, 1H), 6.98 (t, J = 7.5 Hz, 1H), 4.28 (s, 2H), 3.37 - 3.34 (m, 2H), 2.83 (t, J = 7.5 Hz, 2H).^{13}C

NMR (126 MHz, DMSO-*d6*) δ 167.25, 165.59, 165.26, 136.69, 136.01, 135.80, 133.68, 133.54, 133.32, 133.26, 132.12, 131.92, 131.81, 130.43, 128.59, 128.56, 127.64, 123.45, 123.23, 123.01, 122.92, 121.40, 118.75, 118.68, 112.00, 111.85, 44.07, 25.47. HRMS (ESI-MS) m/z: $[M+H]^+$ calcd for $C_{22}H_{18}BrN_3O_3S$: 486.0307; found: 486.0307.

(**5m**, *$C_{22}H_{18}BrN_3O_3S$*). Yellow solid; Yield 52%; m.p. 245.4 - 246.8 °C. ^{1}H NMR (500 MHz, DMSO-*d6*) δ 10.85 (s, 1H), 8.42 (t, *J* = 5.7 Hz, 1H), 7.96 (s, 1H), 7.74 (d, *J* = 8.5 Hz, 1H), 7.62 (s, 1H), 7.60 (s, 1H), 7.54 (t, *J* = 8.5 Hz, 2H), 7.34 (d, *J* = 8.0 Hz, 1H), 7.16 (s, 1H), 7.06 (t, *J* = 7.5 Hz, 1H), 6.98 (t, *J* = 7.4 Hz, 1H), 4.27 (s, 2H), 3.37 - 3.33 (m, 2H), 2.83 (t, *J* = 7.4 Hz, 2H). ^{13}C NMR (126 MHz, DMSO-*d6*) δ 167.35, 165.72, 165.28, 136.68, 132.91, 132.81, 132.79, 132.61, 132.56, 132.42, 132.25, 130.82, 127.64, 124.78, 124.33, 123.23, 122.53, 121.40, 118.75, 118.67, 111.99, 111.85, 44.04, 25.47. HRMS (ESI-MS) m/z: $[M+H]^+$ calcd for $C_{22}H_{18}BrN_3O_3S$: 486.0307; found: 486.0307.

(***5n***, *$C_{23}H_{18}F_3N_3O_3S$*). Yellow solid; Yield 32%; m.p. 206.0 - 208.1 °C. ^{1}H NMR (500 MHz, DMSO-*d*$_6$) *δ* 10.85 (s, 1H), 8.45 (t, *J* = 5.7 Hz, 1H), 8.01 (s, 1H), 7.93 (d, *J* = 7.9 Hz, 1H), 7.87 (d, *J* = 7.6 Hz, 1H), 7.78 (d, *J* = 7.8 Hz, 1H), 7.72 (t, *J* = 7.7 Hz, 1H), 7.53 (d, *J* = 7.9 Hz, 1H), 7.34 (d, *J* = 8.1 Hz, 1H), 7.16 (s, 1H), 7.07 (t, *J* = 7.6 Hz, 1H), 6.98 (t, *J* = 7.6 Hz, 1H), 4.28 (s, 2H), 3.38 - 3.34 (m, 2H), 2.84 (t, *J* = 7.5 Hz, 2H). ^{13}C NMR (126 MHz, DMSO-*d*$_6$) *δ* 167.29, 165.19, 136.69, 134.04, 131.65, 131.17, 129.58, 128.29, 127.64, 127.43, 127.39, 127.15, 123.23, 121.40, 118.75, 118.68, 111.98, 111.85, 44.11, 25.46. HRMS (ESI-MS) m/z: $[M+H]^+$ calcd for $C_{23}H_{18}F_3N_3O_3S$: 474.1098; found: 474.1098.

(**5o**, *$C_{23}H_{18}F_3N_3O_3S$*). Yellow solid; Yield 41%; m.p. 194.0 - 195.1 °C. ^{1}H NMR (500 MHz, DMSO-*d6*) δ 10.86 (s, 1H), 8.44 (t, *J* = 5.7 Hz, 1H), 8.11 (s, 1H), 8.07 (s, 1H), 7.93 (d, *J* = 7.9 Hz, 1H), 7.88 (d, *J* = 7.8 Hz, 1H), 7.81 (t, *J* = 7.9 Hz, 1H), 7.53 (d, *J* = 7.8 Hz, 1H), 7.34 (d, *J* = 8.1 Hz, 1H), 7.16 (s, 1H), 7.07 (t, *J* = 7.7 Hz, 1H), 6.98 (t, *J* = 7.4 Hz, 1H), 4.29 (s, 2H), 3.38 - 3.34 (m, 2H), 2.83 (t, *J* = 7.4 Hz, 2H). ^{13}C NMR (126 MHz, DMSO-*d6*) δ 167.18, 165.58, 165.25, 136.69, 134.55, 133.23, 132.12, 131.08, 127.76, 127.73, 127.64, 123.92, 123.23, 121.40, 118.75, 118.68,

111.99, 111.85, 44.09, 25.47. HRMS (ESI-MS) m/z: $[M+H]^+$ calcd for $C_{23}H_{18}F_3N_3O_3S$: 474.1098; found: 474.1097.

(**5p**, *$C_{23}H_{18}F_3N_3O_3S$*). Yellow solid; Yield 51%; m.p. 323.8 - 324.7 °C. ^{1}H NMR (500 MHz, DMSO-*d6*) δ 10.86 (s, 1H), 8.44 (t, *J* = 5.7 Hz, 1H), 8.06 (s, 1H), 7.91 (d, *J* = 8.4 Hz, 2H), 7.87 (d, *J* = 3.7 Hz, 3H), 7.86 (d, *J* = 4.5 Hz, 2H), 7.80 (d, *J* = 8.2 Hz, 1H), 7.53 (d, *J* = 7.8 Hz, 1H), 7.34 (d, *J* = 8.0 Hz, 1H), 7.16 (d, *J* = 2.3 Hz, 1H), 7.07 (t, *J* = 7.5 Hz, 1H), 6.98 (t, *J* = 7.4 Hz, 1H), 4.29 (s, 2H), 3.38 - 3.34 (d, *J* = 6.4 Hz, 3H), 2.84 (t, *J* = 7.4 Hz, 2H). ^{13}C NMR (126 MHz, DMSO-*d6*) δ 167.27, 165.59, 165.24, 137.36, 136.69, 131.94, 131.12, 130.92, 127.64, 126.68, 126.65, 126.56, 126.53, 124.69, 123.23, 121.40, 118.75, 118.67, 111.99, 111.85, 44.11, 25.47. HRMS (ESI-MS) m/z: $[M+H]^+$ calcd for $C_{23}H_{18}F_3N_3O_3S$: 474.1098; found: 474.1099.

(**5q**, *$C_{23}H_{21}N_3O_4S$*). Yellow solid; Yield 51%; m.p. 239.7 - 340.7 °C. ^{1}H NMR (500 MHz, DMSO-*d6*) δ 10.84 (s, 1H), 8.41 (t, *J* = 5.7 Hz, 1H), 8.11 (s, 1H), 7.54 (s, 1H), 7.51 (d, *J* = 8.5 Hz, 1H), 7.48 (d, *J* = 7.8 Hz, 1H), 7.34 (d, *J* = 8.0 Hz, 1H), 7.18 (d, *J* = 8.4 Hz, 1H), 7.15 (d, *J* = 9.3 Hz, 1H), 7.12 (d, *J* = 7.4 Hz, 1H), 7.07 (t, *J* = 7.5 Hz, 1H), 6.98 (t, *J* = 7.4 Hz, 1H), 4.26 (s, 2H), 3.91 (s, 3H), 3.37 - 3.34 (m, 2H), 2.83 (t, *J* = 7.3 Hz, 2H). ^{13}C NMR (126 MHz, DMSO-*d6*) δ 167.90, 165.95, 165.41, 158.54, 136.71, 133.29, 129.43, 128.71, 127.67, 123.24, 121.79, 121.55, 121.43, 118.78, 118.71, 112.45, 112.03, 111.87, 56.27, 43.93, 25.48. HRMS (ESI-MS) m/z: $[M+H]^+$ calcd for $C_{23}H_{21}N_3O_4S$: 436.1330; found: 436.1330.

(**5r**, *$C_{23}H_{21}N_3O_4S$*). Yellow solid; Yield 61%; m.p. 284.0 - 285.0 °C. ^{1}H NMR (500 MHz, DMSO-*d6*) δ 10.85 (s, 1H), 8.43 (t, *J* = 5.7 Hz, 1H), 7.96 (s, 1H), 7.53 (d, *J* = 7.9 Hz, 1H), 7.48 (t, *J* = 8.2 Hz, 1H), 7.34 (d, *J* = 8.1 Hz, 1H), 7.23 (s, 1H), 7.22 (d, *J* = 4.9 Hz, 1H), 7.16 (s, 1H), 7.11 - 7.08 (m, 1H), 7.06 (d, *J* = 7.7 Hz, 1H), 6.98 (t, *J* = 7.4 Hz, 1H), 4.27 (s, 2H), 3.82 (s, 3H), 3.38 - 3.33 (m, 2H), 2.83 (t, *J* = 7.4 Hz, 2H). ^{13}C NMR (126 MHz, DMSO-*d6*) δ 167.59, 165.79, 165.35, 160.16, 136.70, 134.75, 133.75, 131.05, 127.65, 123.24, 122.43, 122.07, 121.42, 118.77, 118.69, 117.09, 116.01, 112.02, 111.87, 55.79, 43.99, 25.46. HRMS (ESI-MS) m/z: $[M+H]^+$ calcd for $C_{23}H_{21}N_3O_4S$: 436.1330; found: 436.1330.

(**5s**, *$C_{23}H_{21}N_3O_4S$*). Yellow solid; Yield 68%; m.p. 193.0 - 194.1 °C. ^{1}H NMR (500 MHz, DMSO-*d6*) δ 10.85 (s, 1H), 8.41 (t, *J* = 5.7 Hz, 1H), 7.93 (s, 1H), 7.64 (s, 1H), 7.63 (d, *J* = 2.2 Hz, 1H), 7.53 (d, *J* = 7.9 Hz, 1H), 7.34 (d, *J* = 8.1 Hz, 1H), 7.16 (s, 1H), 7.14 (s, 1H), 7.12 (d, *J* = 2.1 Hz, 1H), 7.06 (d, *J* = 1.2 Hz, 1H), 7.01 - 6.96 (m, 1H), 4.26 (s, 2H), 3.84 (s, 3H), 3.39 - 3.35 (m, 2H), 2.83 (t, *J* = 7.4 Hz, 2H). ^{13}C NMR (126 MHz, DMSO-*d6*) δ 167.71, 165.96, 165.43, 161.73, 136.71, 133.78, 132.82, 132.57, 127.66, 125.87, 123.24, 121.41, 118.77, 118.69, 118.39, 115.51, 115.39, 112.03, 111.86, 56.01, 55.96, 43.92, 25.48. HRMS (ESI-MS) m/z: $[M+H]^+$ calcd for $C_{23}H_{21}N_3O_4S$: 436.1330; found: 436.1330.

(**5t**, *$C_{22}H_{19}N_3O_4S$*). Yellow solid; Yield 34%; m.p. 214.2 - 216.2 °C. ^{1}H NMR (500 MHz, DMSO-*d6*) δ 10.86 (s, 1H), 10.63 (s, 1H), 8.42 (t, *J* = 5.7 Hz, 1H), 8.16 (s, 1H), 7.53 (d, *J* = 7.8 Hz, 1H), 7.39 (d, *J* = 6.3 Hz, 1H), 7.35 (d, *J* = 7.4 Hz, 1H), 7.33 (s, 1H), 7.16 (d, *J* = 2.3 Hz, 1H), 7.07 (t, *J* = 7.0 Hz, 1H), 6.99 (d, *J* = 5.0 Hz, 1H), 6.98 (s, 1H), 6.97 (d, *J* = 7.5 Hz, 1H), 4.26 (s, 2H), 3.38 - 3.34 (m, 2H), 2.84 (t, *J* = 7.4 Hz, 2H). ^{13}C NMR (126 MHz, DMSO-*d6*) δ 167.95, 166.06, 165.43, 157.87, 136.70, 133.13, 129.08, 127.66, 123.24, 121.41, 120.29, 120.18, 118.77, 118.70, 116.71, 112.02, 111.86, 43.89, 25.47. HRMS (ESI-MS) m/z: $[M+H]^+$ calcd for $C_{22}H_{19}N_3O_4S$: 422.1173; found: 422.1173.

(**5u**, *$C_{22}H_{19}N_3O_4S$*). White solid; Yield 45%; m.p. 257.9 - 258.5 °C. ^{1}H NMR (500 MHz, DMSO-*d6*) δ 10.85 (s, 1H), 9.90 (s, 1H), 8.42 (t, *J* = 5.8 Hz, 1H), 7.87 (s, 1H), 7.53 (d, *J* = 7.9 Hz, 1H), 7.36 (d, *J* = 7.9 Hz, 1H), 7.34 (d, *J* = 6.9 Hz, 1H), 7.16 (s, 1H), 7.09 (d, *J* = 8.7 Hz, 1H), 7.06 (d, *J* = 7.8 Hz, 1H), 7.03 (s, 1H), 6.98 (t, *J* = 7.6 Hz, 1H), 6.91 (d, *J* = 8.2 Hz, 1H), 4.27 (s, 2H), 3.37 - 3.37 (m, 2H), 2.84 (d, *J* = 7.5 Hz, 2H). ^{13}C NMR (126 MHz, DMSO-*d6*) δ 167.65, 165.81, 165.34, 158.41, 136.69, 134.55, 133.97, 130.99, 127.64, 123.23, 121.92, 121.47, 121.40, 118.75, 118.68, 118.54, 116.49, 112.01, 111.85, 43.97, 25.47. HRMS (ESI-MS) m/z: $[M+H]^+$ calcd for $C_{22}H_{19}N_3O_4S$: 422.1173; found: 422.1173.

(**5v**, *$C_{22}H_{19}N_3O_4S$*). White solid; Yield 46%; m.p. 265.7 - 267.1 °C. ^{1}H NMR (500 MHz, DMSO-*d6*) δ 10.85 (s, 1H), 10.39 (s, 1H), 8.40 (t, *J* = 5.7 Hz, 1H), 7.87 (s, 1H), 7.53 (s, 2H), 7.52 (s, 3H), 7.51 (s, 2H), 7.33 (d, *J* = 8.0 Hz, 1H), 7.16 (d, *J* = 2.4

Hz, 1H), 7.06 (t, *J* = 7.6 Hz, 1H), 6.98 (d, *J* = 7.5 Hz, 1H), 6.95 (d, *J* = 8.4 Hz, 2H), 6.93 (d, *J* = 6.5 Hz, 2H), 4.25 (s, 2H), 3.36 - 3.32 (m, 2H), 2.82 (t, *J* = 7.5 Hz, 2H). ^{13}C NMR (126 MHz, DMSO-*d6*) δ 167.81, 166.03, 165.46, 160.70, 136.70, 134.21, 133.15, 127.66, 124.32, 123.23, 121.41, 118.76, 118.69, 117.09, 116.92, 112.03, 111.85, 43.88, 25.47. HRMS (ESI-MS) m/z: $[M+H]^+$ calcd for $C_{22}H_{19}N_3O_4S$: 422.1173; found: 422.1173.

(**5w**, *$C_{23}H_{18}N_4O_3S$*). White solid; Yield 41%; m.p. 236.5 - 239.7 °C. ^{1}H NMR (500 MHz, DMSO-*d6*) δ 10.85 (s, 1H), 8.44 (t, *J* = 5.7 Hz, 1H), 8.14 (s, 1H), 7.97 (d, *J* = 7.8 Hz, 1H), 7.94 (d, *J* = 7.9 Hz, 1H), 7.85 (dd, *J* = 14.2, 7.8 Hz, 1H), 7.77 (t, *J* = 7.9 Hz, 1H), 7.58 (s, 1H), 7.53 (d, *J* = 7.9 Hz, 1H), 7.34 (d, *J* = 8.1 Hz, 1H), 7.16 (s, 1H), 7.06 (t, *J* = 7.5 Hz, 1H), 6.98 (t, *J* = 7.4 Hz, 1H), 4.28 (s, 2H), 3.38 - 3.34 (m, 2H), 2.83 (t, *J* = 7.4 Hz, 2H). ^{13}C NMR (126 MHz, DMSO-*d6*) δ 167.20, 165.58, 165.23, 136.68, 134.65, 134.59, 134.17, 133.86, 133.69, 133.49, 132.60, 131.48, 131.10, 130.73, 127.63, 124.32, 123.24, 121.40, 118.88, 118.75, 118.67, 118.60, 113.03, 112.64, 111.98, 111.85, 44.10, 25.46. HRMS (ESI-MS) m/z: $[M+H]^+$ calcd for $C_{23}H_{18}N_4O_3S$: 431.1177; found: 431.1177.

(**5x**, *$C_{23}H_{18}N_4O_3S$*). Yellow solid; Yield 52%; m.p. 250.7 - 252.7 °C. ^{1}H NMR (500 MHz, DMSO-*d6*) δ 10.85 (s, 1H), 8.44 (t, *J* = 5.7 Hz, 1H), 8.04 (s, 1H), 8.02 (s, 1H), 8.00 (s, 1H), 7.84 (s, 1H), 7.83 (s, 1H), 7.53 (d, *J* = 7.9 Hz, 1H), 7.34 (d, *J* = 8.1 Hz, 1H), 7.16 (s, 1H), 7.06 (t, *J* = 7.5 Hz, 1H), 6.98 (t, *J* = 7.4 Hz, 1H), 4.28 (s, 2H), 3.37 - 3.33 (m, 2H), 2.83 (t, *J* = 7.4 Hz, 2H). ^{13}C NMR (126 MHz, DMSO-*d6*) δ 167.14, 165.55, 165.22, 137.81, 136.68, 133.58, 131.70, 131.03, 127.64, 125.28, 123.23, 121.41, 118.84, 118.76, 118.67, 112.82, 111.99, 111.85, 44.13, 25.46. HRMS (ESI-MS) m/z: $[M+H]^+$ calcd for $C_{23}H_{18}N_4O_3S$: 431.1177; found: 431.1177.

(**5y**, *$C_{22}H_{18}N_4O_5S$*). Yellow solid; Yield 31%; m.p. 208.4 - 209.7 °C. ^{1}H NMR (500 MHz, DMSO-*d6*) δ 10.85 (s, 1H), 8.52 (t, *J* = 2.0 Hz, 1H), 8.44 (t, *J* = 5.7 Hz, 1H), 8.33 (d, *J* = 8.3 Hz, 1H), 8.15 (s, 1H), 8.07 (d, *J* = 7.7 Hz, 1H), 7.85 (t, *J* = 8.0 Hz, 1H), 7.53 (d, *J* = 7.9 Hz, 1H), 7.33 (d, *J* = 8.0 Hz, 1H), 7.16 (s, 1H), 7.06 (t, *J* = 7.5 Hz, 1H), 7.00 - 6.96 (m, 1H), 4.29 (s, 2H), 3.38 - 3.34 (m, 2H), 2.83 (t, *J* = 7.4 Hz, 2H). ^{13}C NMR (126 MHz, DMSO-*d6*) δ 167.04, 165.51, 165.22, 148.77, 136.69,

135.87, 135.05, 131.51, 131.47, 127.64, 125.26, 125.21, 124.64, 123.23, 121.40, 118.75, 118.67, 111.99, 111.85, 44.14, 25.47. HRMS (ESI-MS) m/z: $[M+H]^+$ calcd for $C_{22}H_{18}N_4O_5S$: 451.1075; found: 451.1075.

(**5z**, *$C_{22}H_{18}N_4O_5S$*). Brown solid; Yield 28%; m.p. 278.1 - 279.3 °C. ^1H NMR (500 MHz, DMSO-*d6*) δ 10.84 (s, 1H), 8.43 (t, *J* = 5.7 Hz, 1H), 8.37 (d, *J* = 8.5 Hz, 2H), 8.10 (s, 1H), 7.94 (s, 1H), 7.92 (s, 1H), 7.53 (d, *J* = 7.9 Hz, 1H), 7.34 (d, *J* = 8.1 Hz, 1H), 7.16 (s, 1H), 7.06 (t, *J* = 7.5 Hz, 1H), 6.98 (t, *J* = 7.4 Hz, 1H), 4.29 (s, 2H), 3.38 - 3.33 (m, 2H), 2.83 (t, *J* = 7.4 Hz, 2H). ^{13}C NMR (126 MHz, DMSO-*d6*) δ 167.12, 165.51, 165.20, 148.17, 139.64, 136.69, 131.57, 131.22, 127.64, 126.02, 124.84, 123.23, 121.40, 118.75, 118.67, 111.99, 111.85, 44.16, 25.46. HRMS (ESI-MS) m/z: $[M+H]^+$ calcd for $C_{22}H_{18}$ N_4O_5S: 451.1075; found: 451.1075.

2. ^{1}H NMR and ^{13}C NMR of compounds **5a~5z**

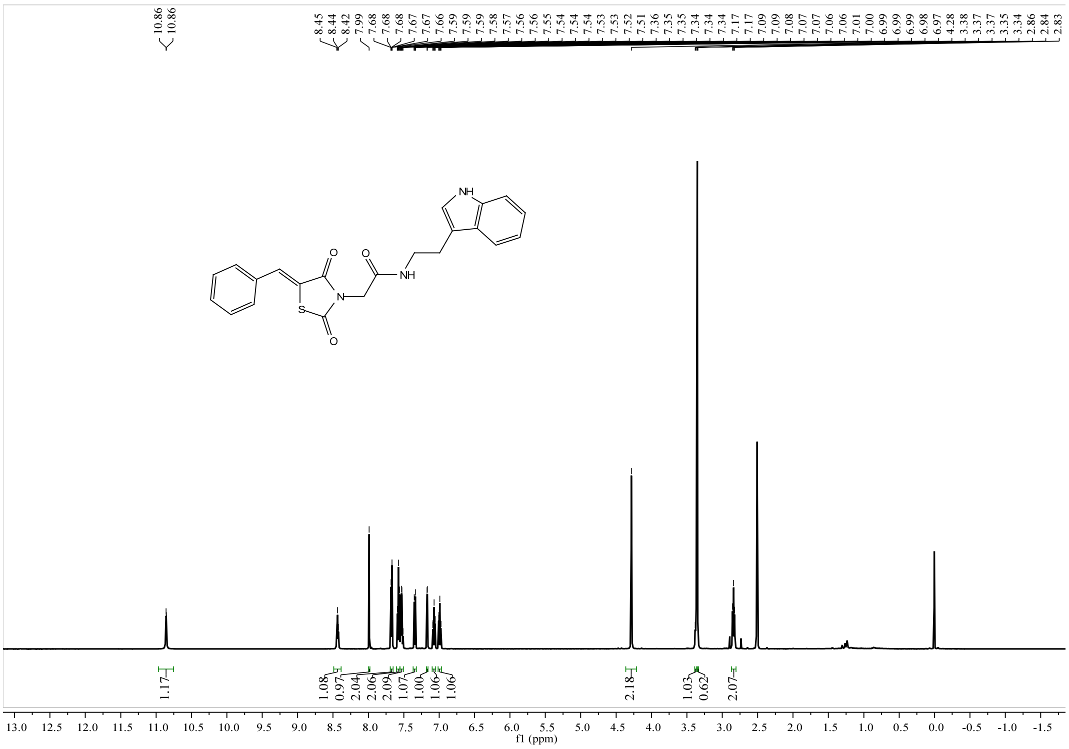

^{1}H NMR of compound **5a**

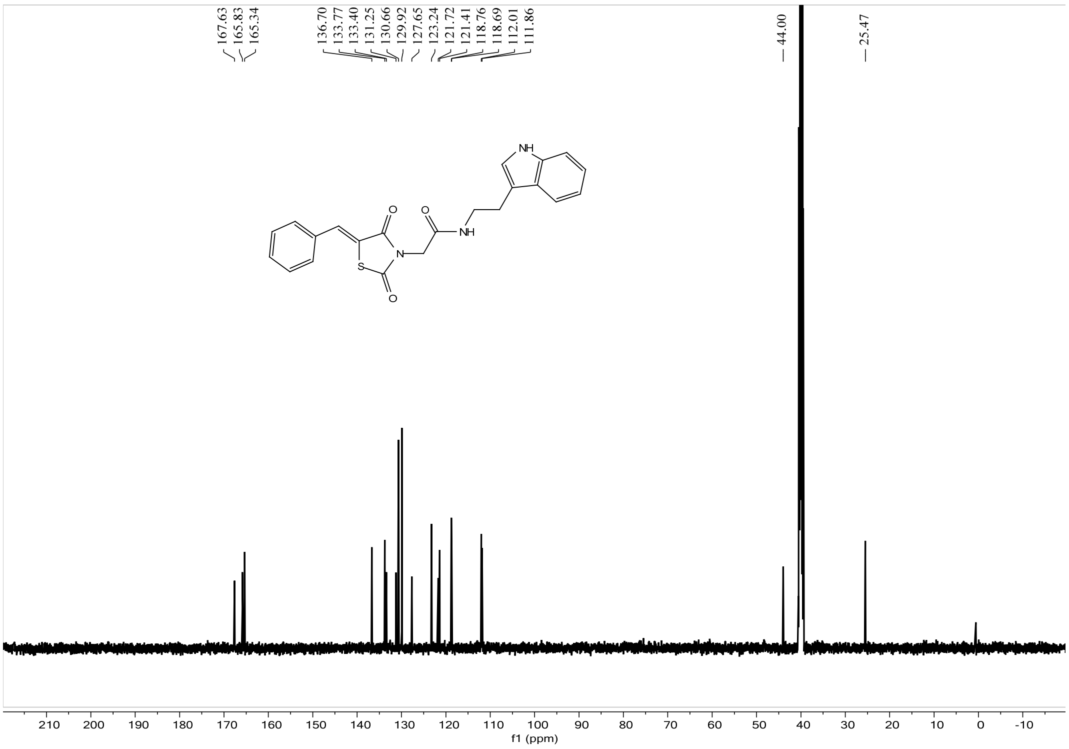

^{13}C NMR of compound **5a**

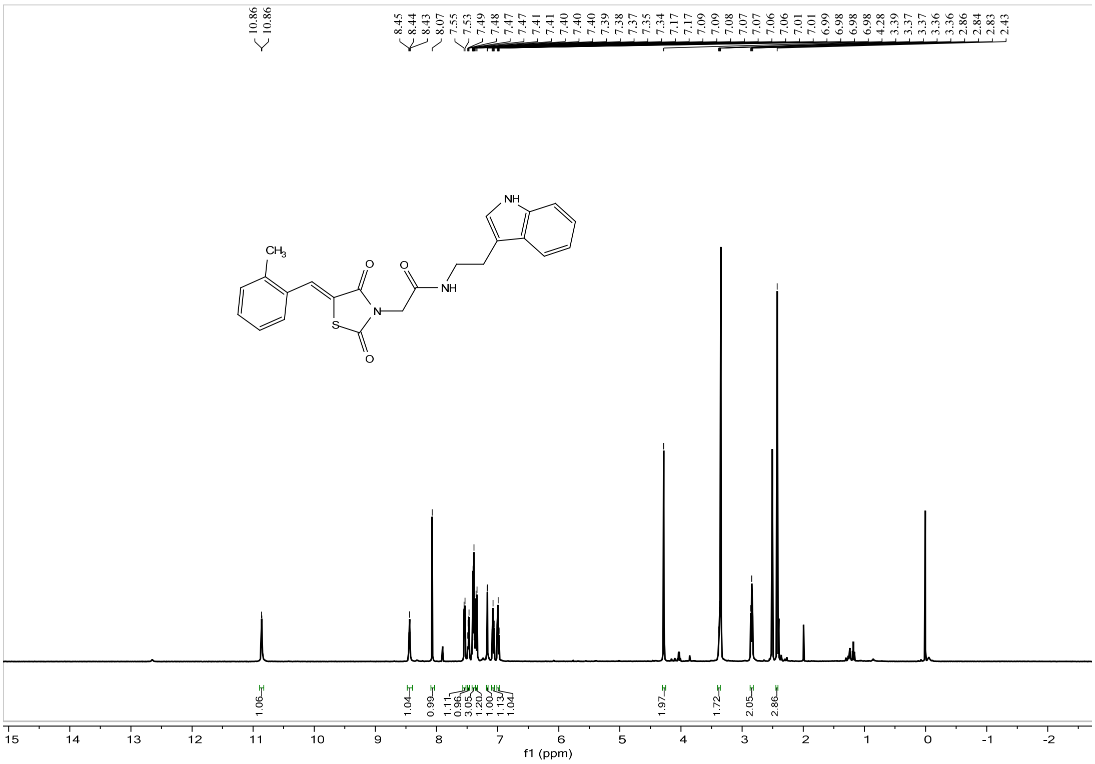

^{1}H NMR of compound **5b**

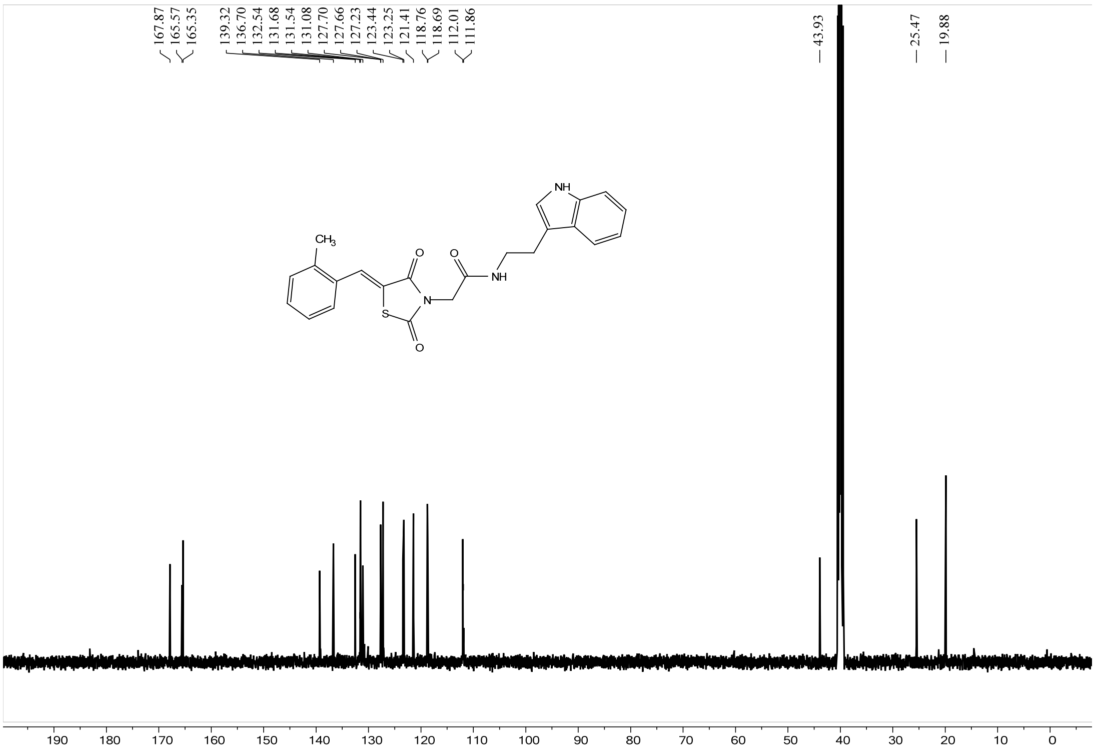

^{13}C NMR of compound **5b**

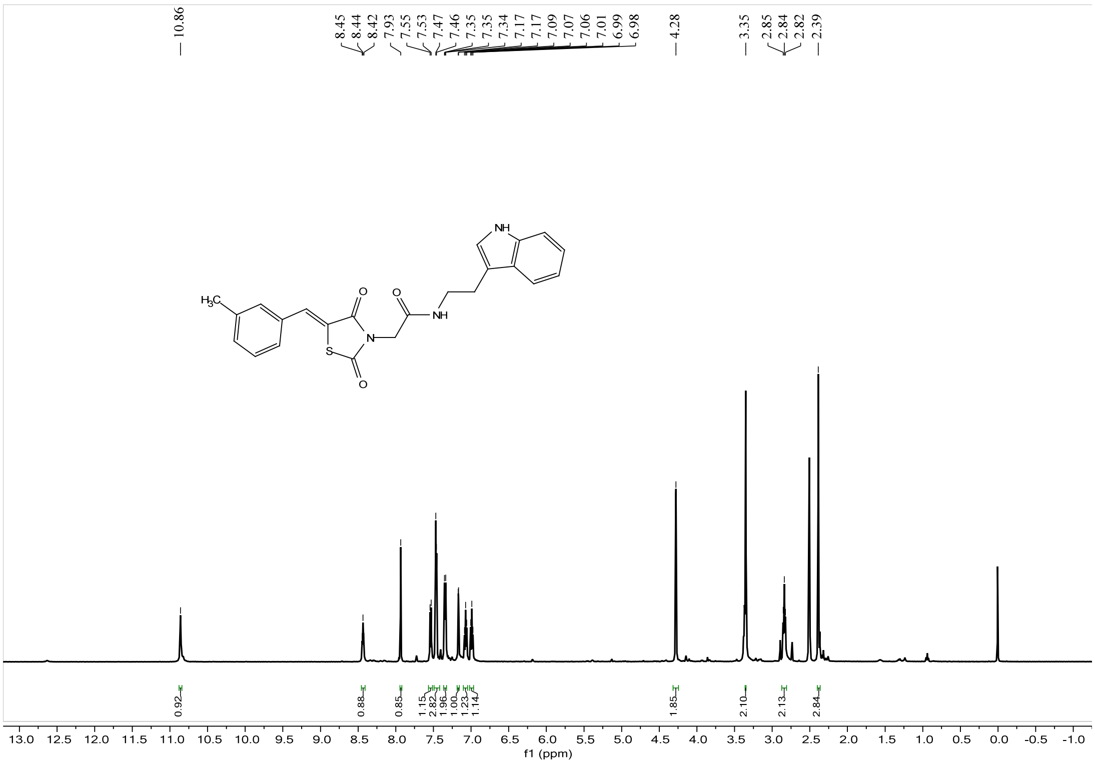

^{1}H NMR of compound **5c**

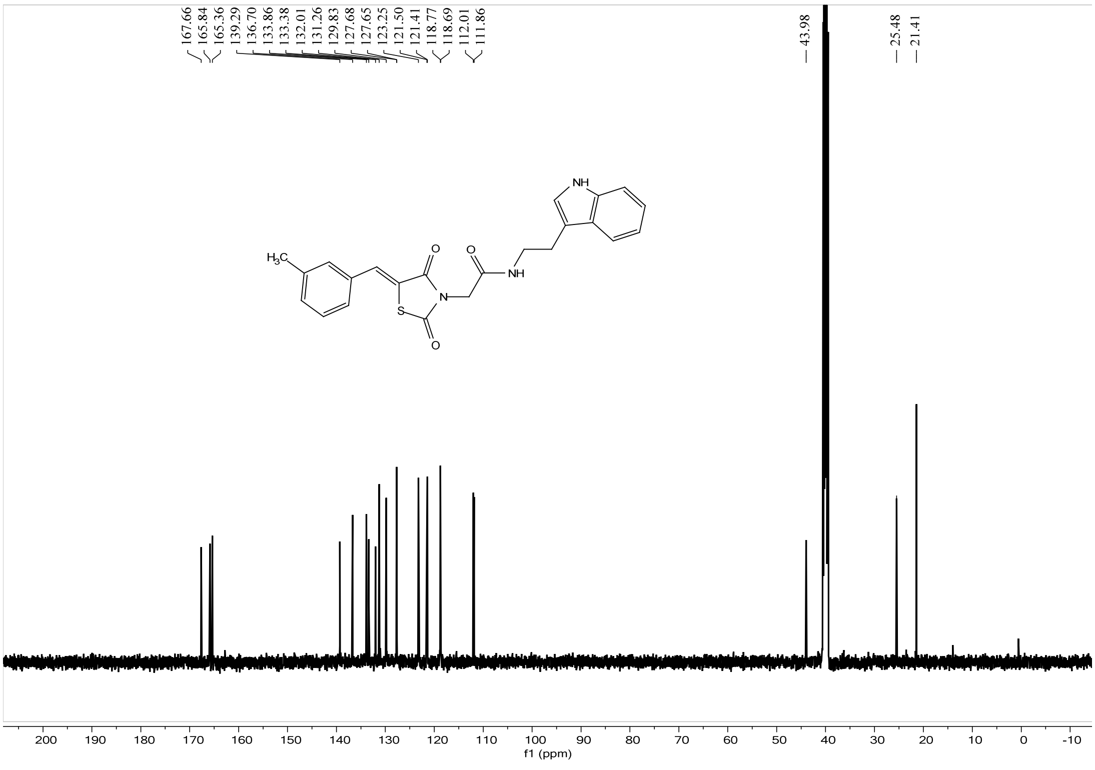

^{13}C NMR of compound **5c**

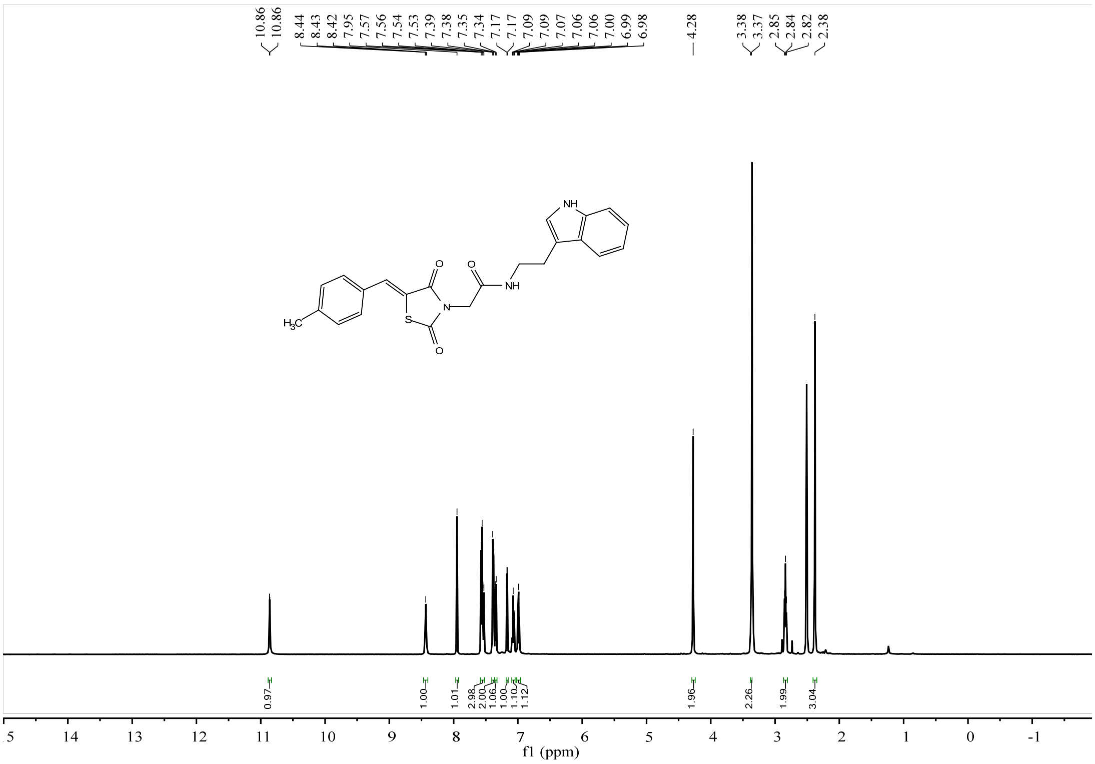

^{1}H NMR of compound **5d**

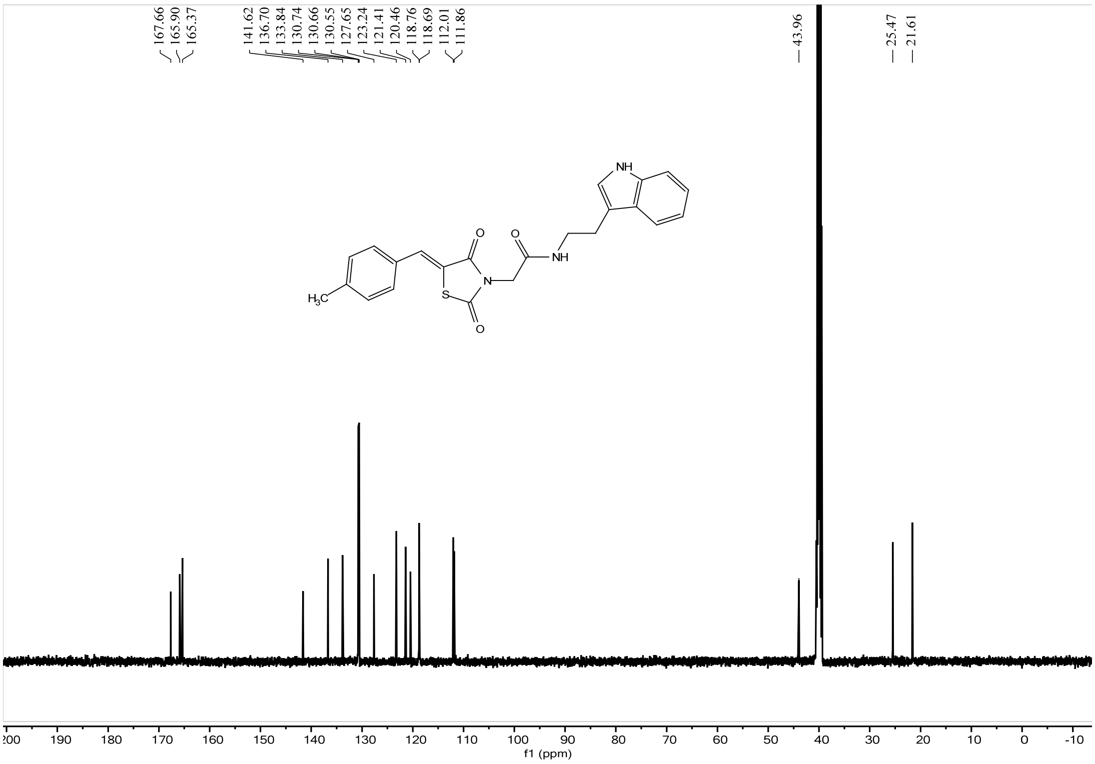

^{13}C NMR of compound **5d**

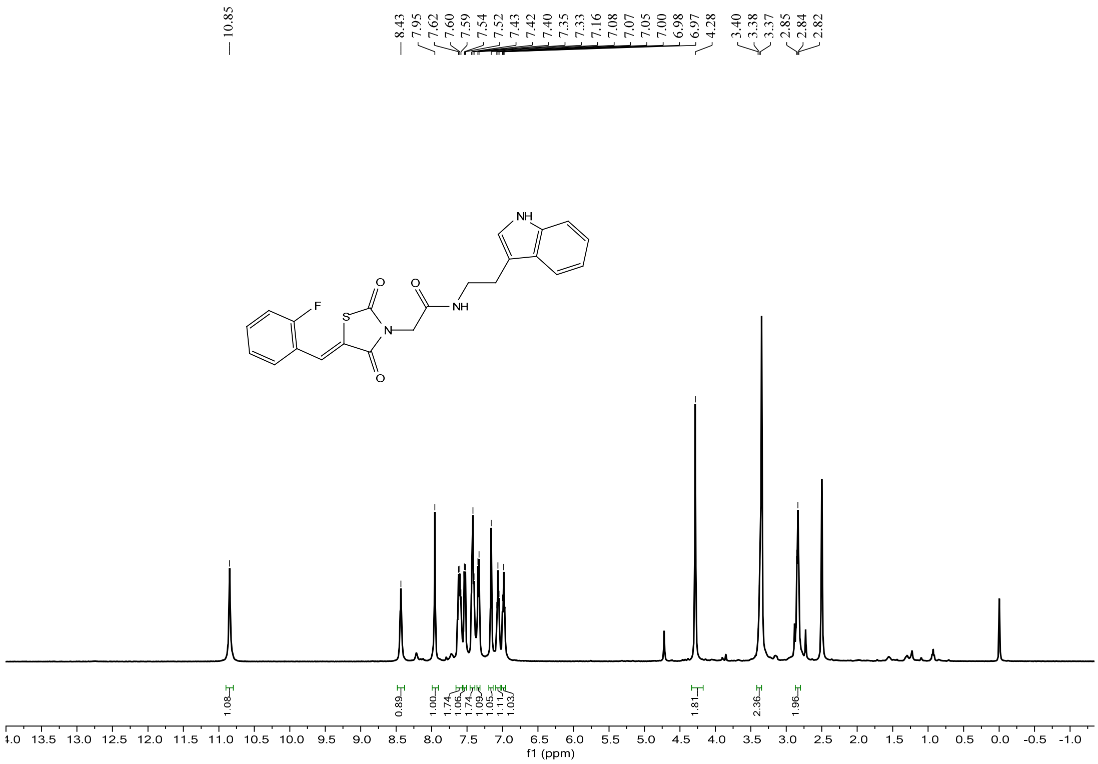

^{1}H NMR of compound **5e**

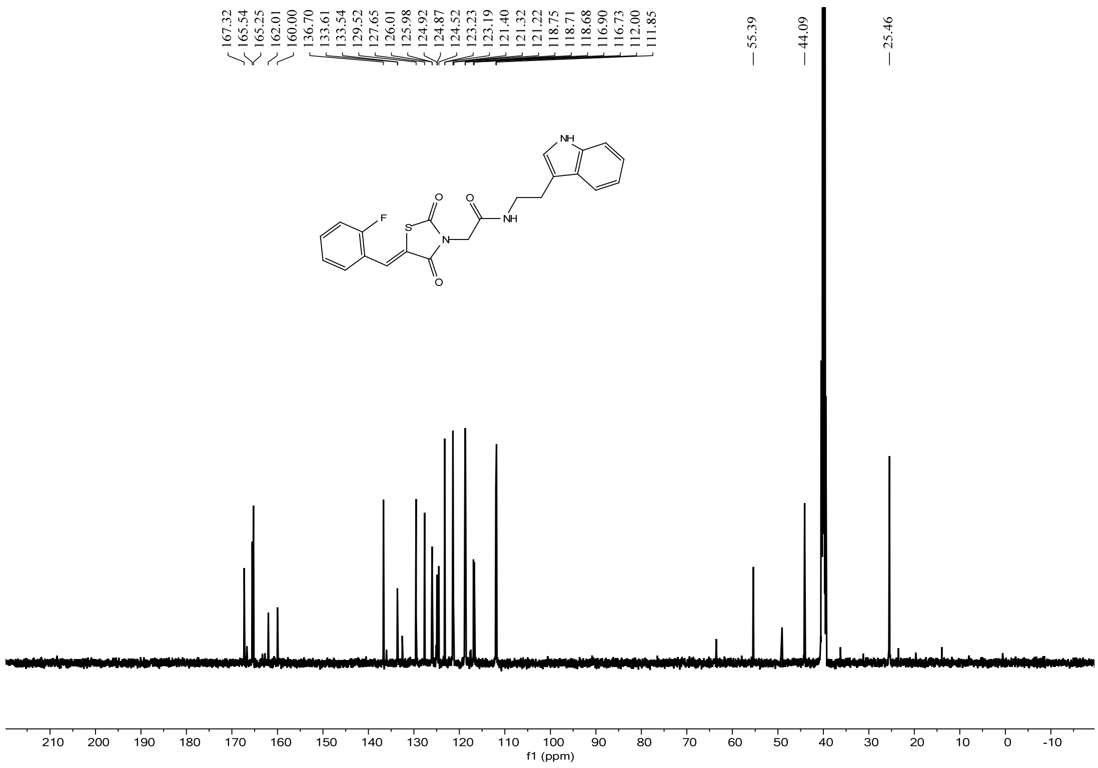

^{13}C NMR of compound **5e**

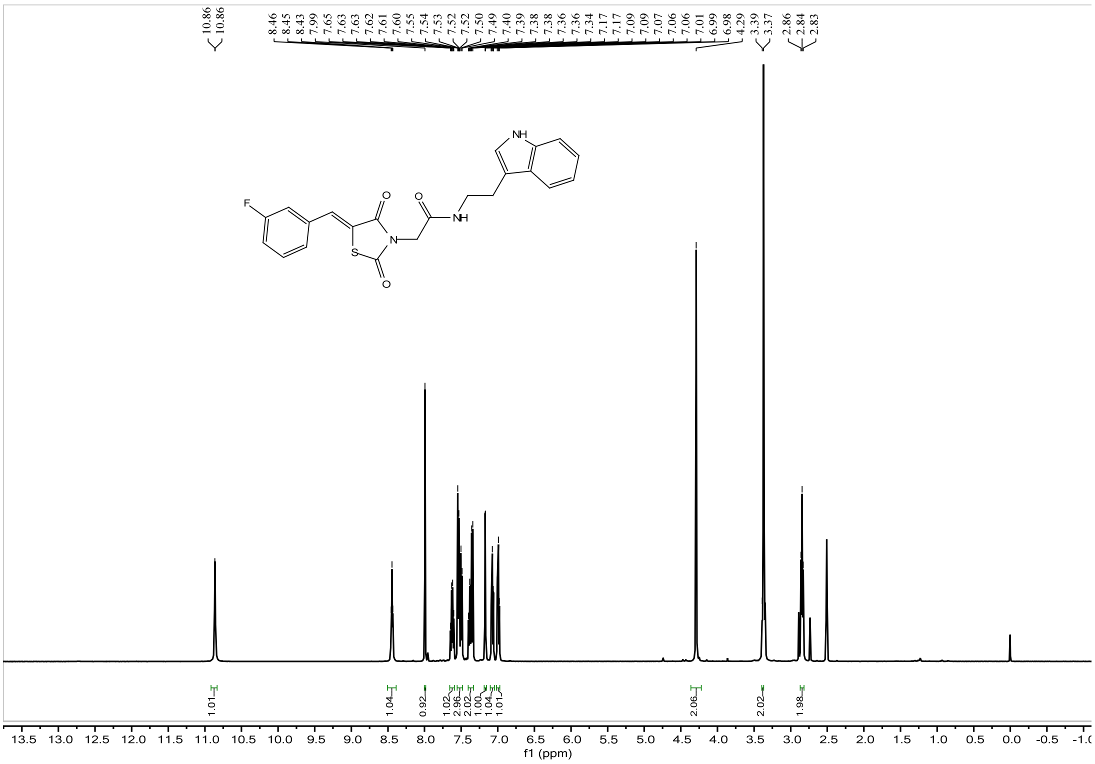

^{1}H NMR of compound **5f**

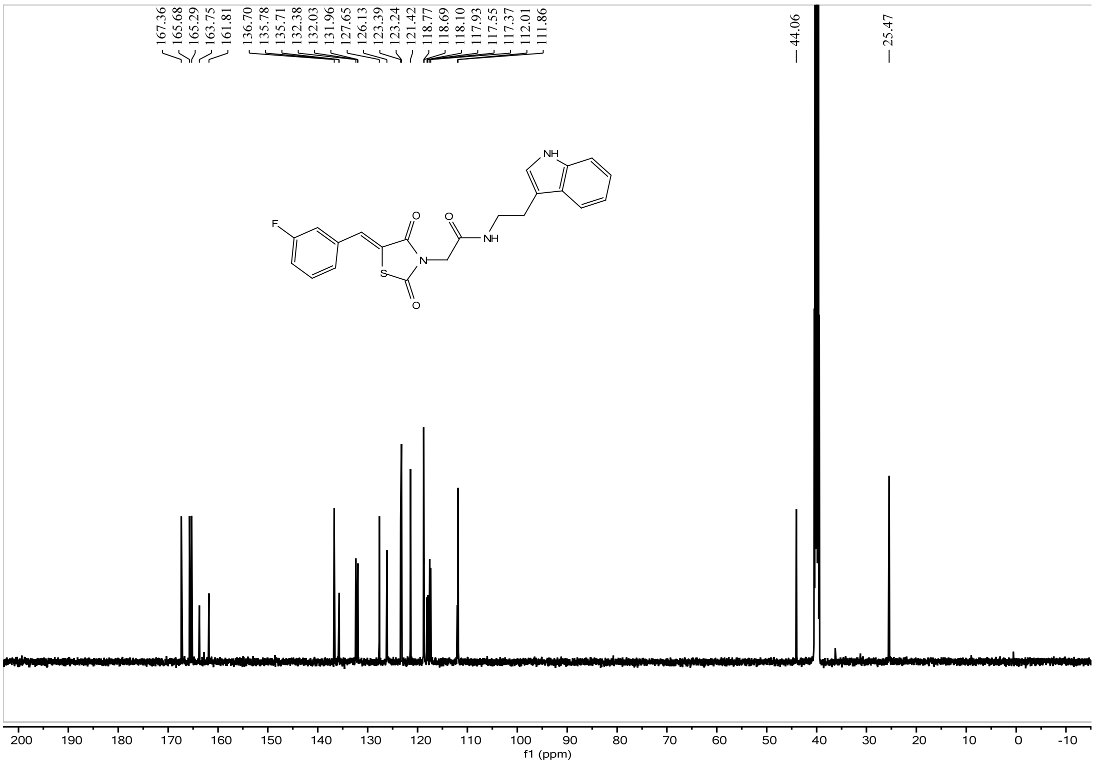

^{13}C NMR of compound **5f**

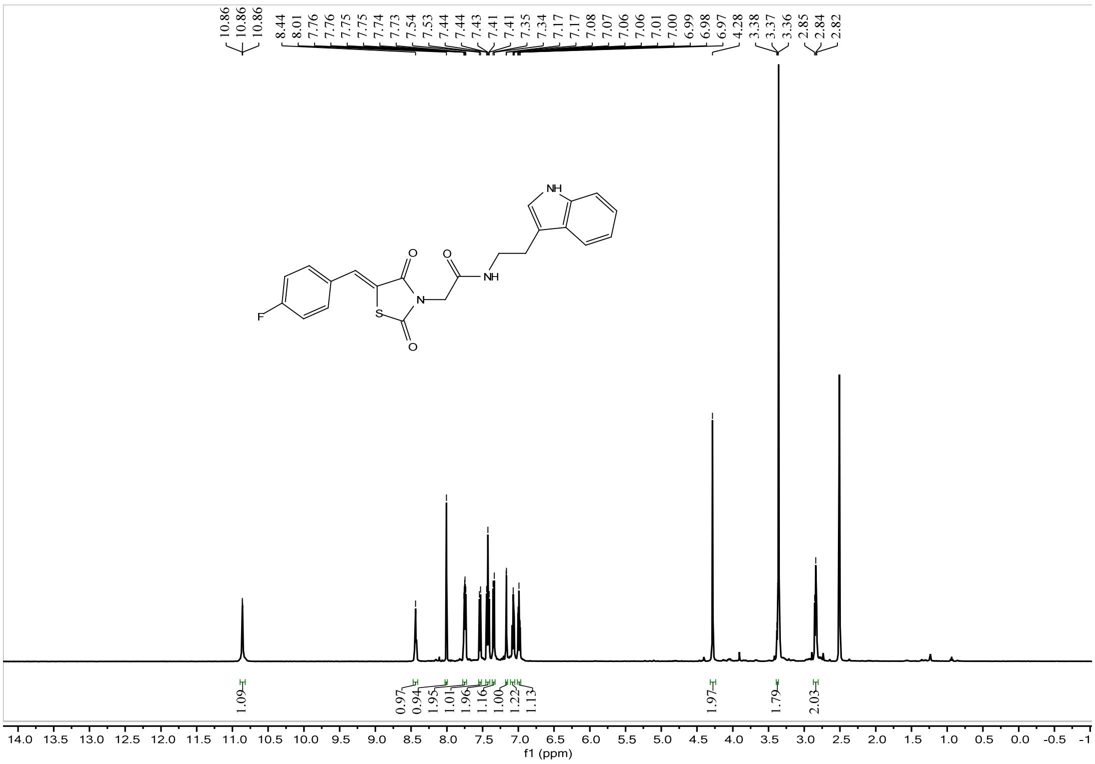

^{1}H NMR of compound **5g**

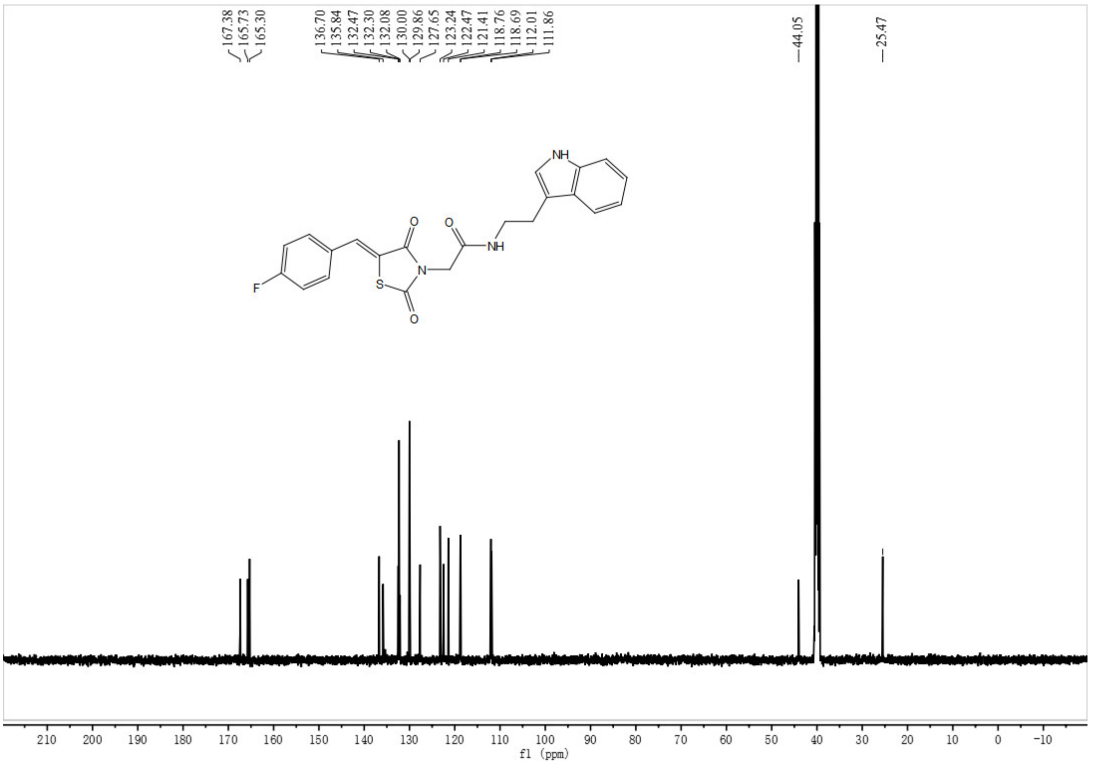

^{13}C NMR of compound **5g**

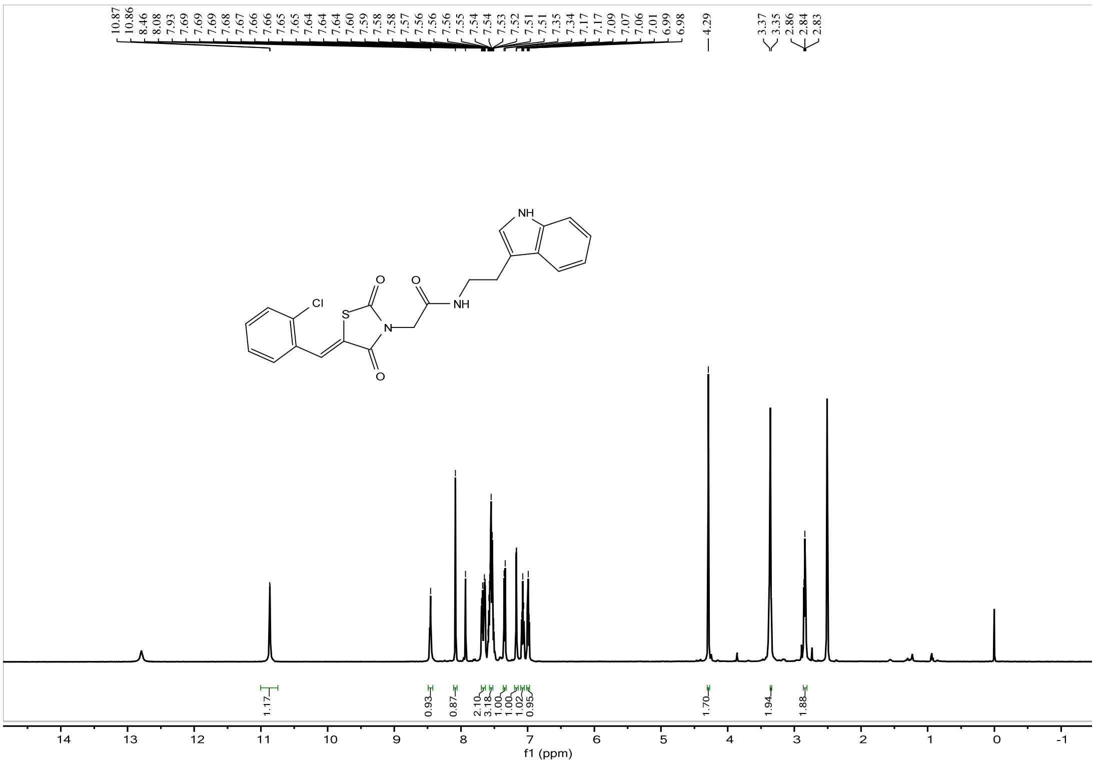

^{1}H NMR of compound **5h**

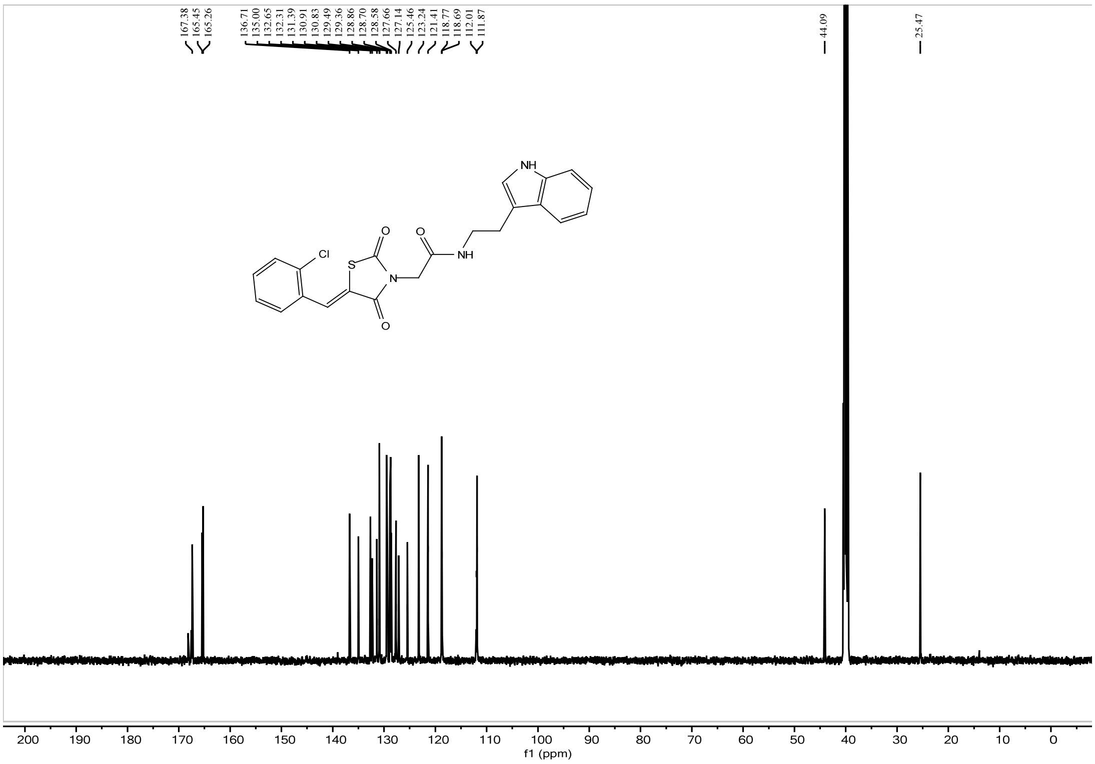

^{13}C NMR of compound **5h**

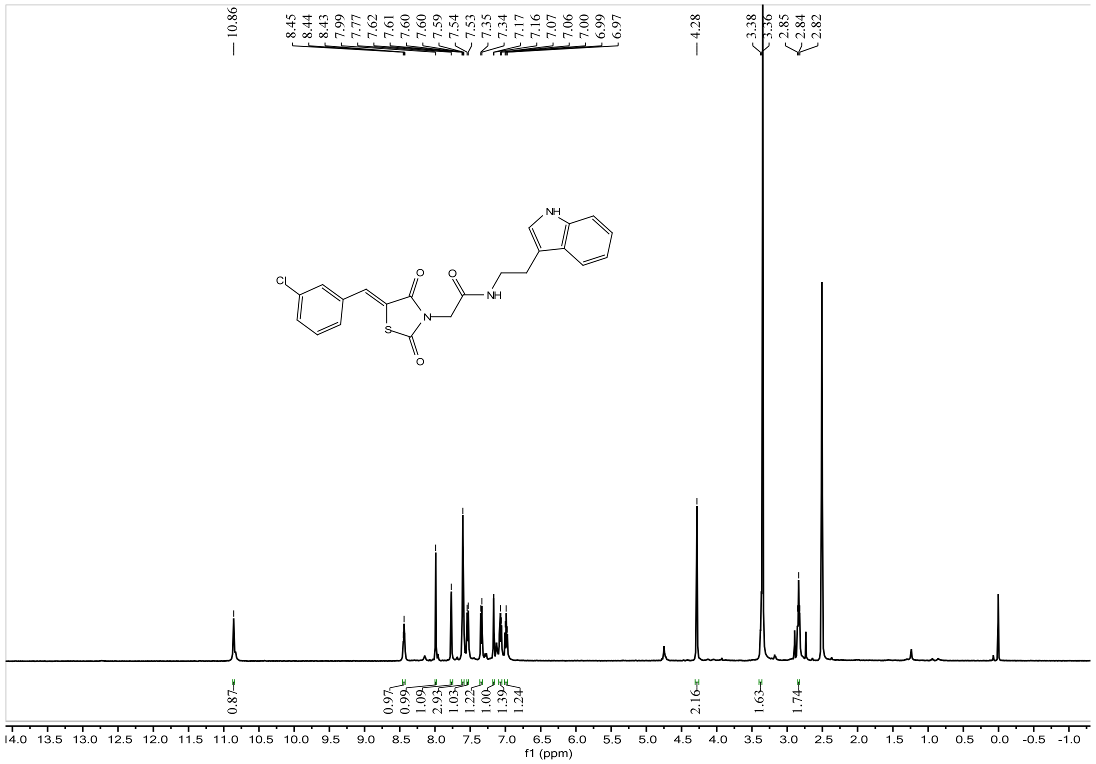

^{1}H NMR of compound **5i**

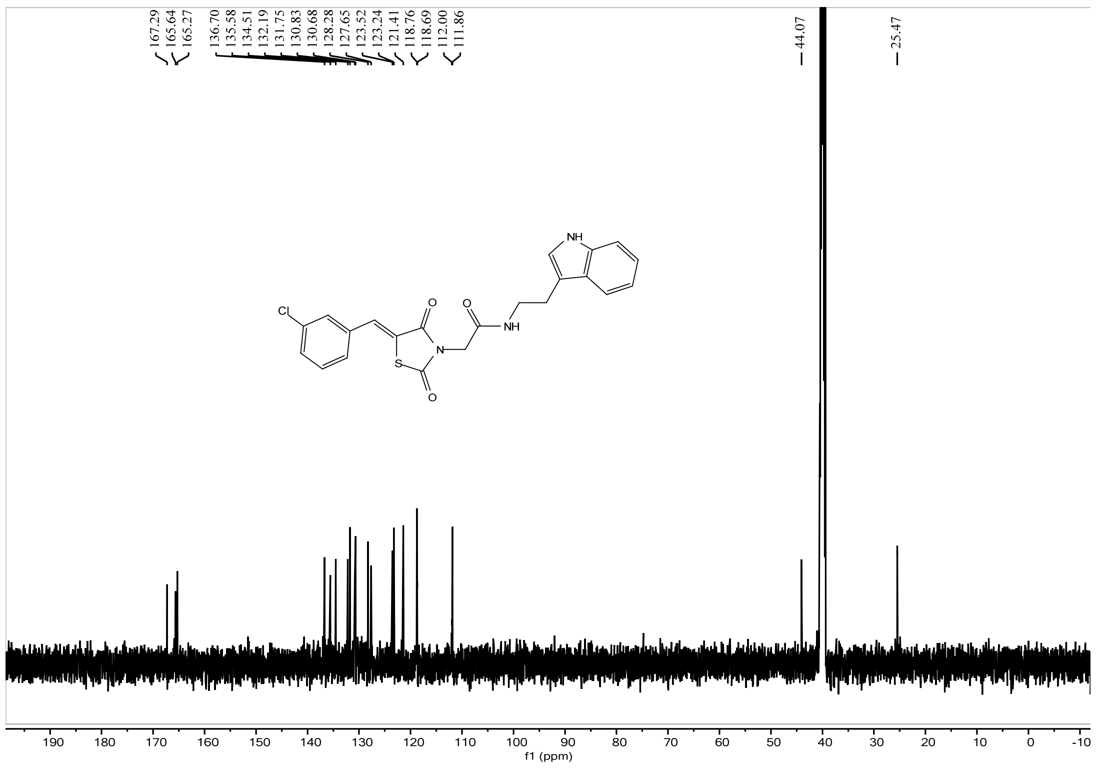

^{13}C NMR of compound **5i**

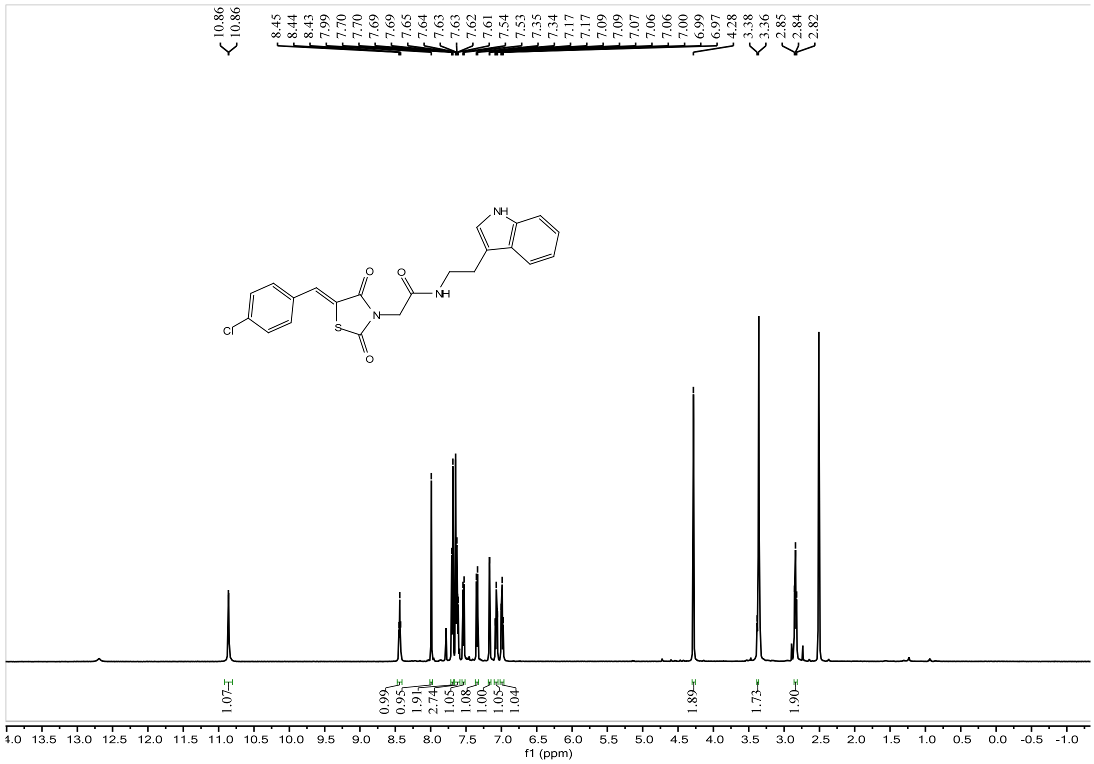

^{1}H NMR of compound **5j**

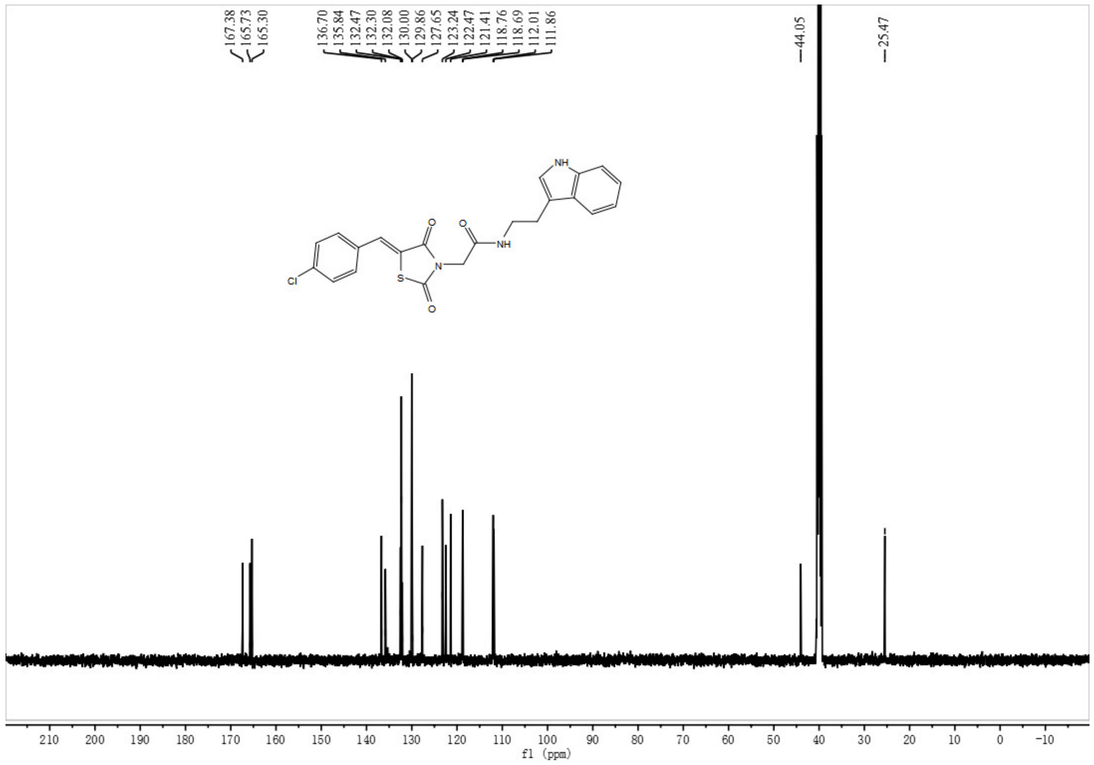

^{13}C NMR of compound **5j**

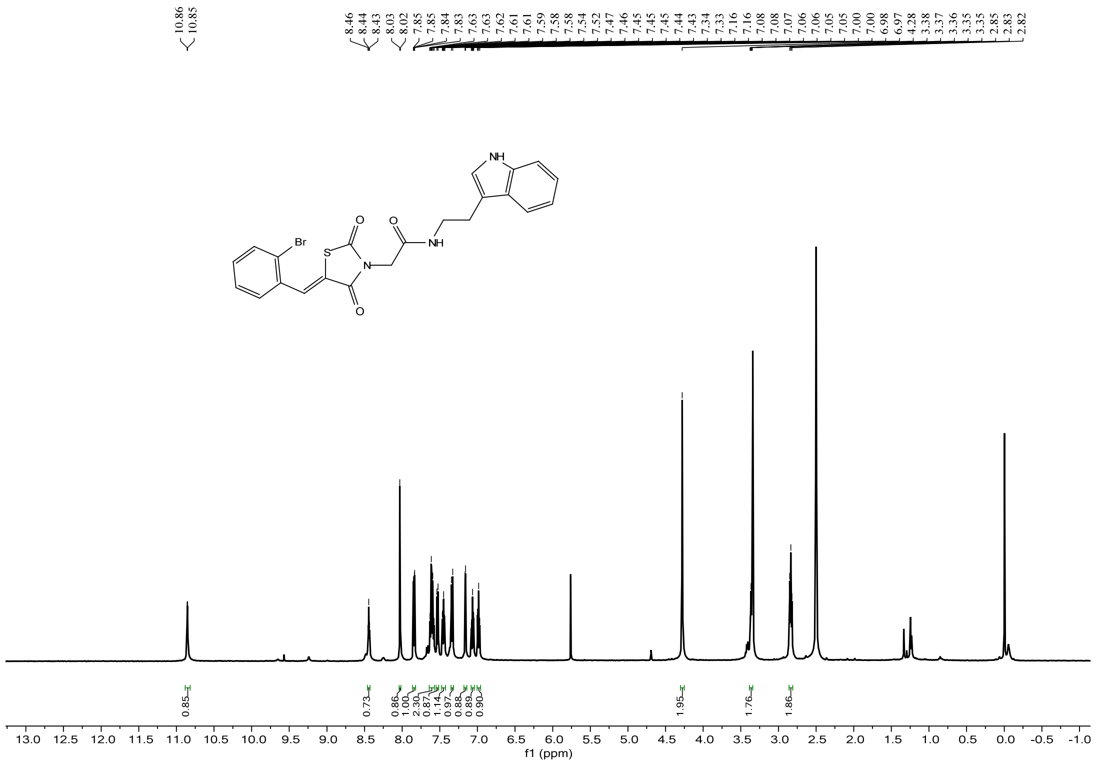

^{1}H NMR of compound **5k**

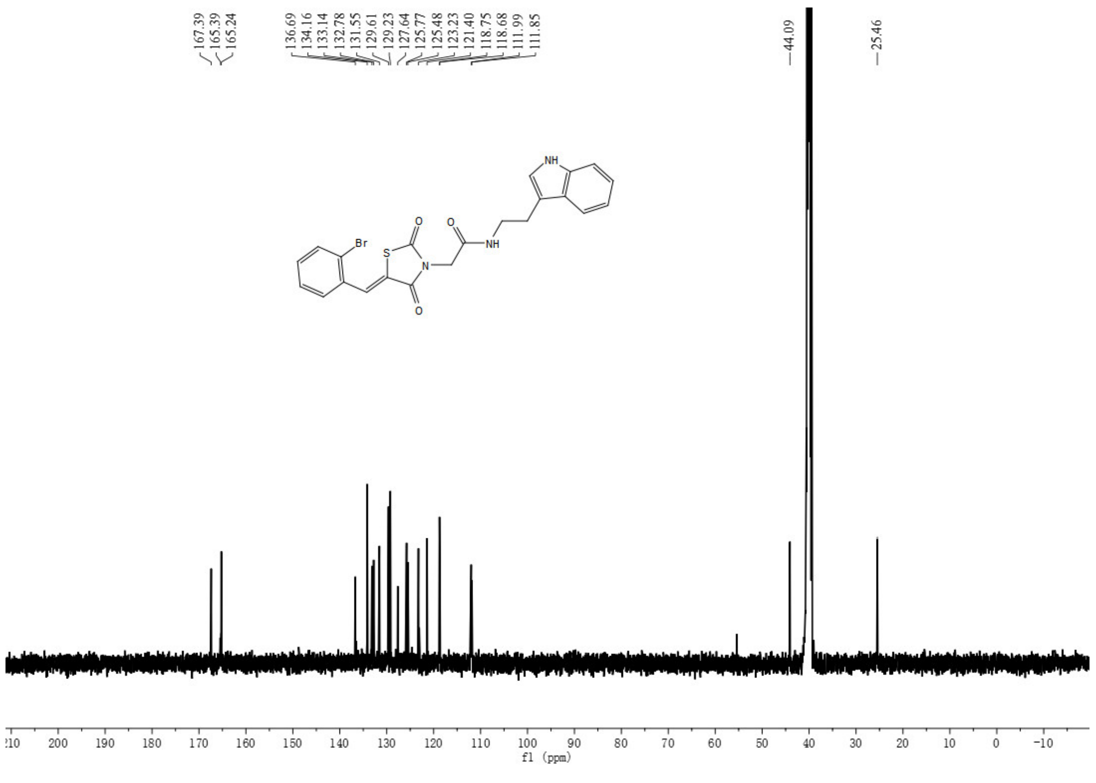

^{13}C NMR of compound **5k**

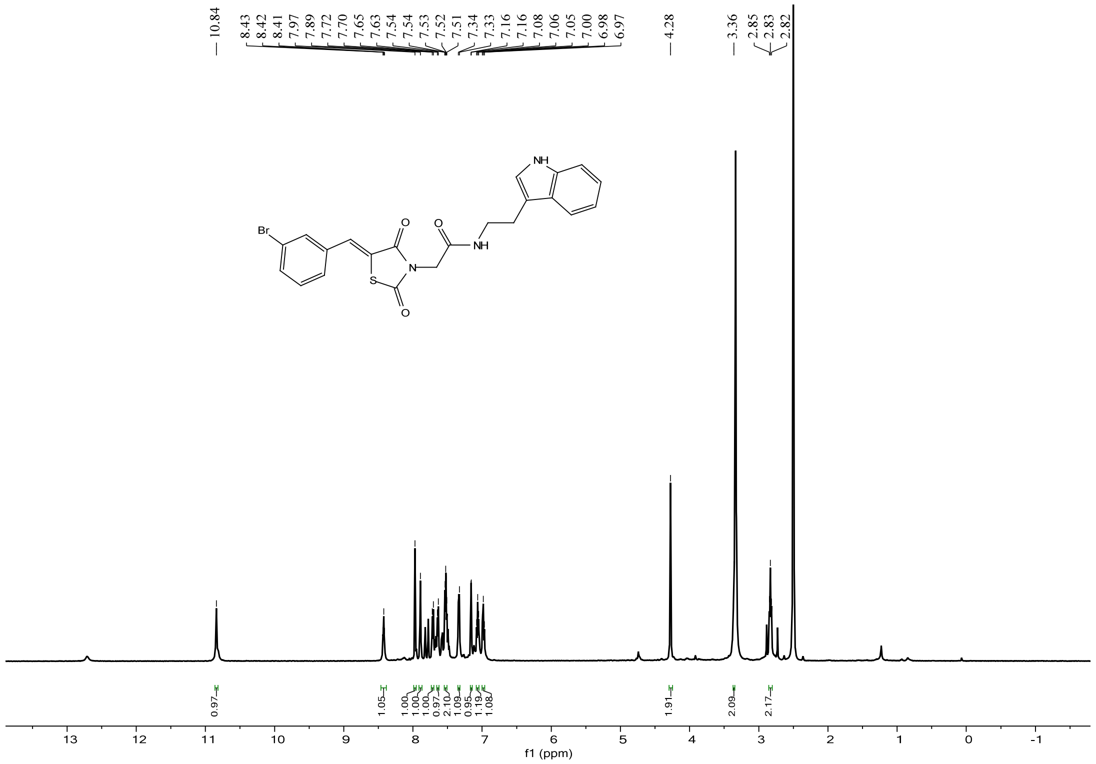

^{1}H NMR of compound **5l**

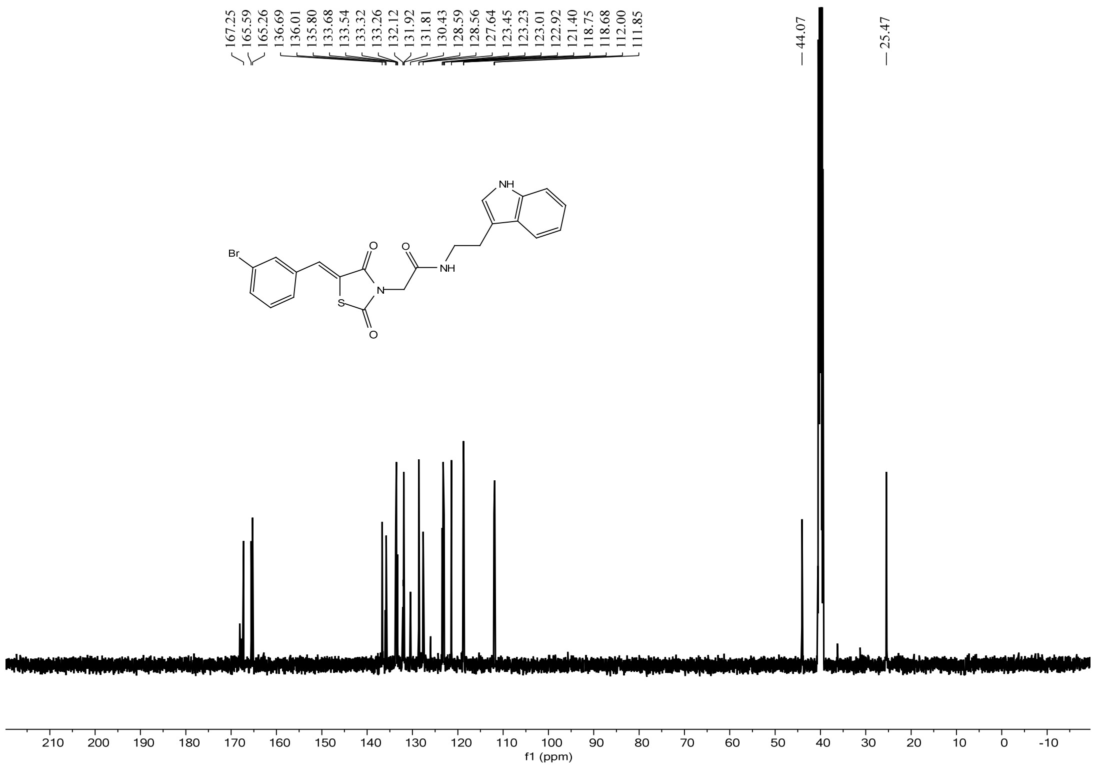

^{13}C NMR of compound **5l**

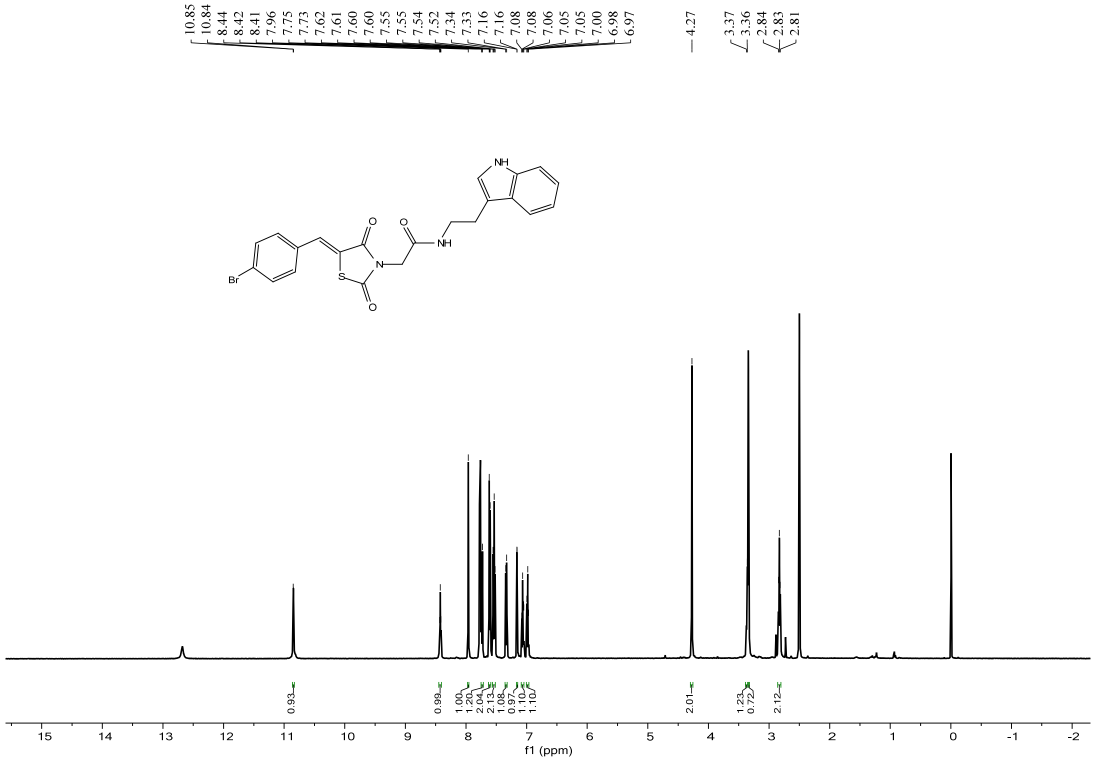

^{1}H NMR of compound **5m**

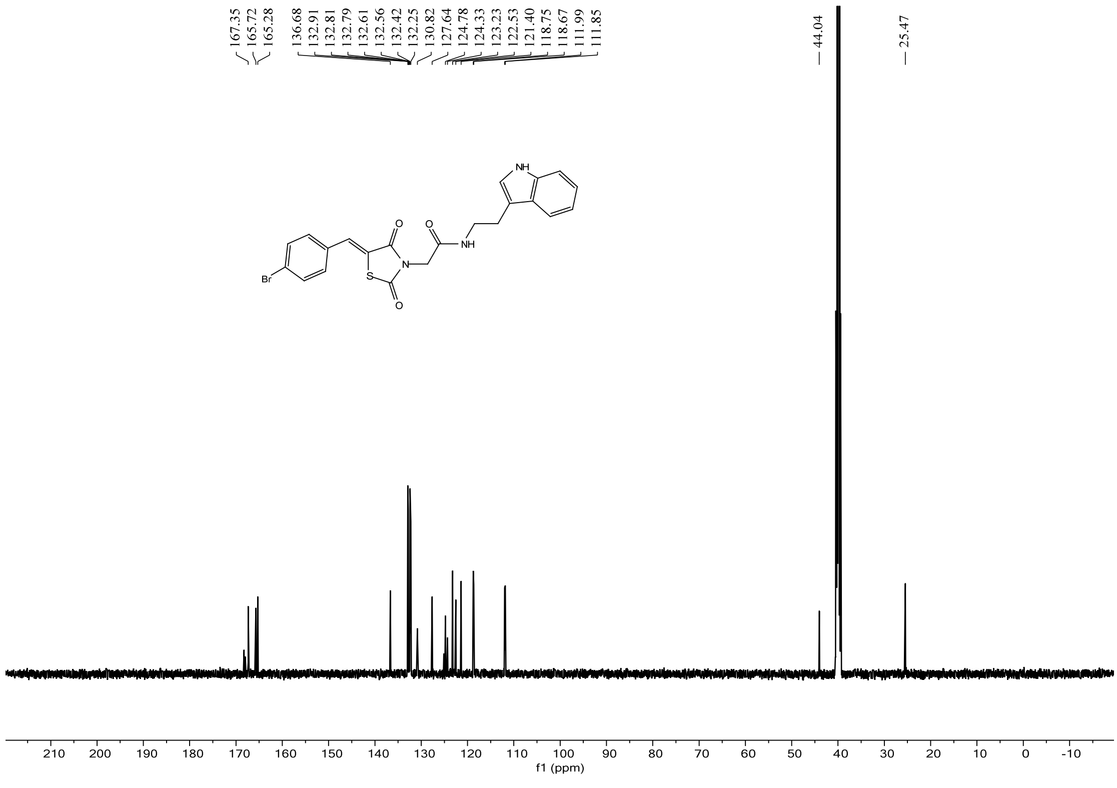

^{13}C NMR of compound **5m**

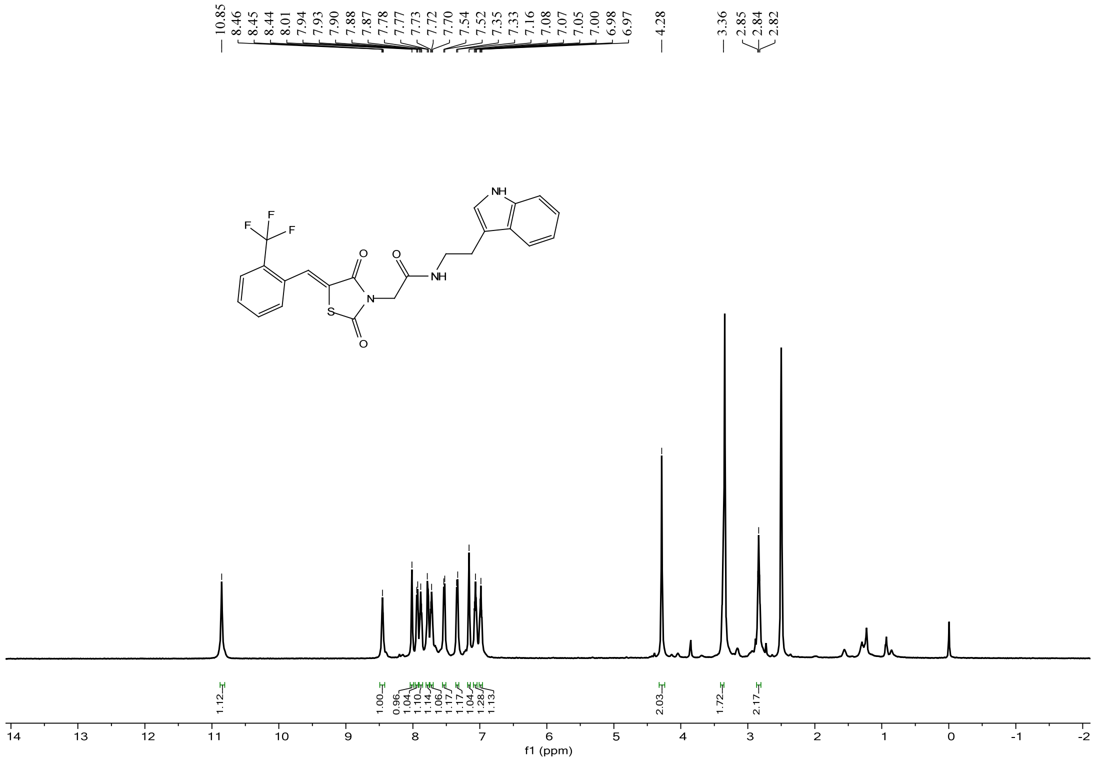

^{1}H NMR of compound **5n**

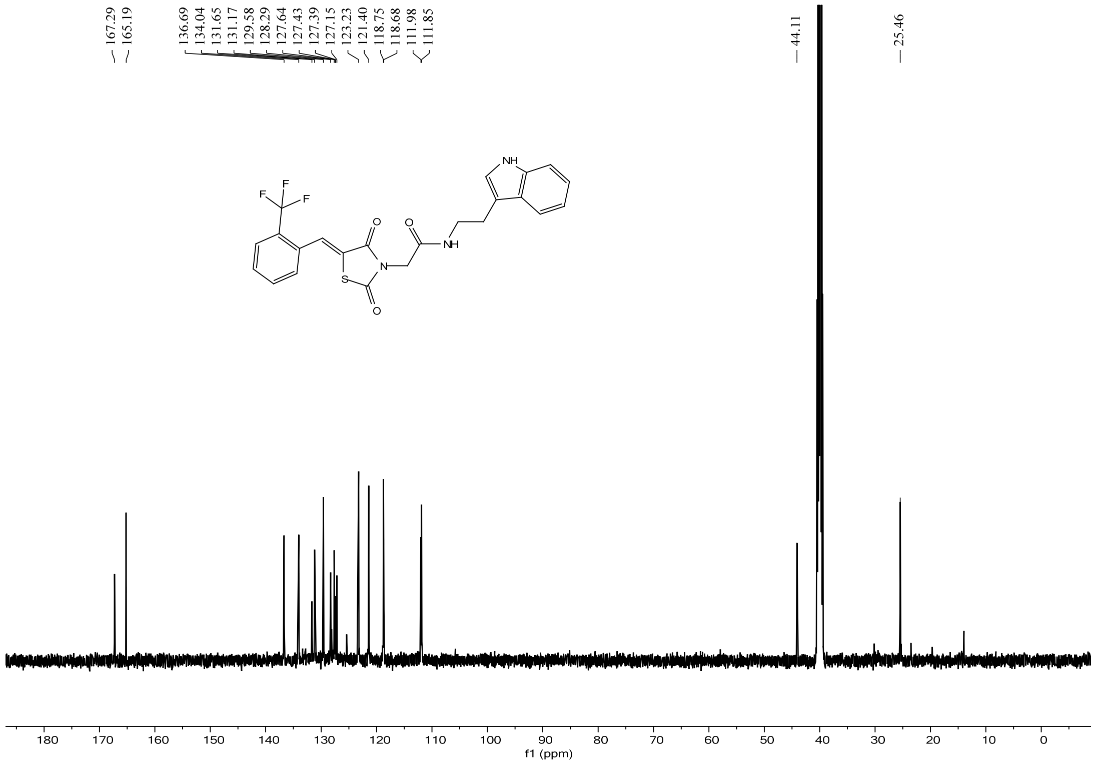

^{13}C NMR of compound **5n**

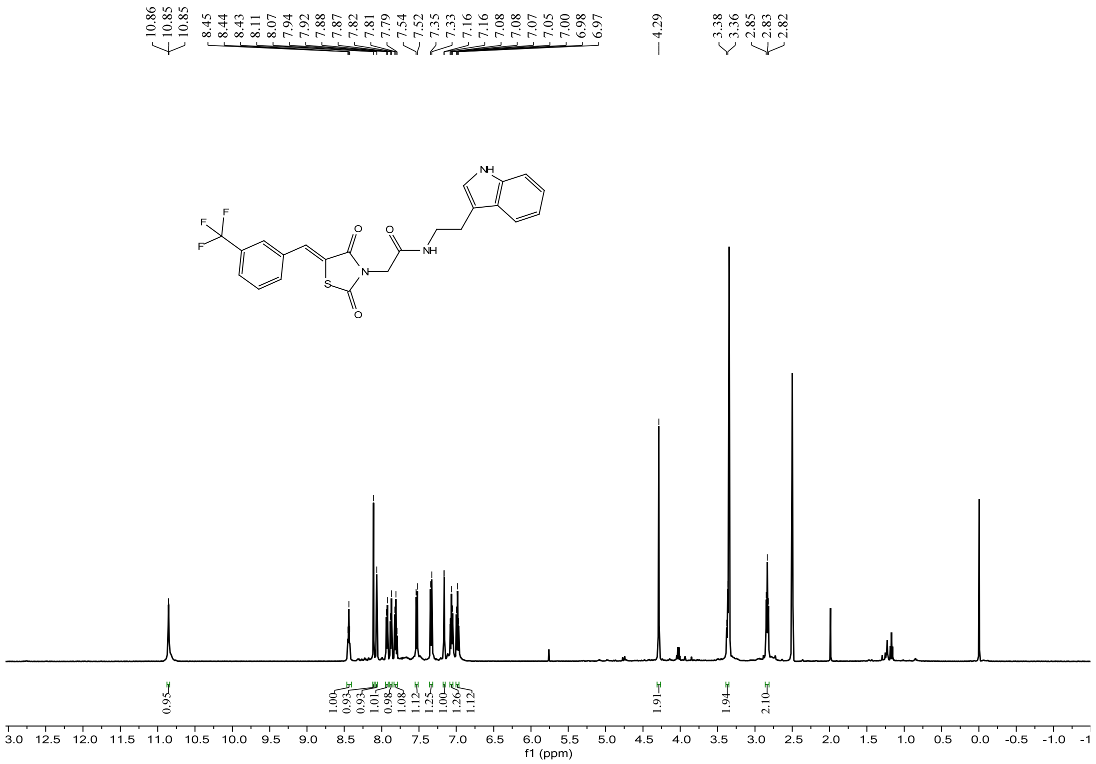

^{1}H NMR of compound **5o**

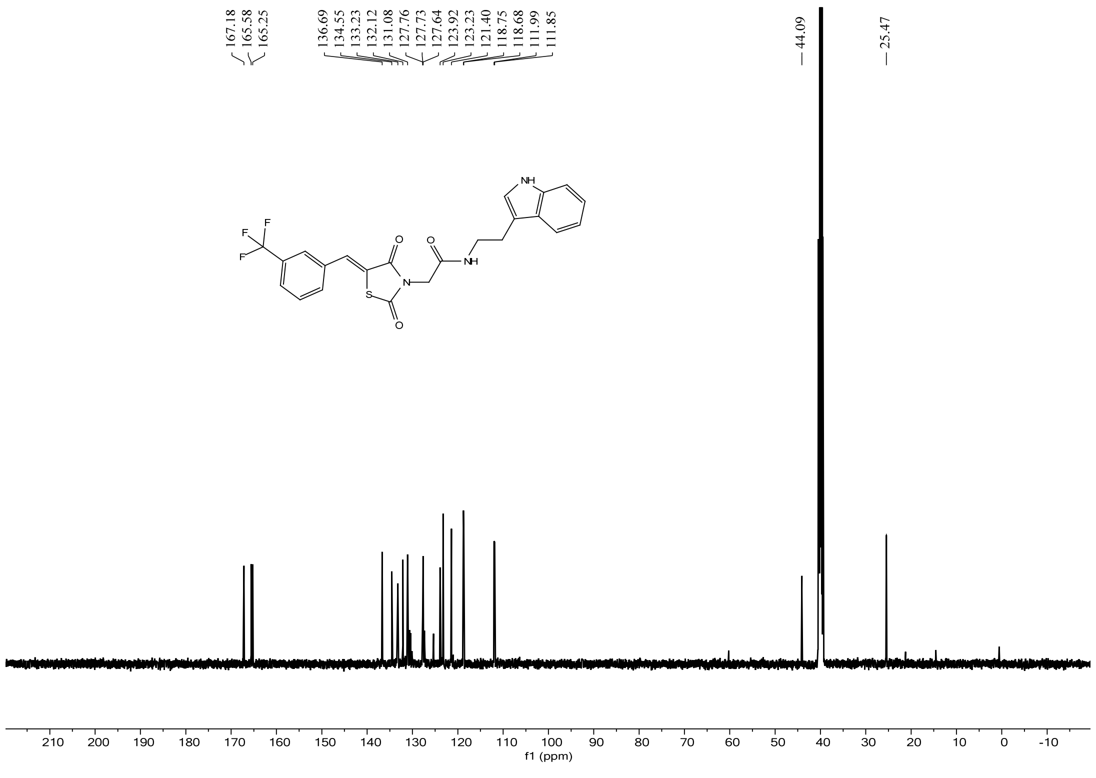

^{13}C NMR of compound **5o**

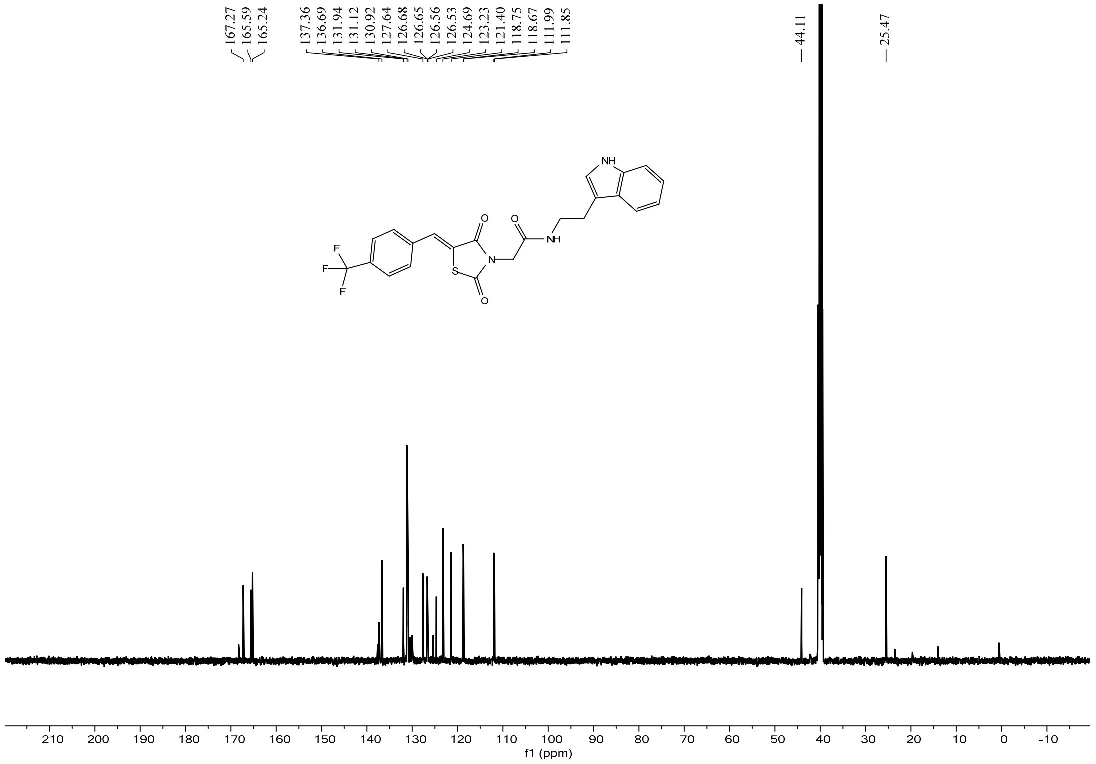

^{1}H NMR of compound **5p**

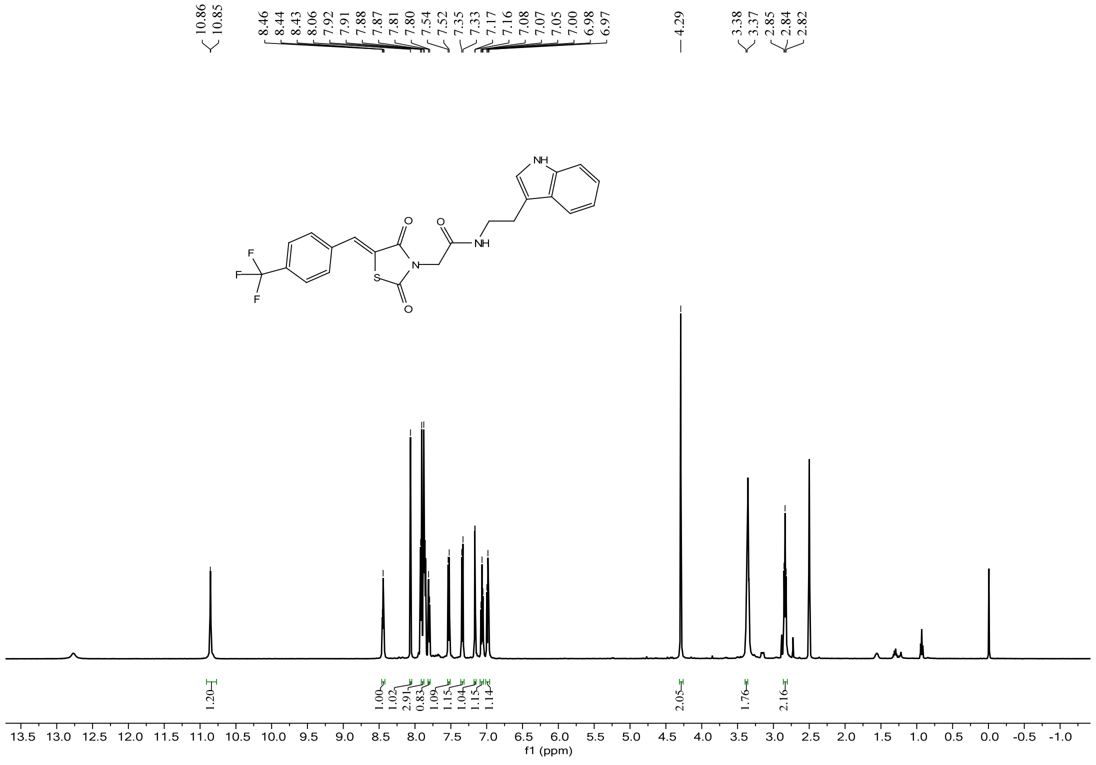

^{13}C NMR of compound **5p**

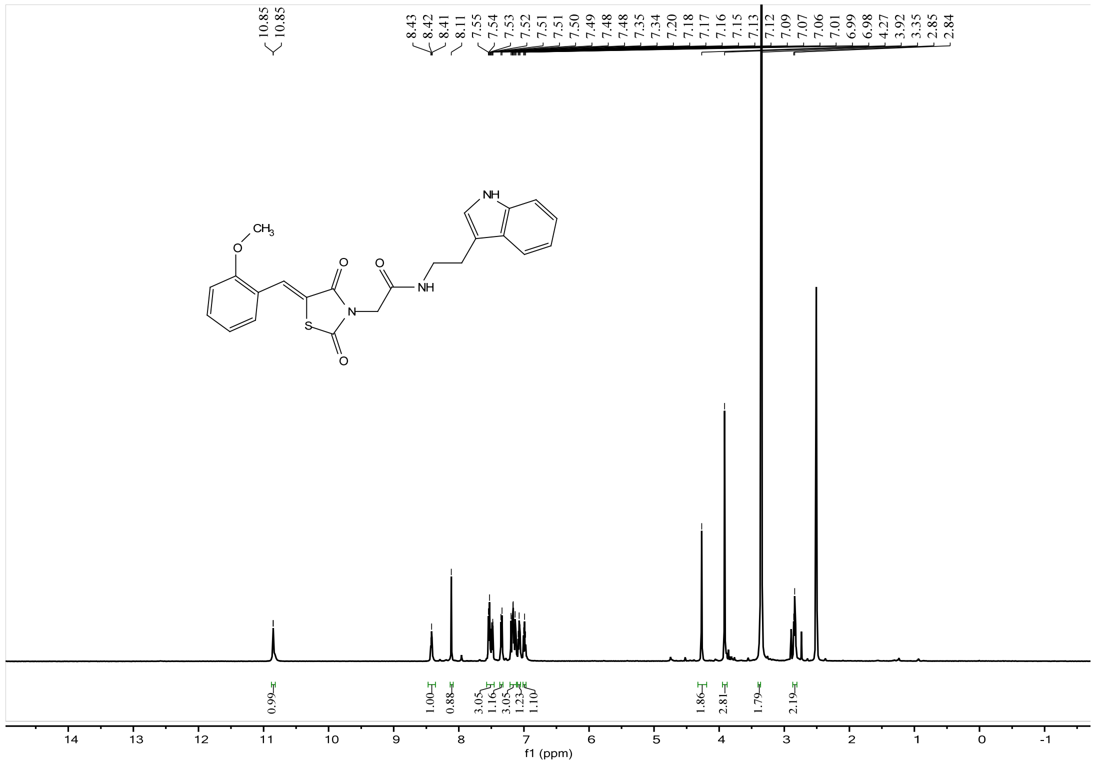

^{1}H NMR of compound **5q**

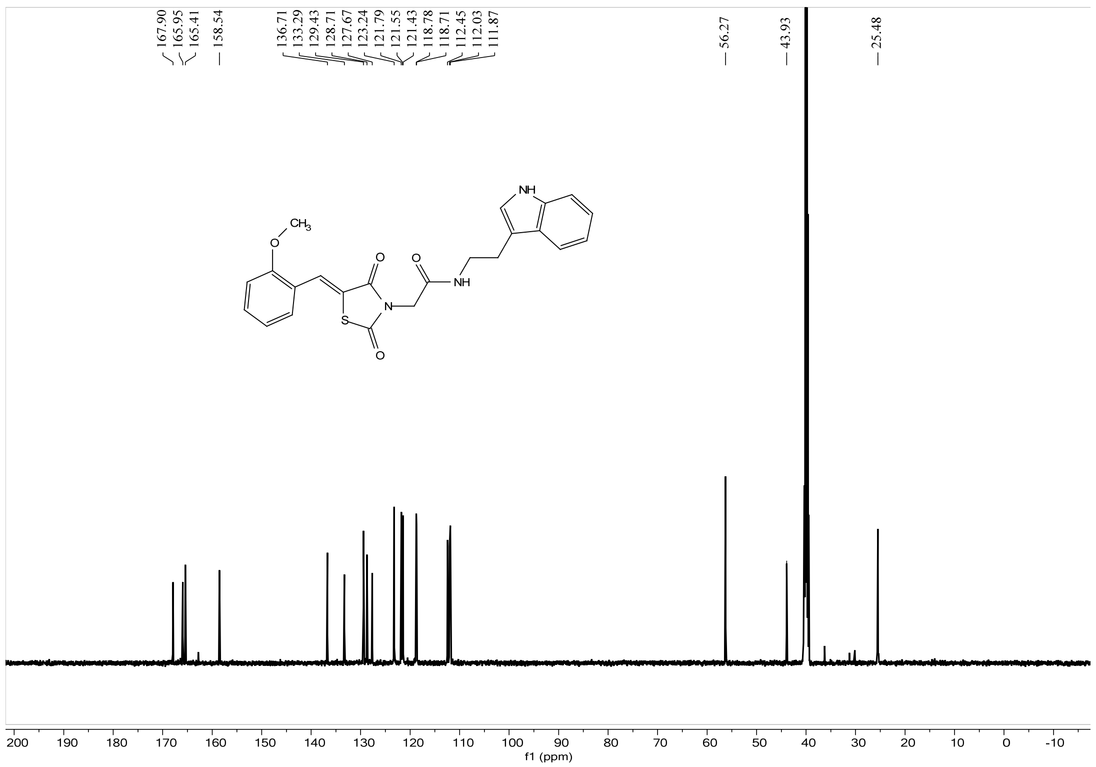

^{13}C NMR of compound **5q**

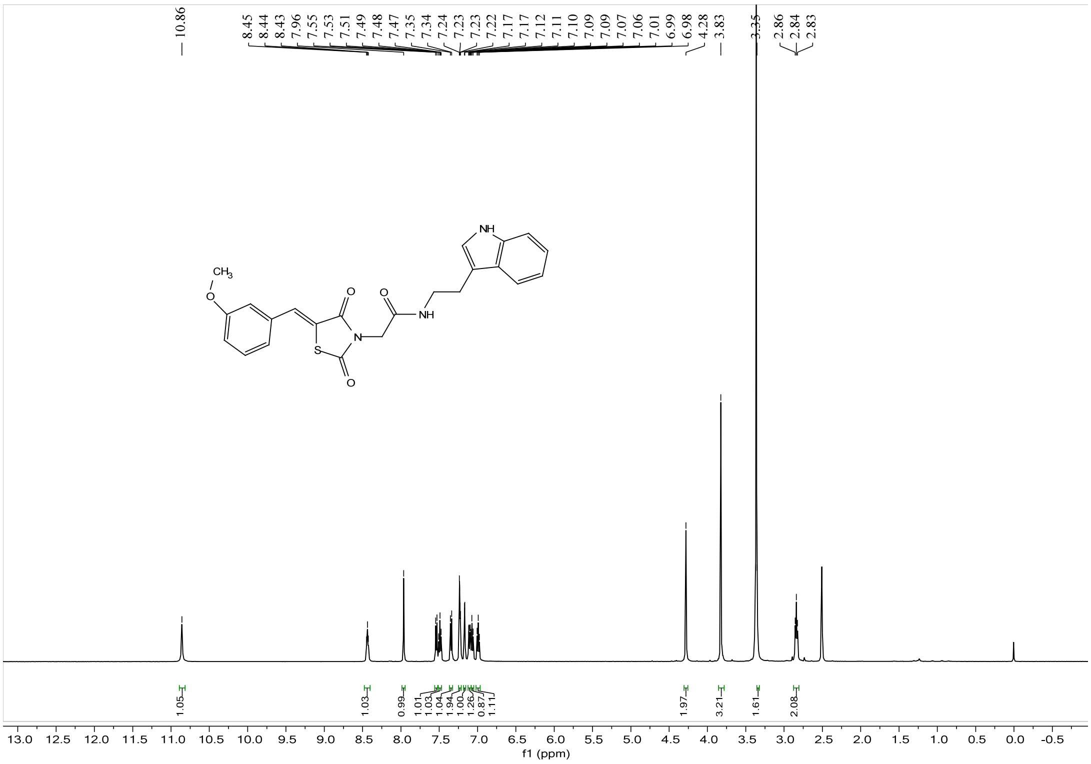

^{1}H NMR of compound **5r**

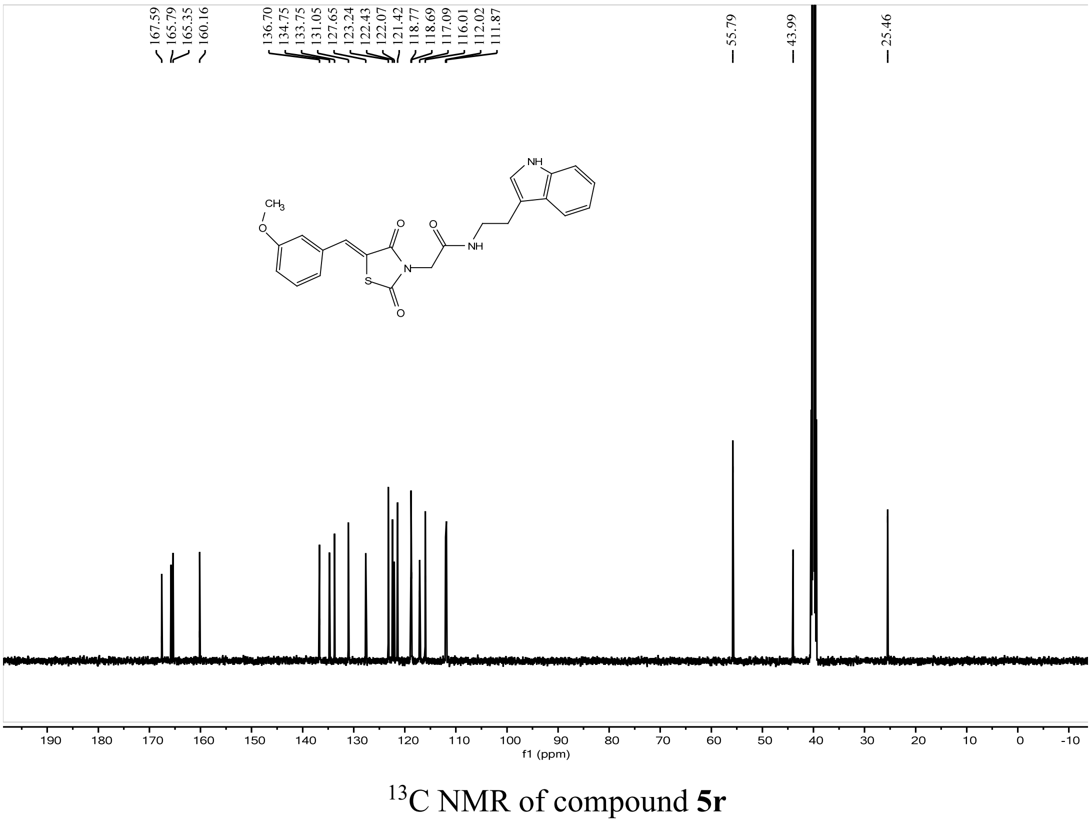

^{13}C NMR of compound **5r**

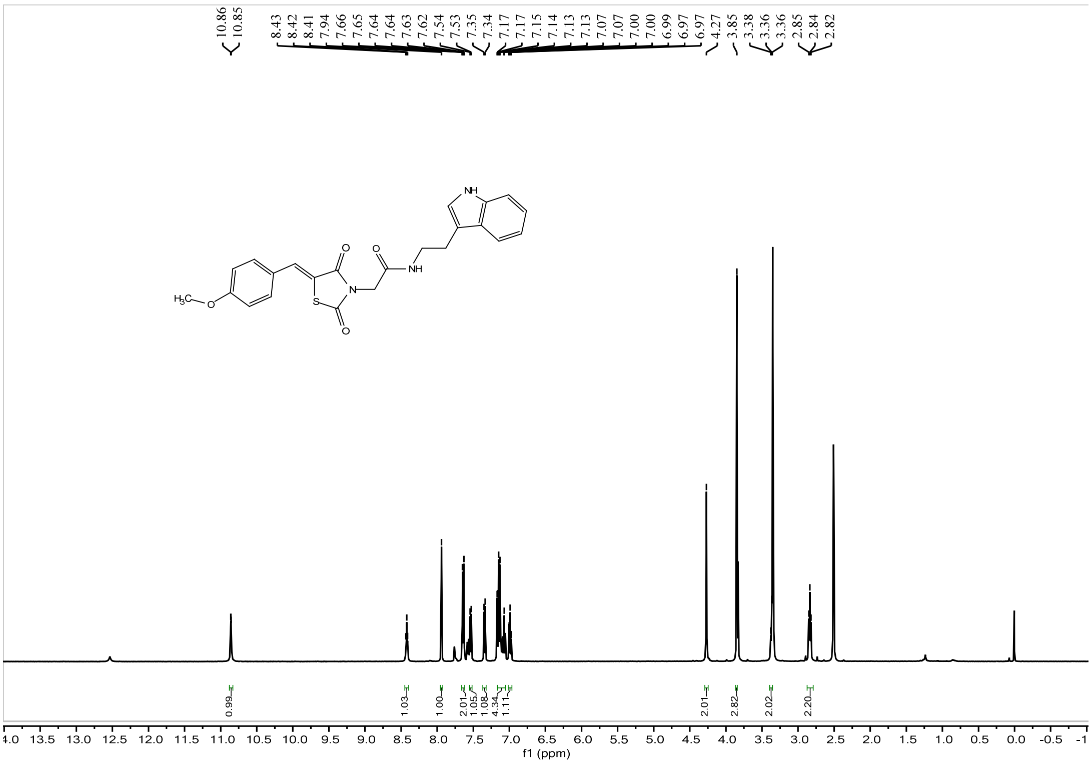

^{1}H NMR of compound **5s**

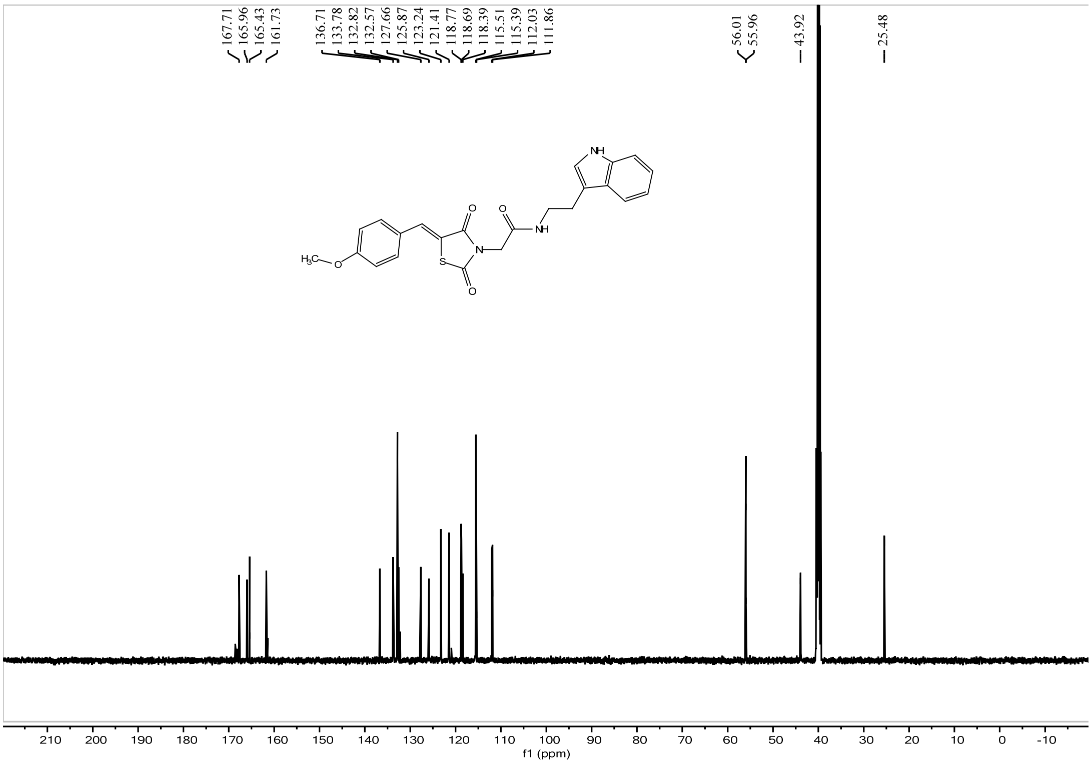

^{13}C NMR of compound **5s**

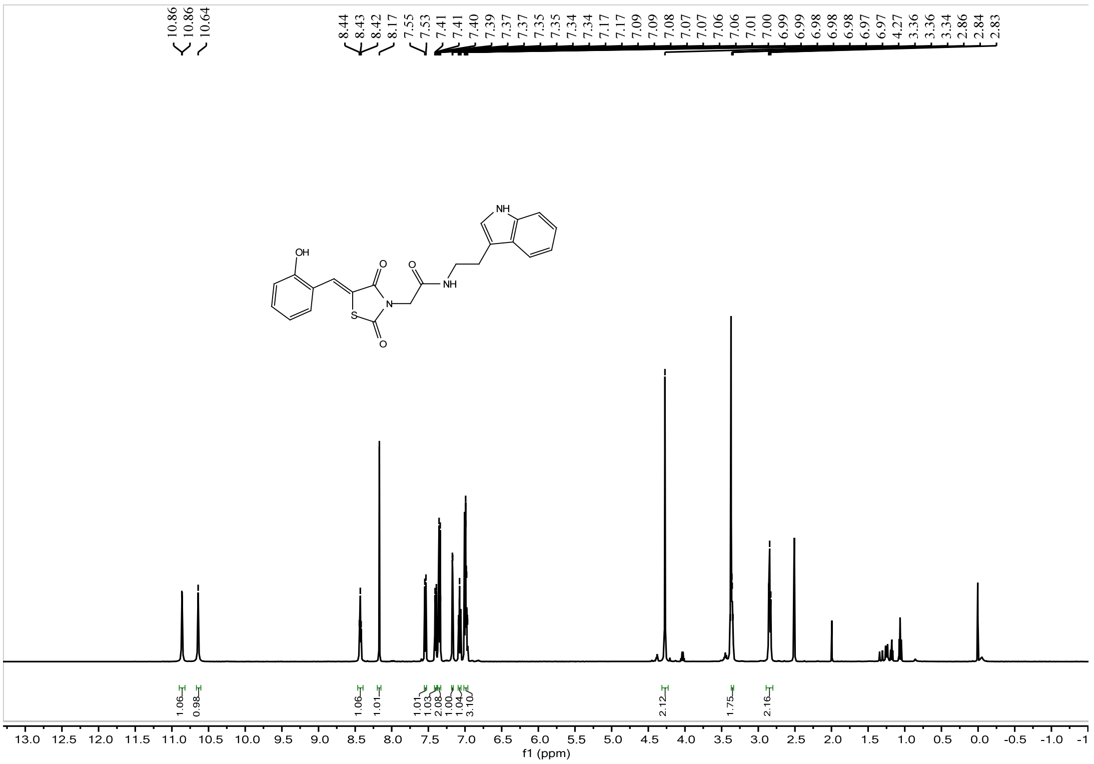

^{1}H NMR of compound **5t**

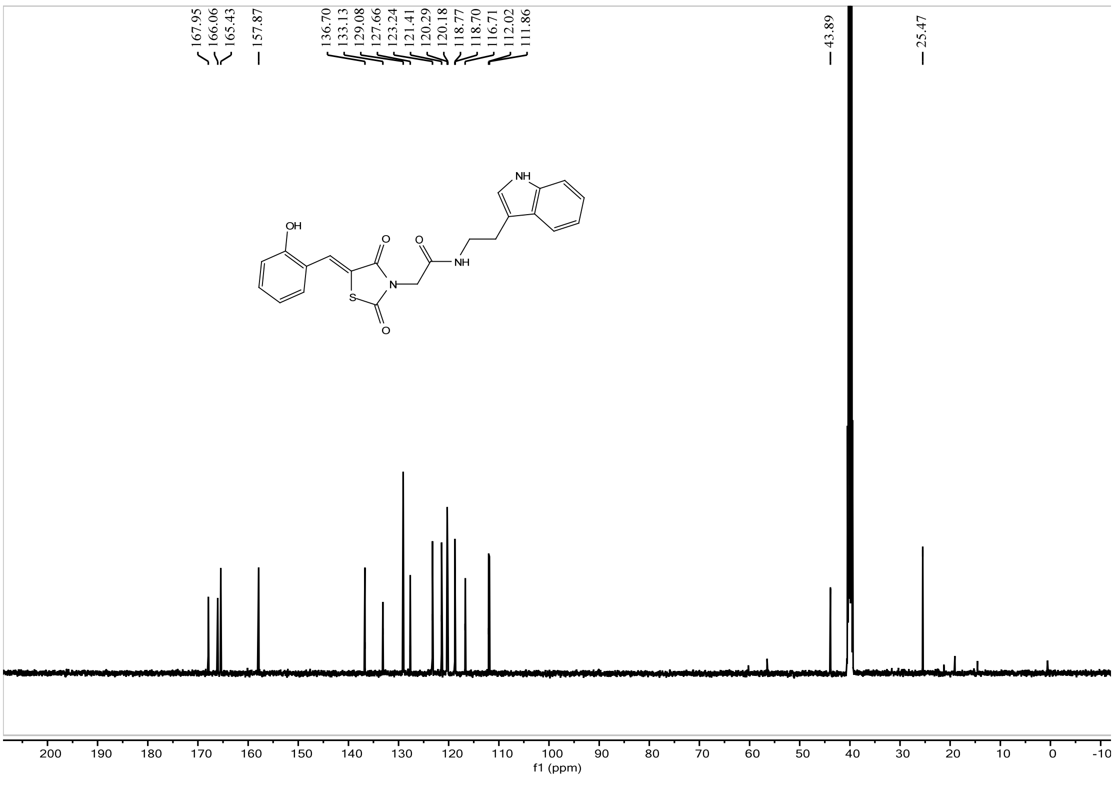

^{13}C NMR of compound **5t**

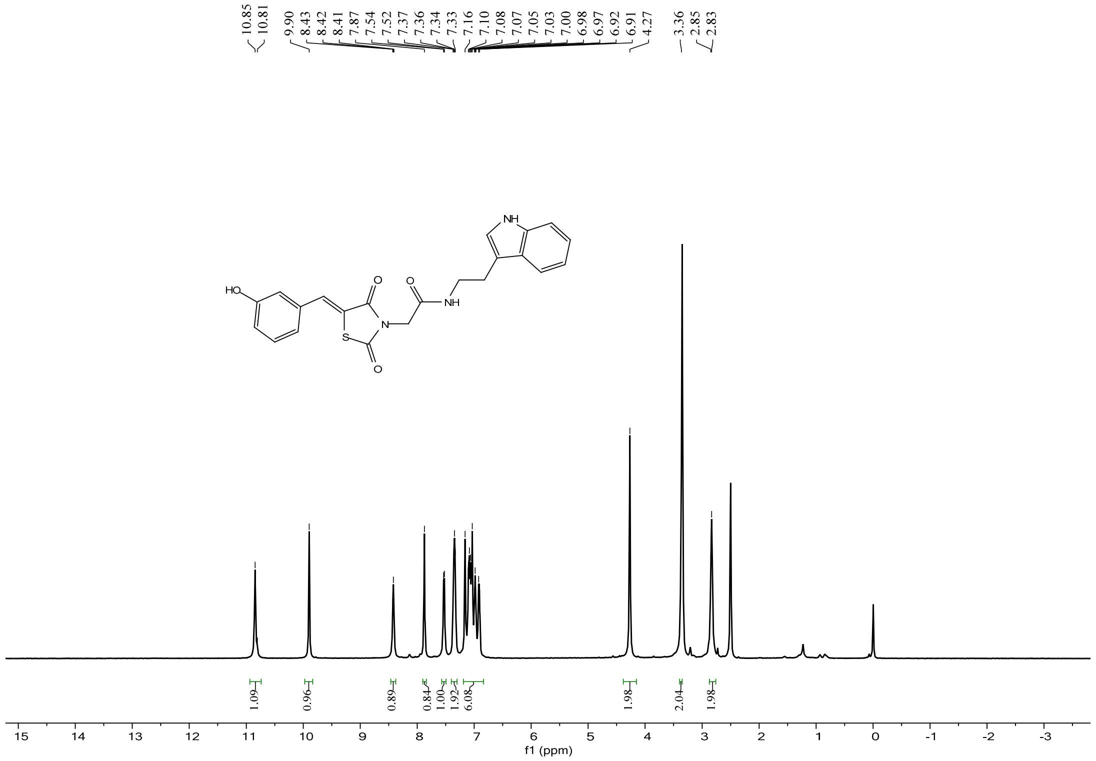

^{1}H NMR of compound **5u**

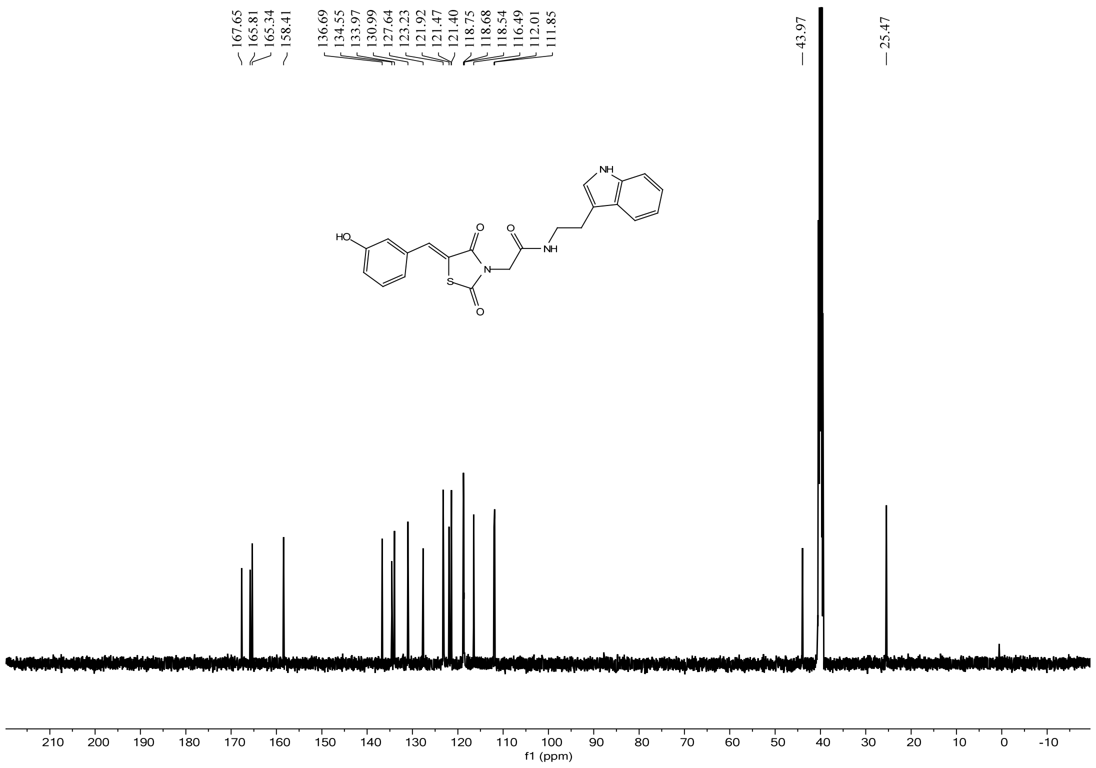

^{13}C NMR of compound **5u**

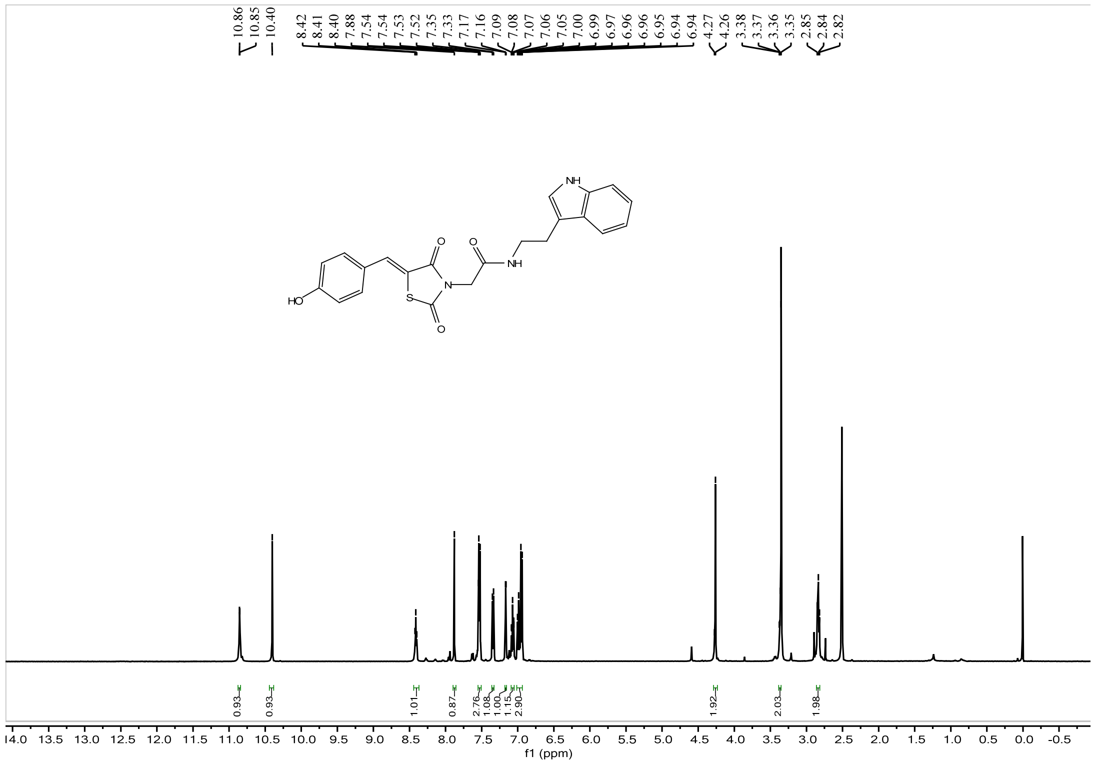

^{1}H NMR of compound **5v**

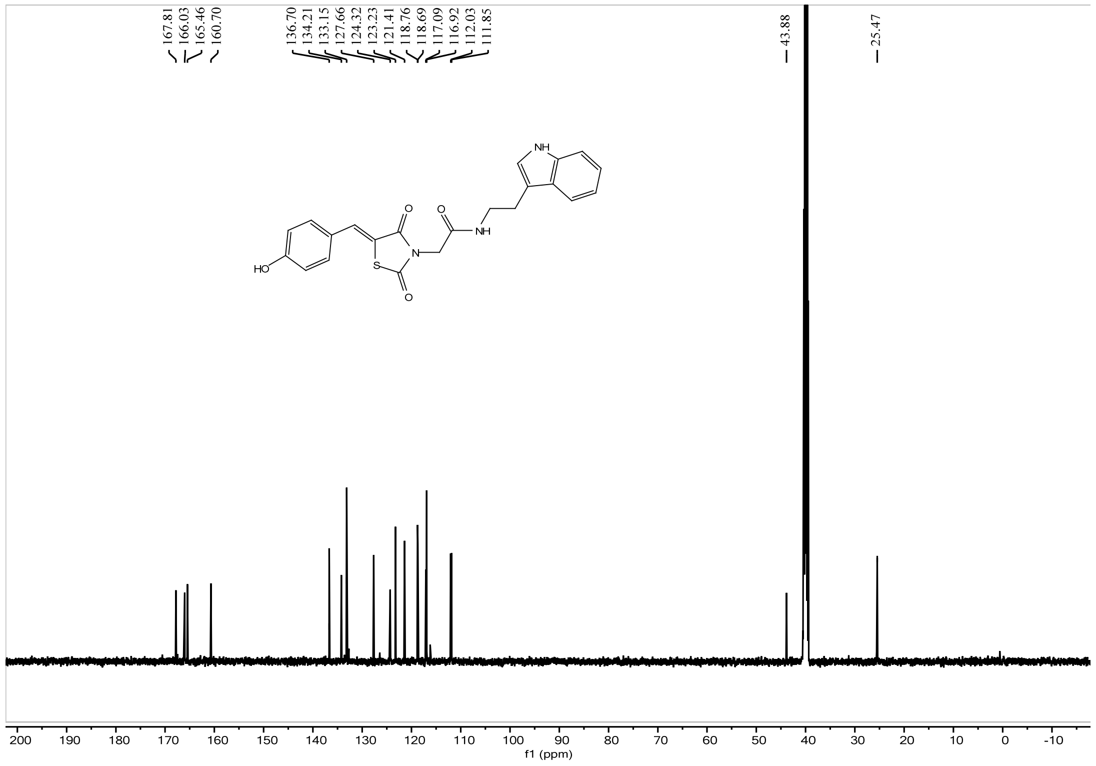

^{13}C NMR of compound **5v**

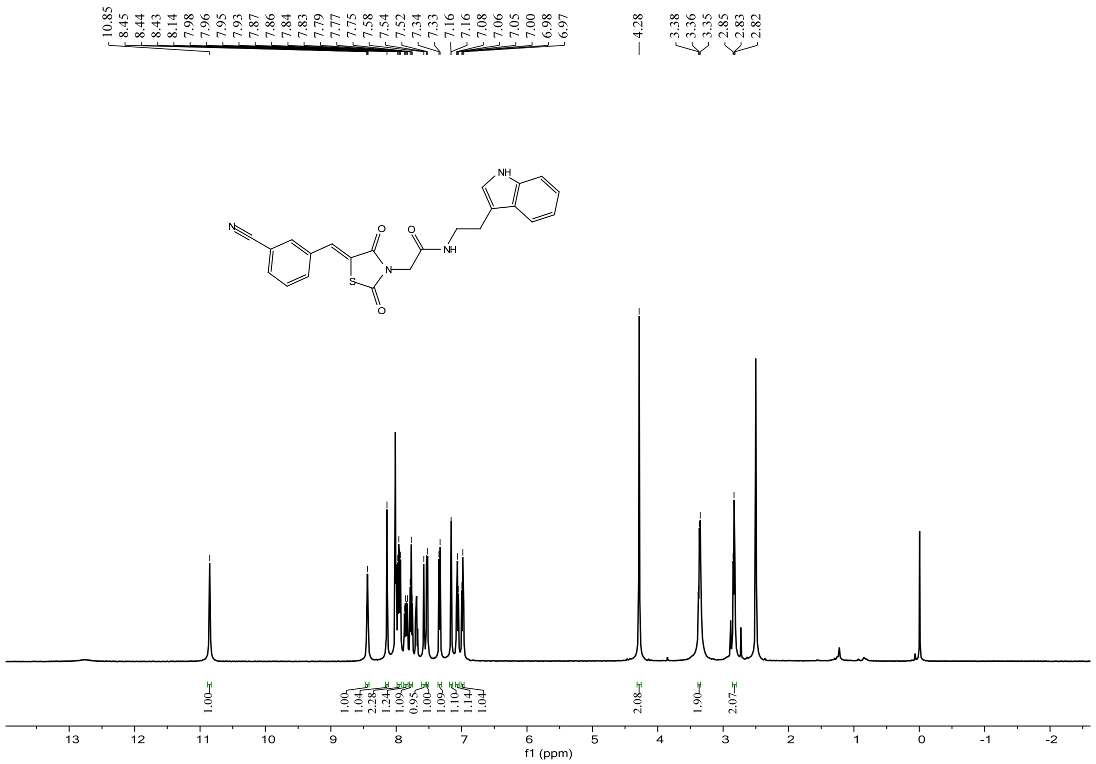

^{1}H NMR of compound **5w**

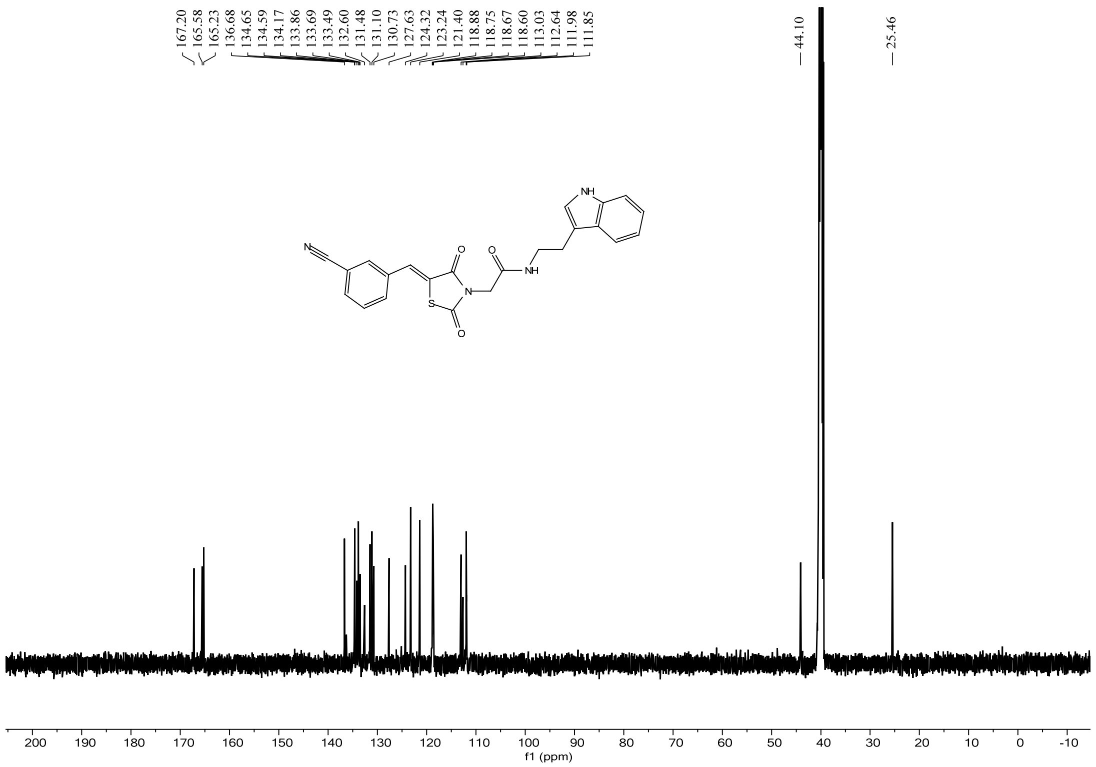

^{13}C NMR of compound **5w**

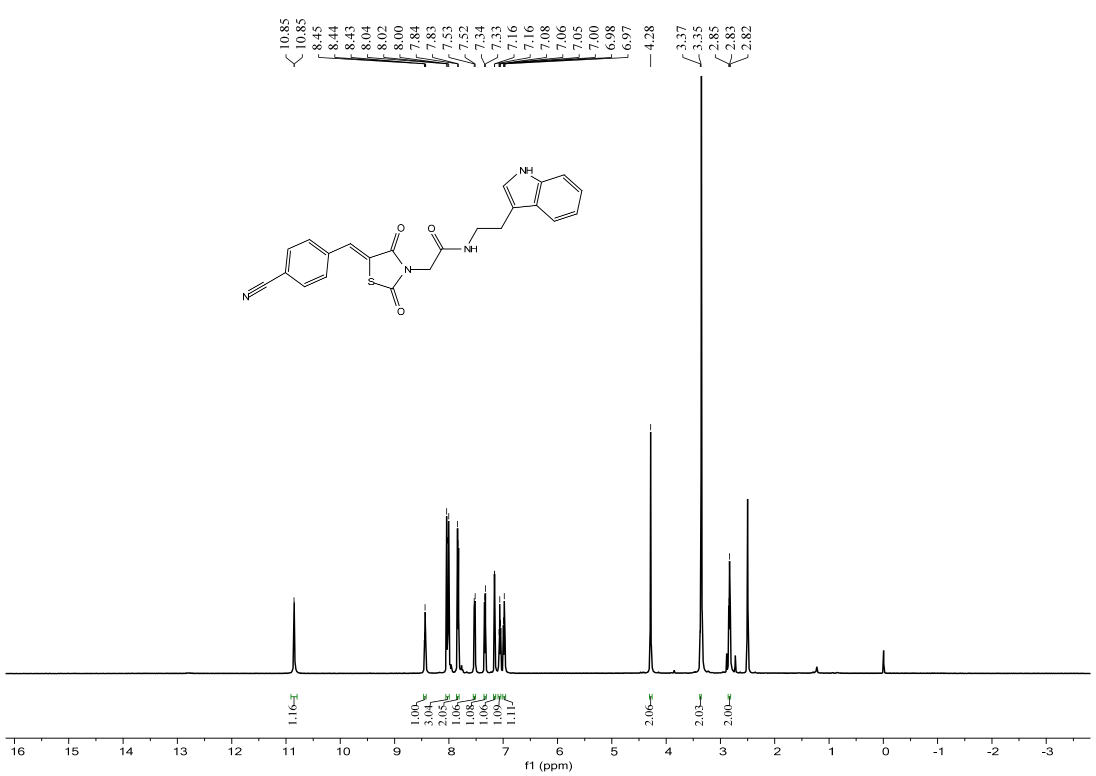

^{1}H NMR of compound **5x**

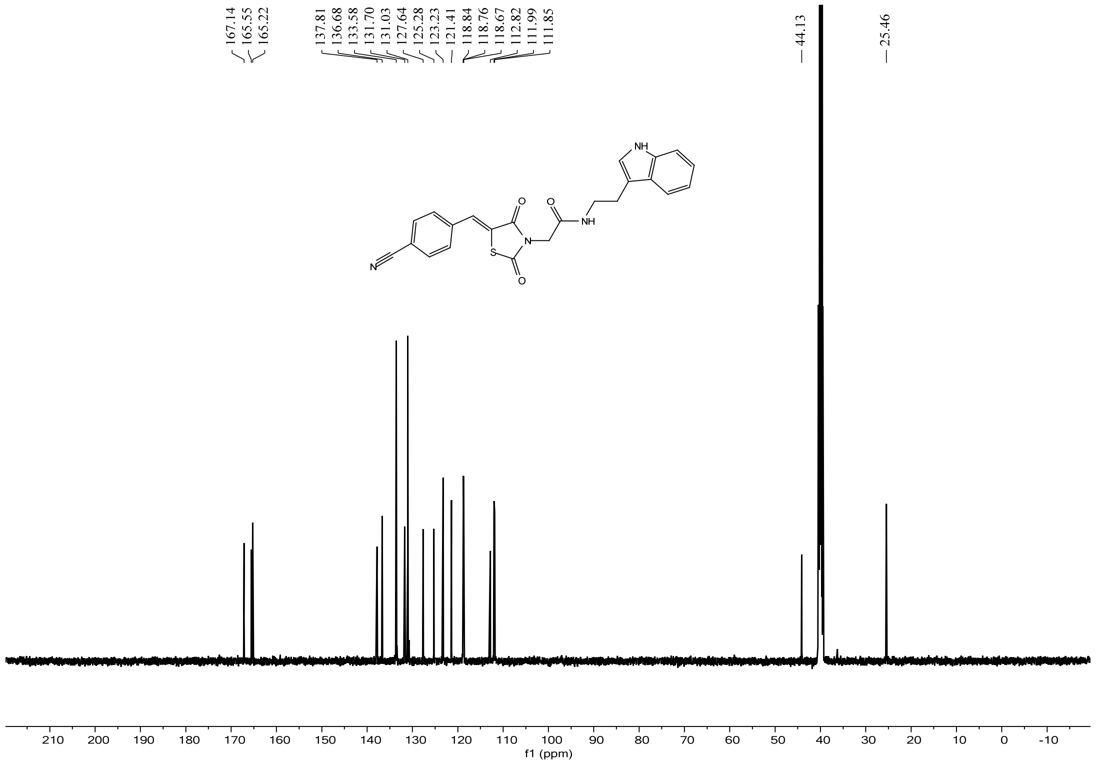

^{13}C NMR of compound **5x**

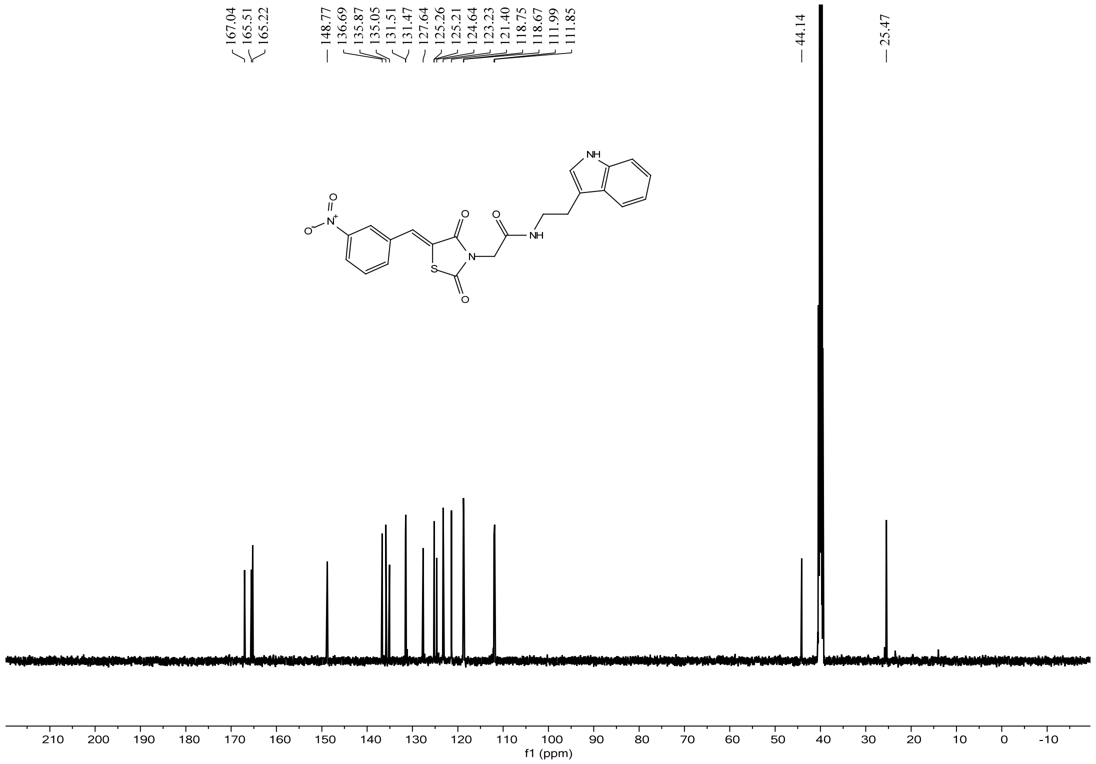

^{1}H NMR of compound **5y**

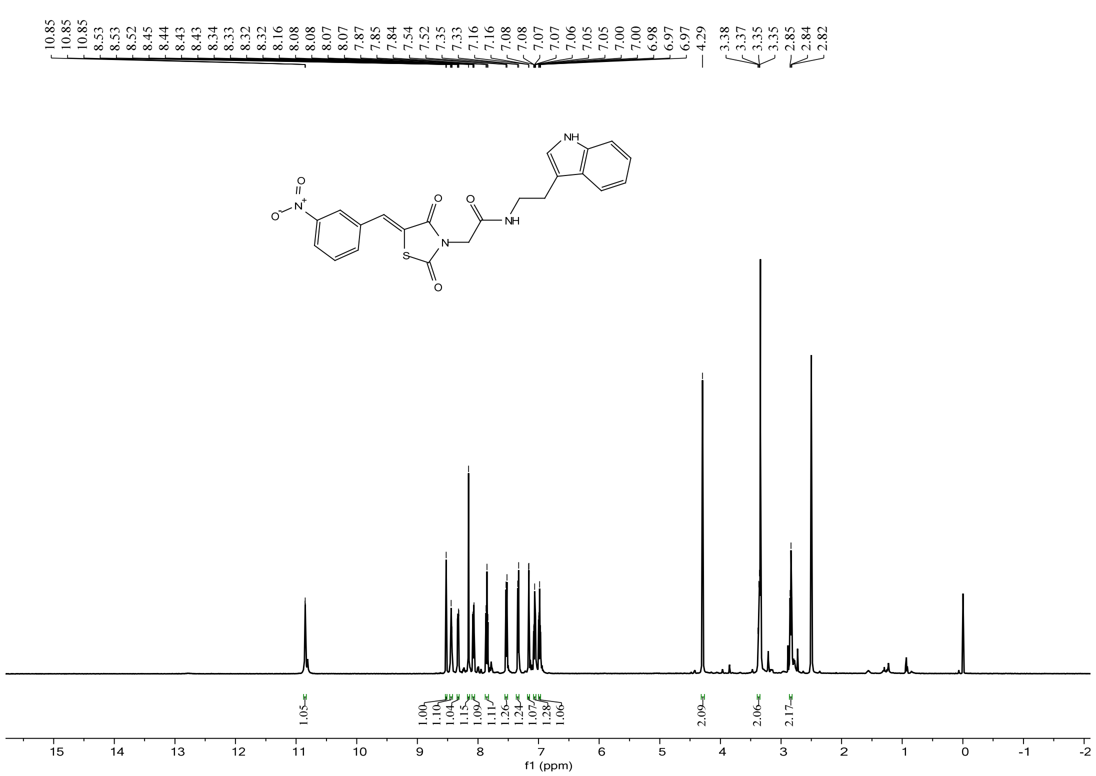

^{13}C NMR of compound **5y**

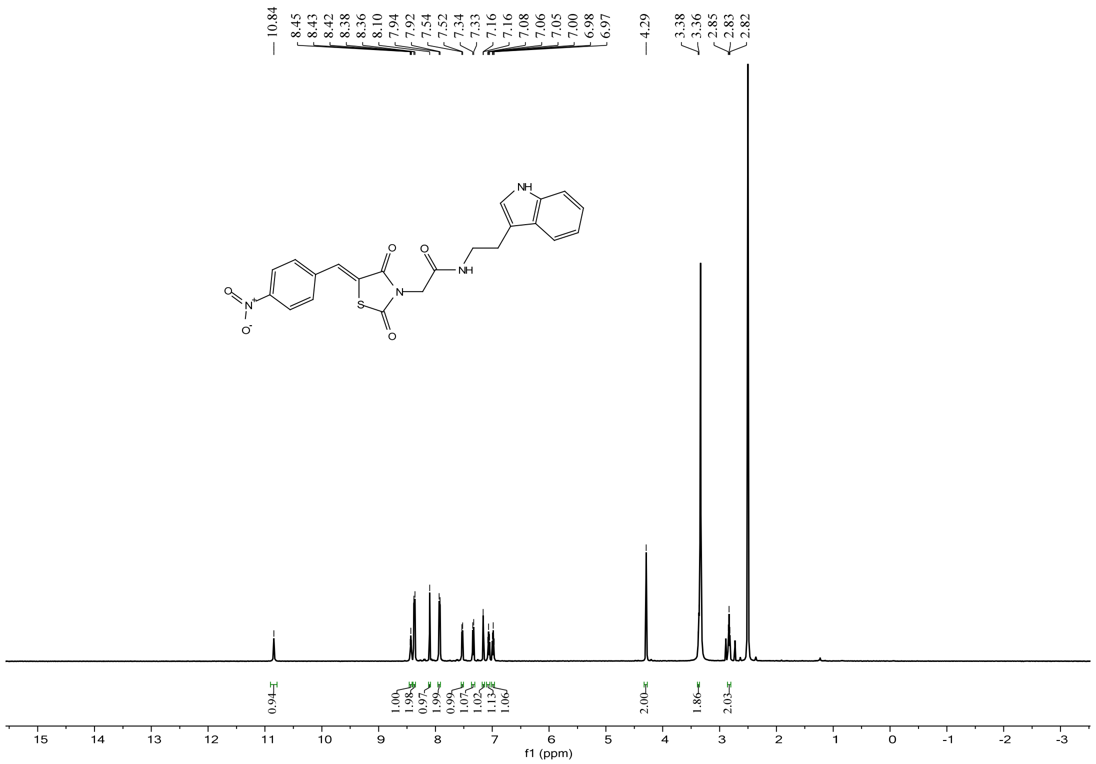

^{1}H NMR of compound **5z**

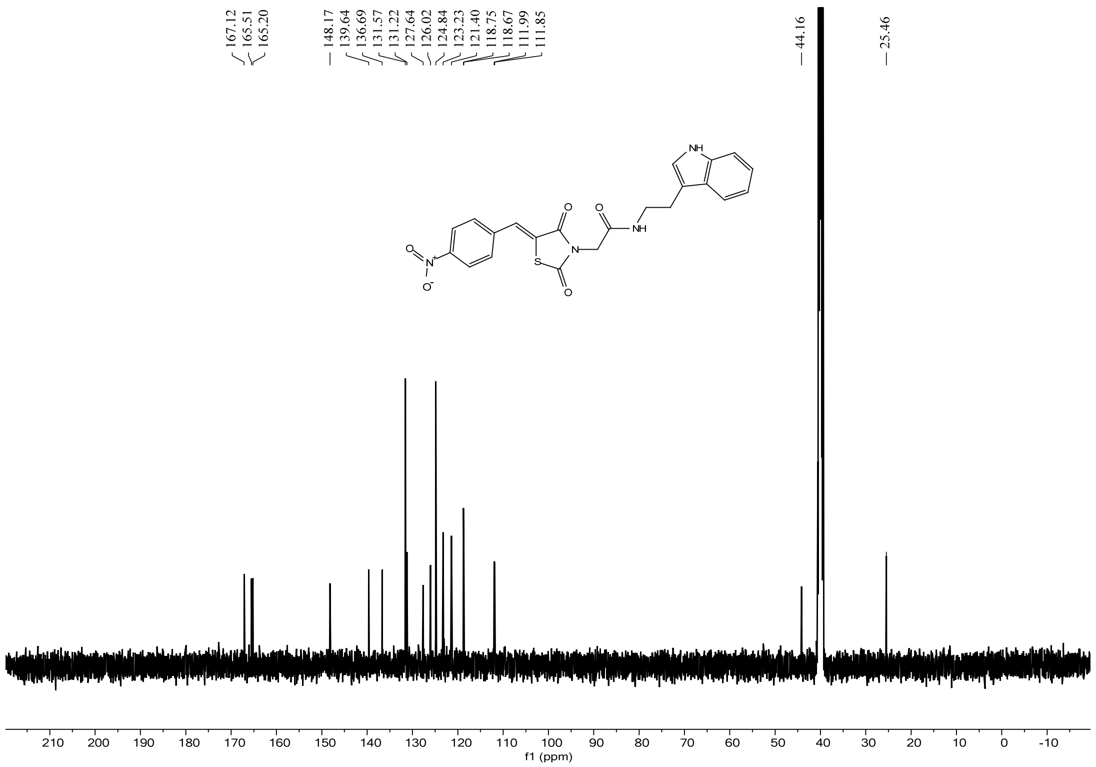

^{13}C NMR of compound **5z**

3. HRMS of compounds ***5a~5z***

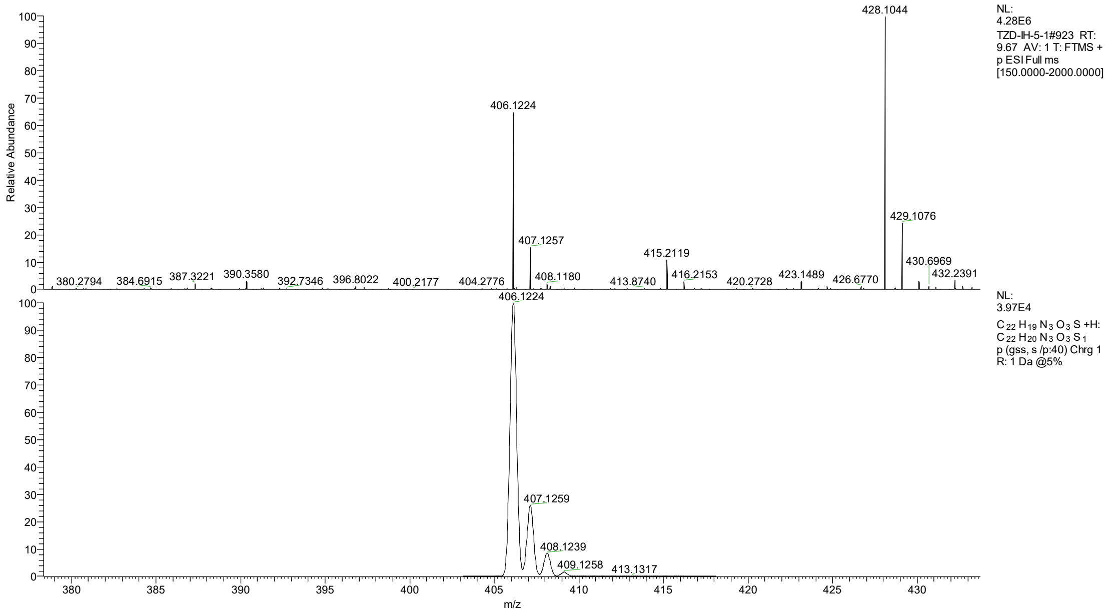

HRMS of compounds 5a

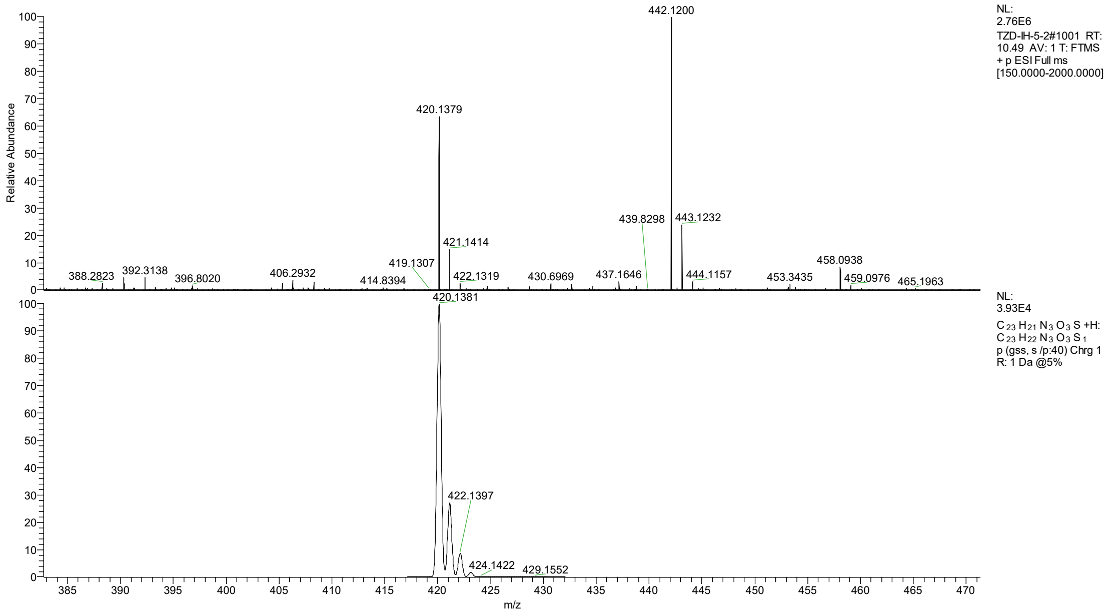

HRMS of compounds 5b

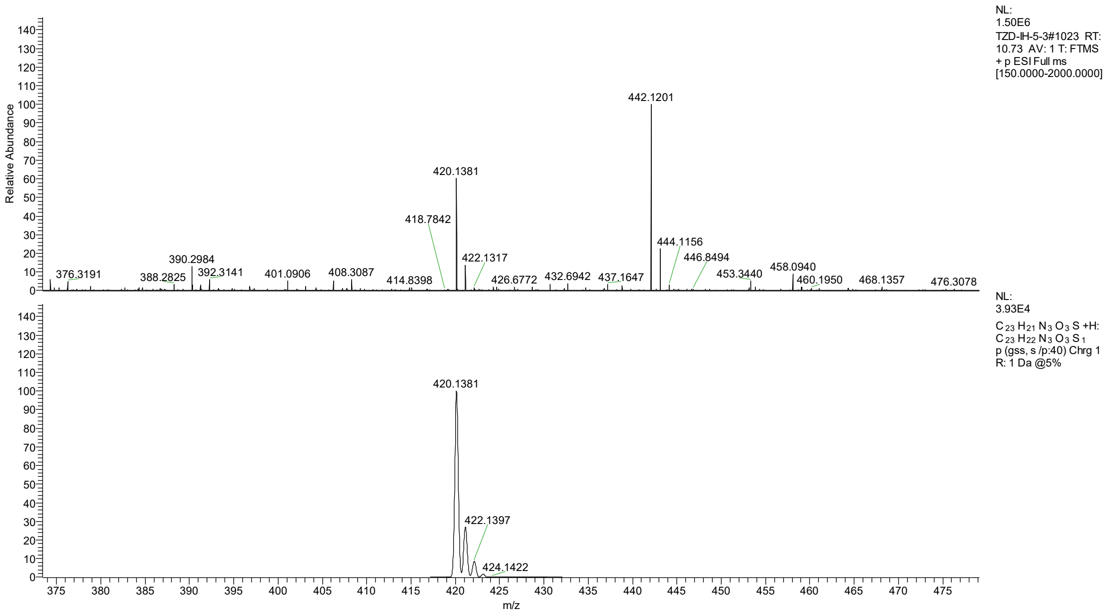

HRMS of compounds 5c

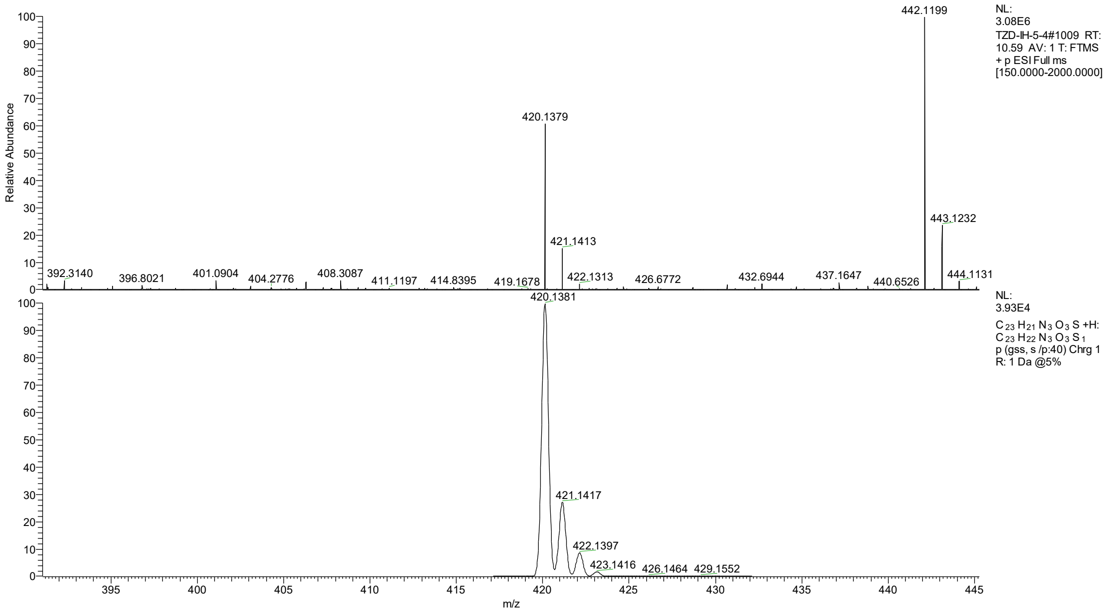

HRMS of compounds 5d

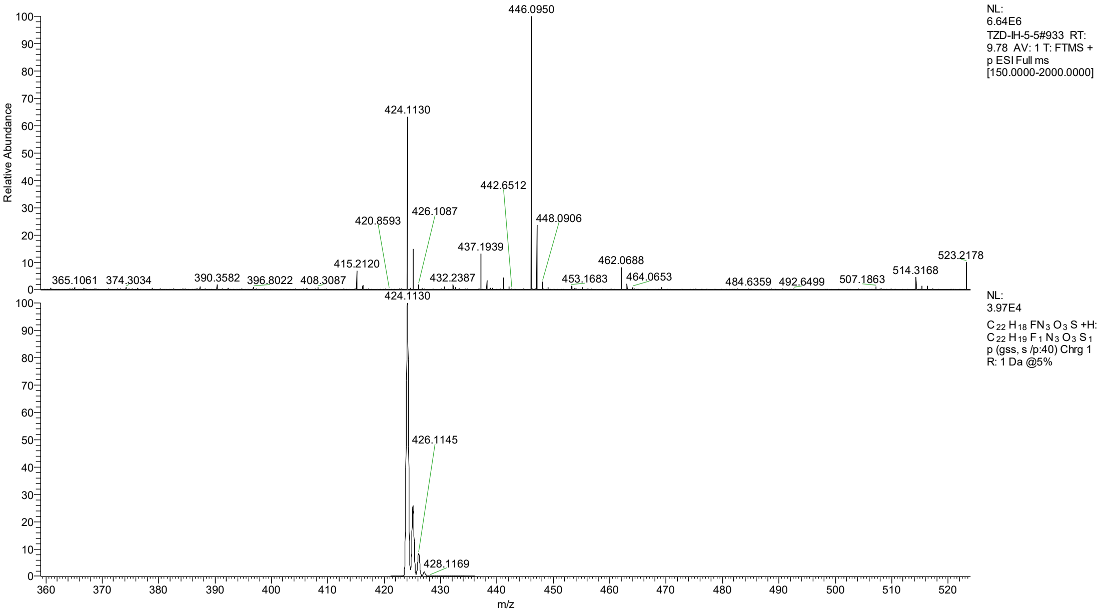

HRMS of compounds 5e

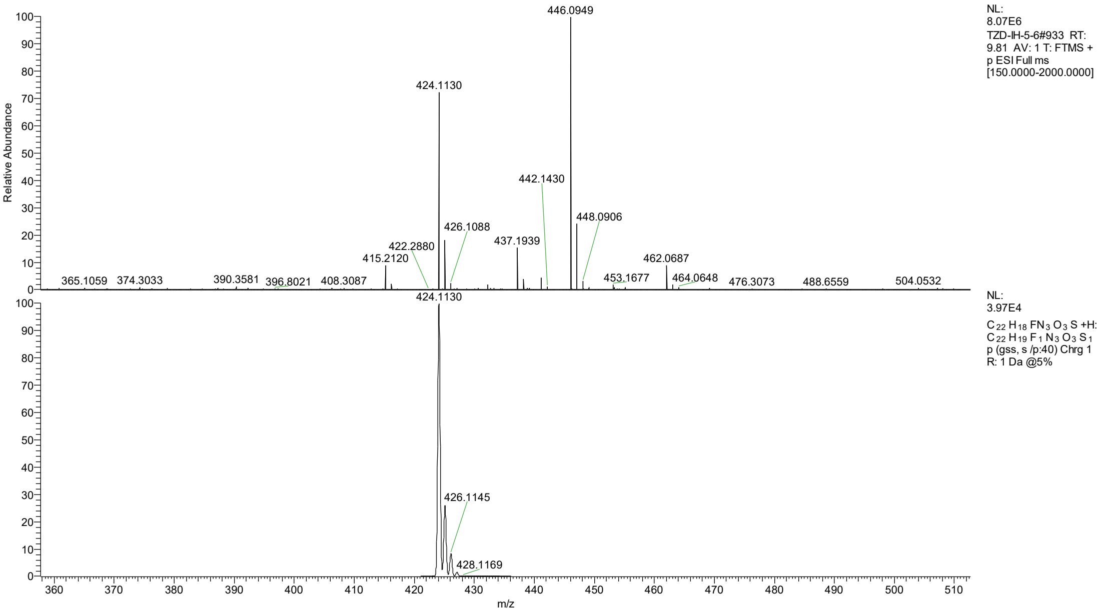

HRMS of compounds 5f

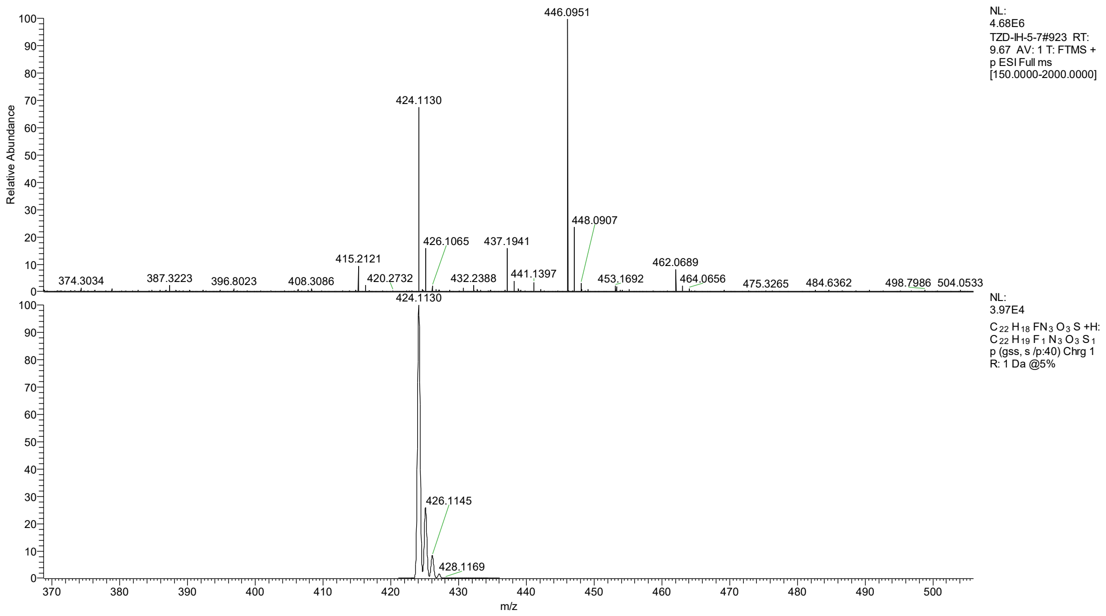

HRMS of compounds 5g

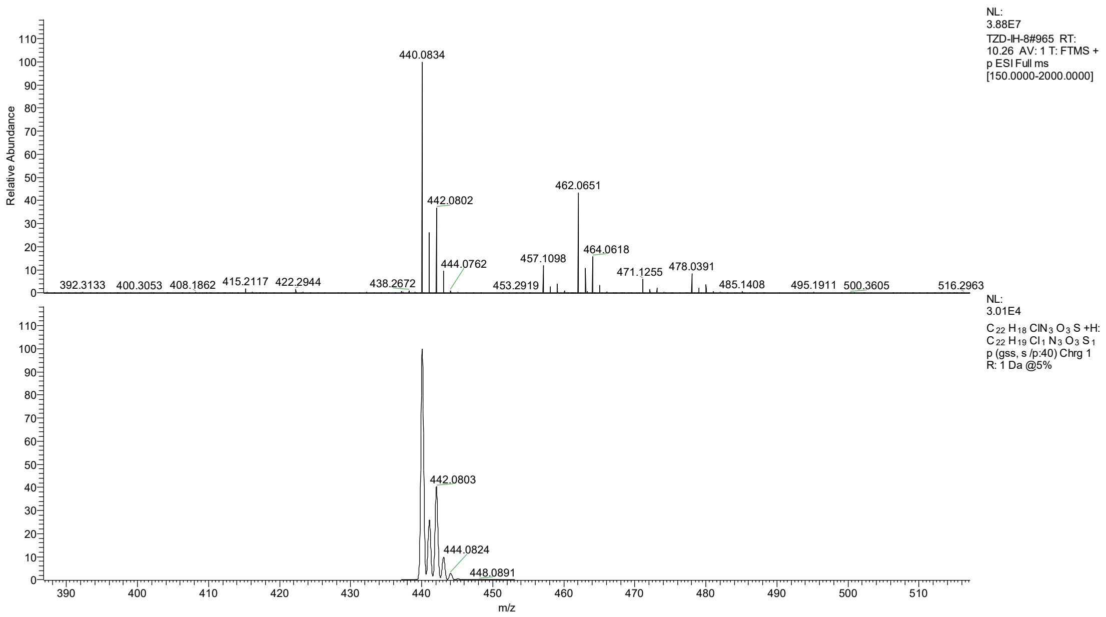

HRMS of compounds 5h

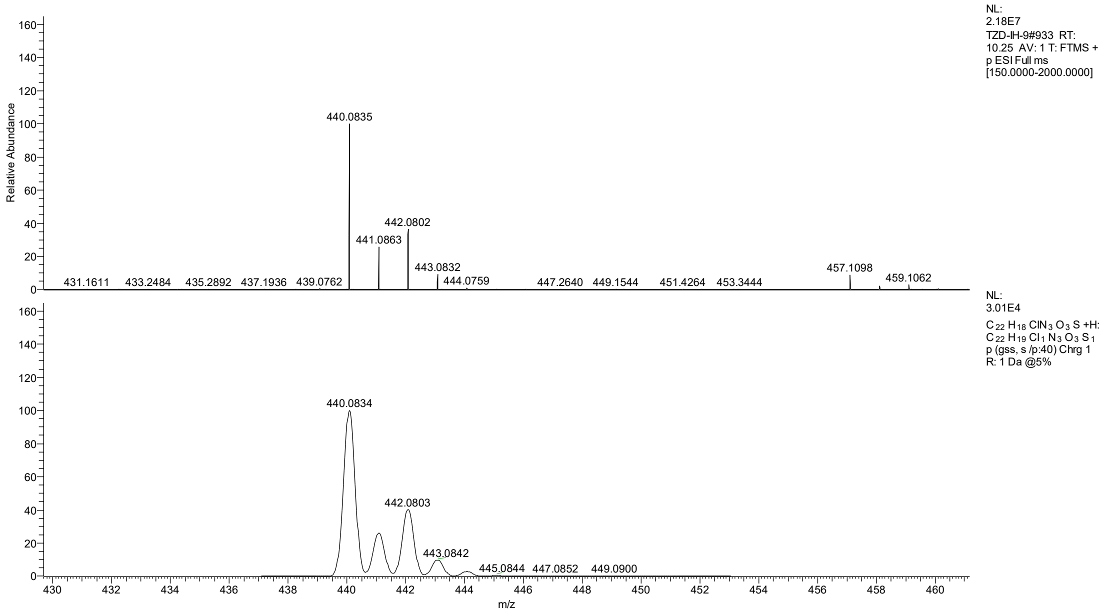

HRMS of compounds 5i

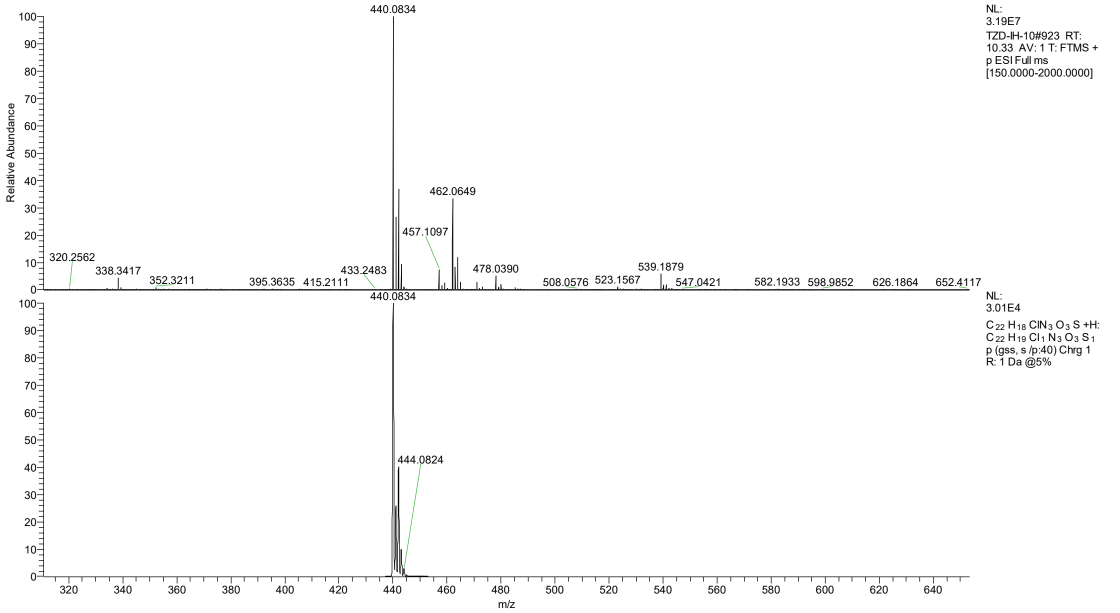

HRMS of compounds 5j

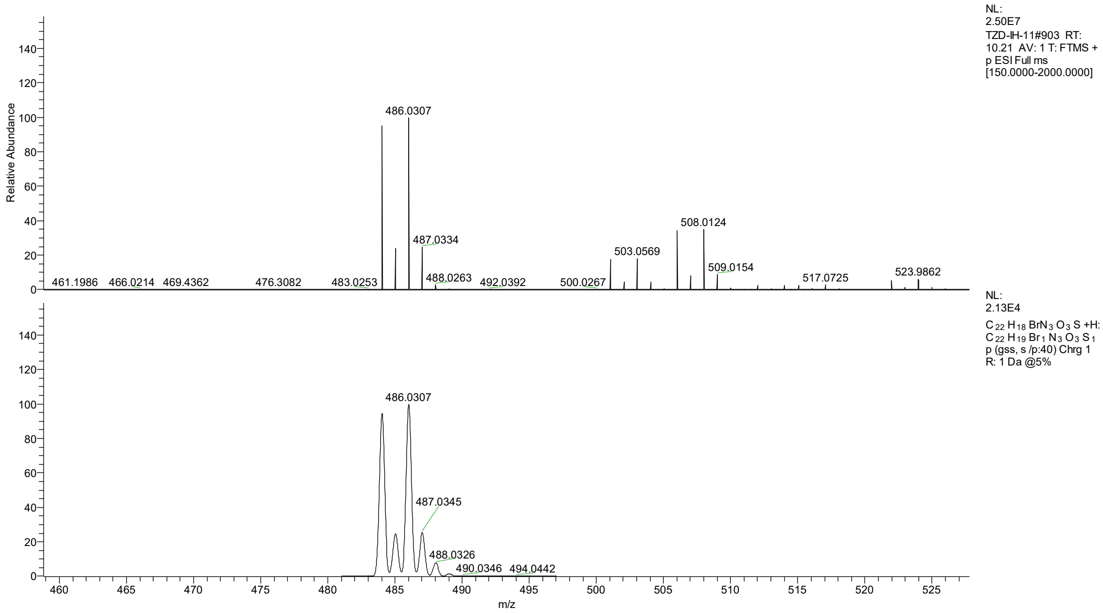

HRMS of compounds 5k

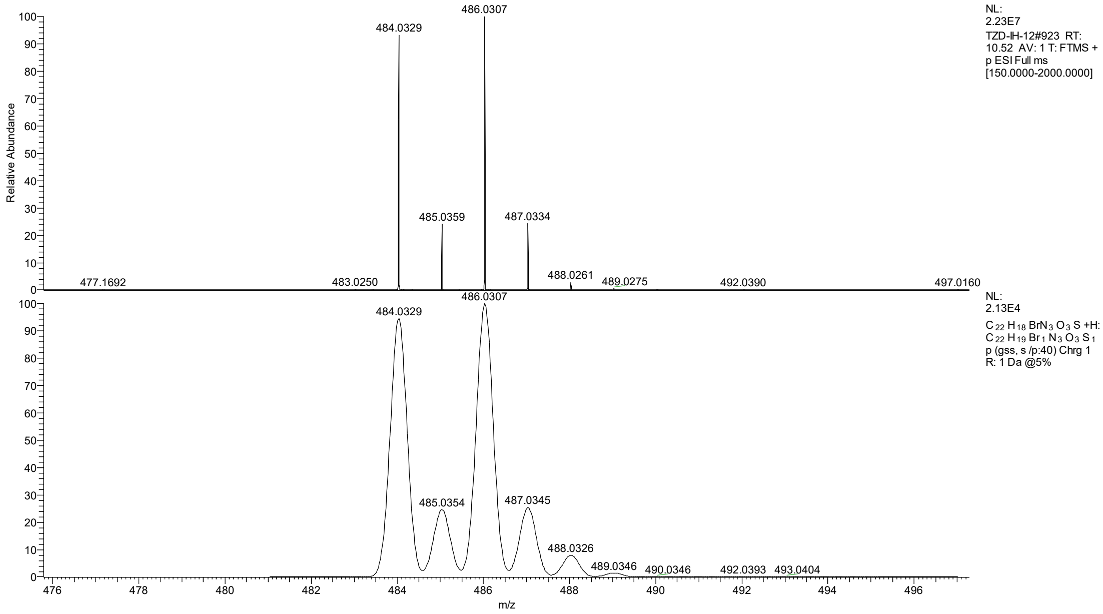

HRMS of compounds 5l

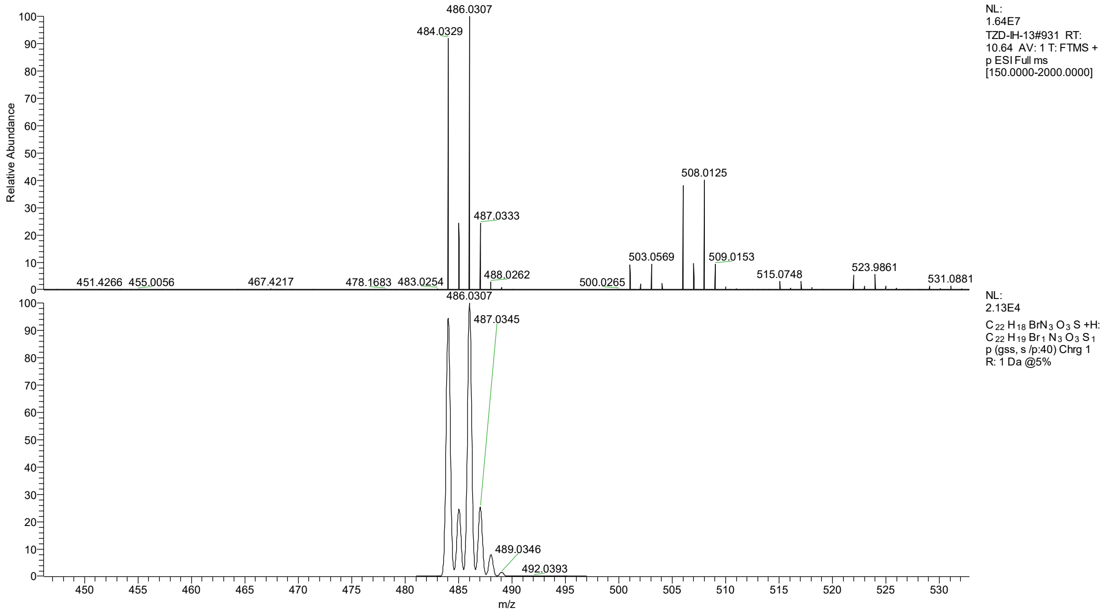

HRMS of compounds 5m

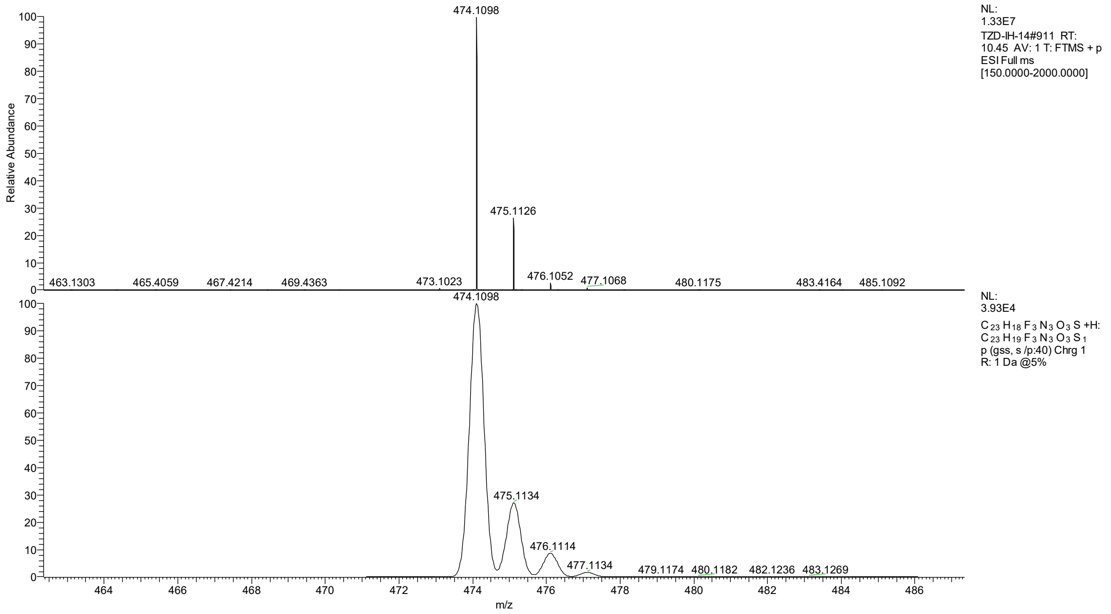

HRMS of compounds 5n

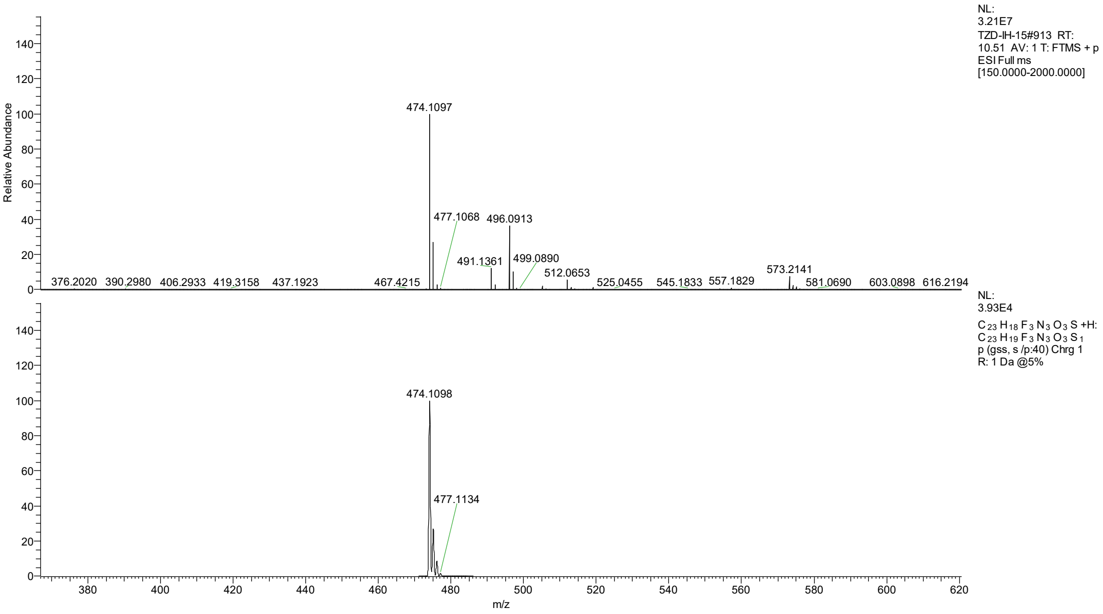

HRMS of compounds 5o

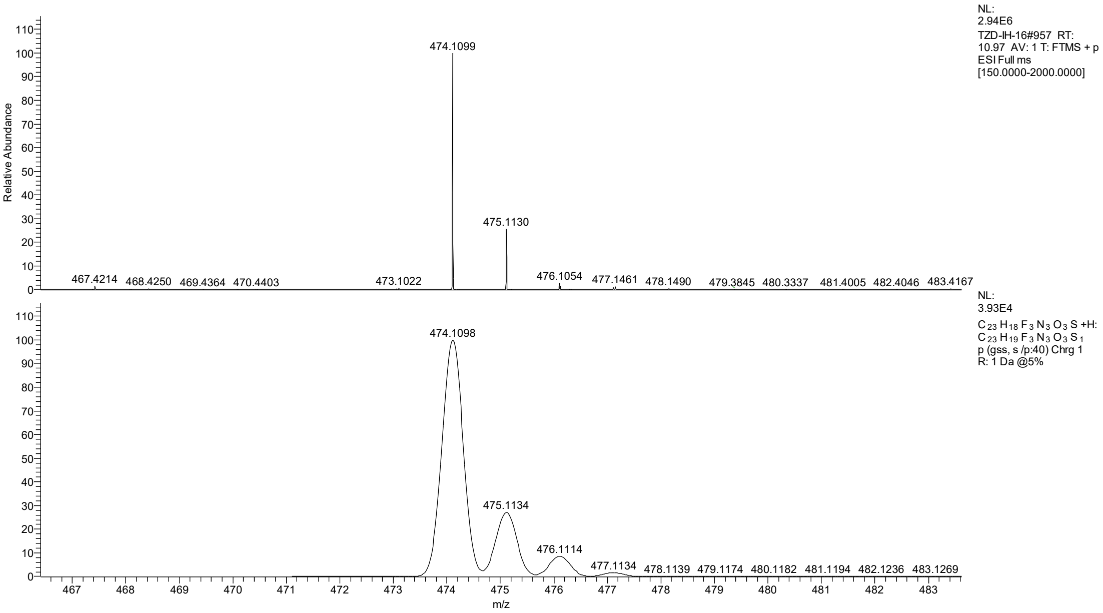

HRMS of compounds 5p

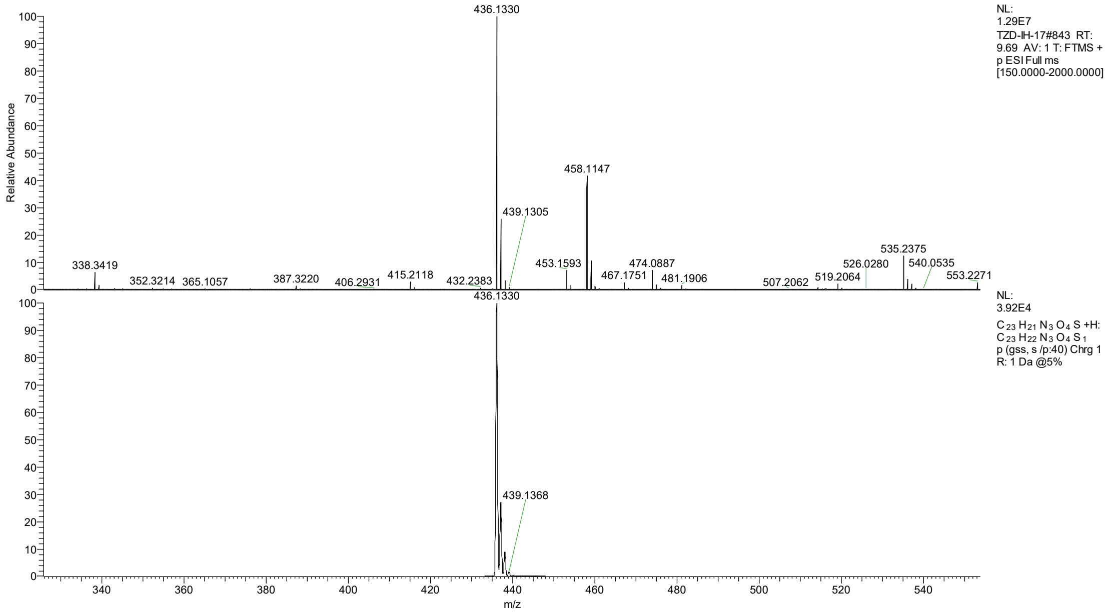

HRMS of compounds 5q

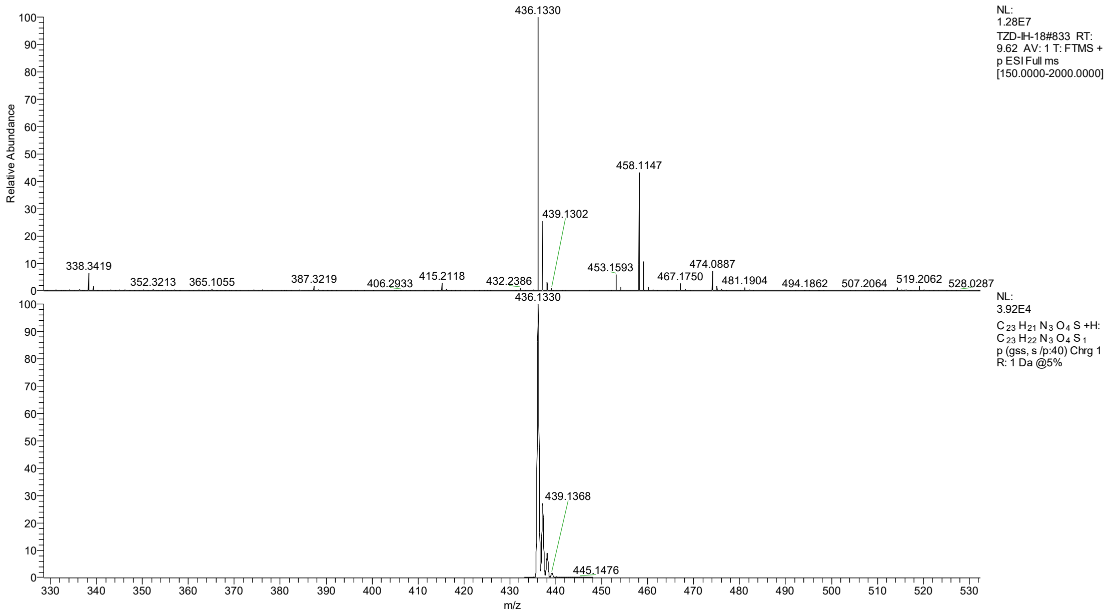

HRMS of compounds 5r

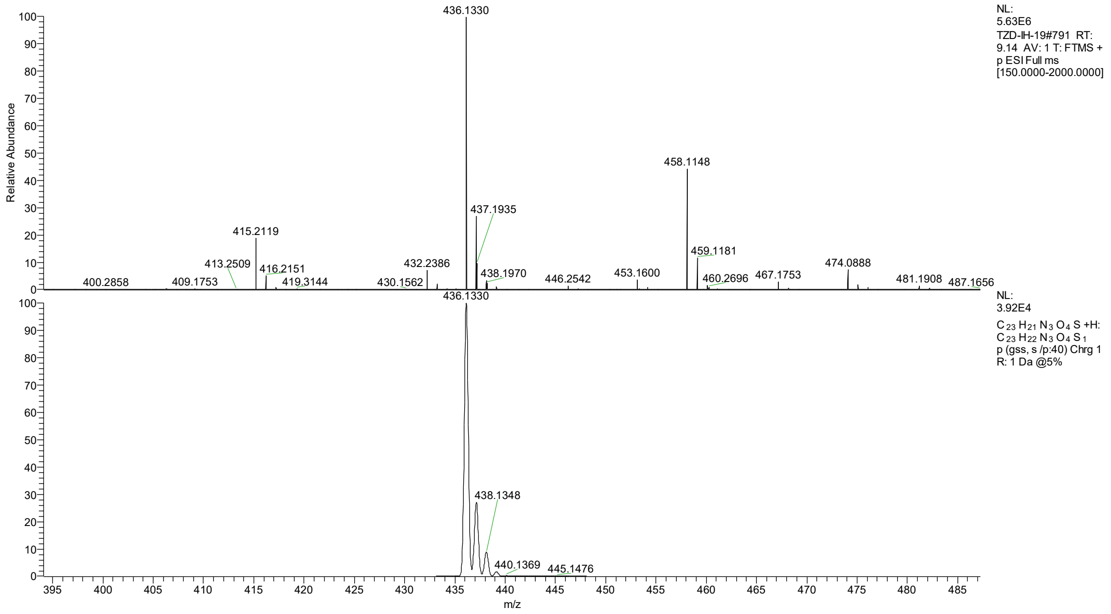

HRMS of compounds 5s

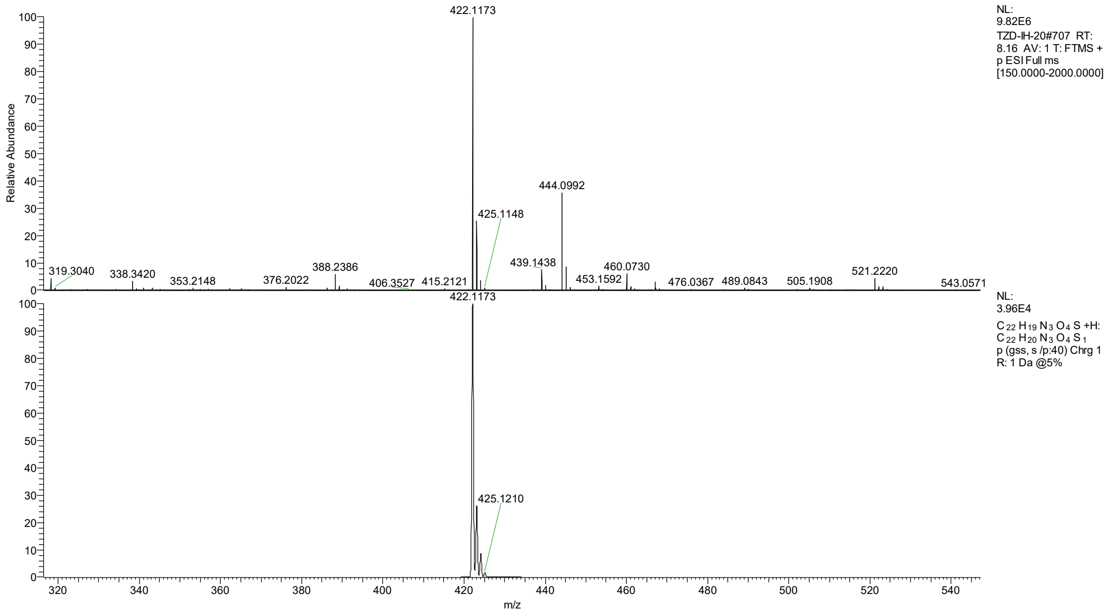

HRMS of compounds 5t

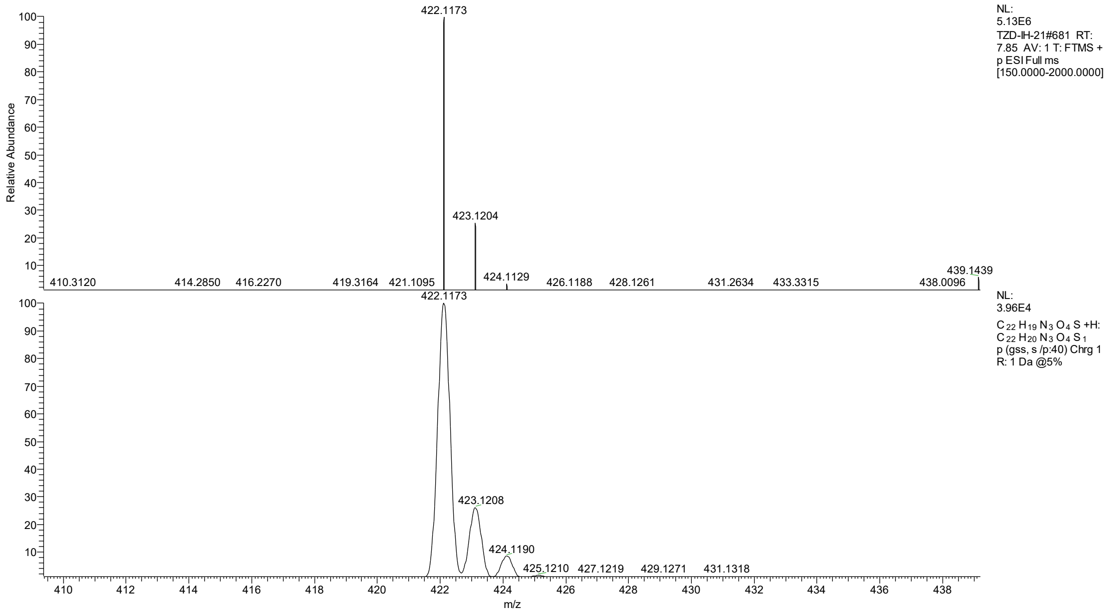

HRMS of compounds 5u

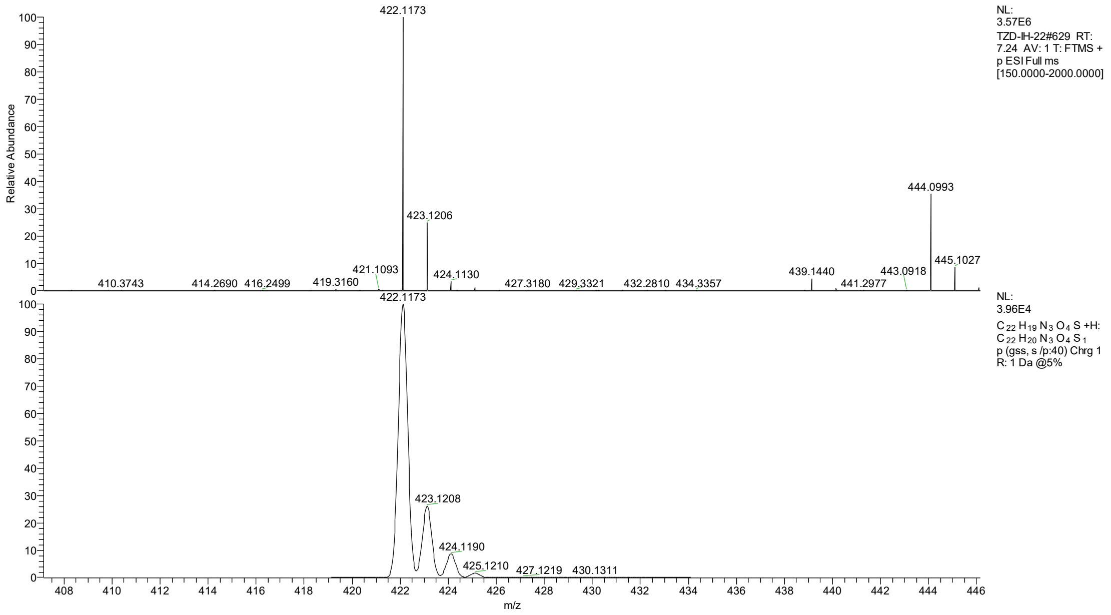

HRMS of compounds 5v

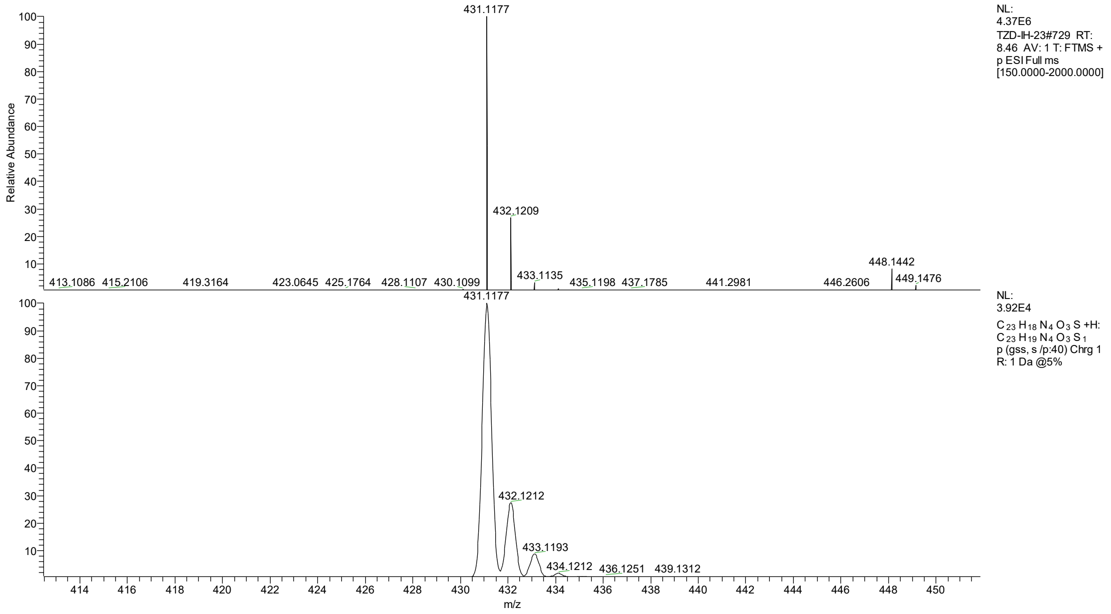

HRMS of compounds 5w

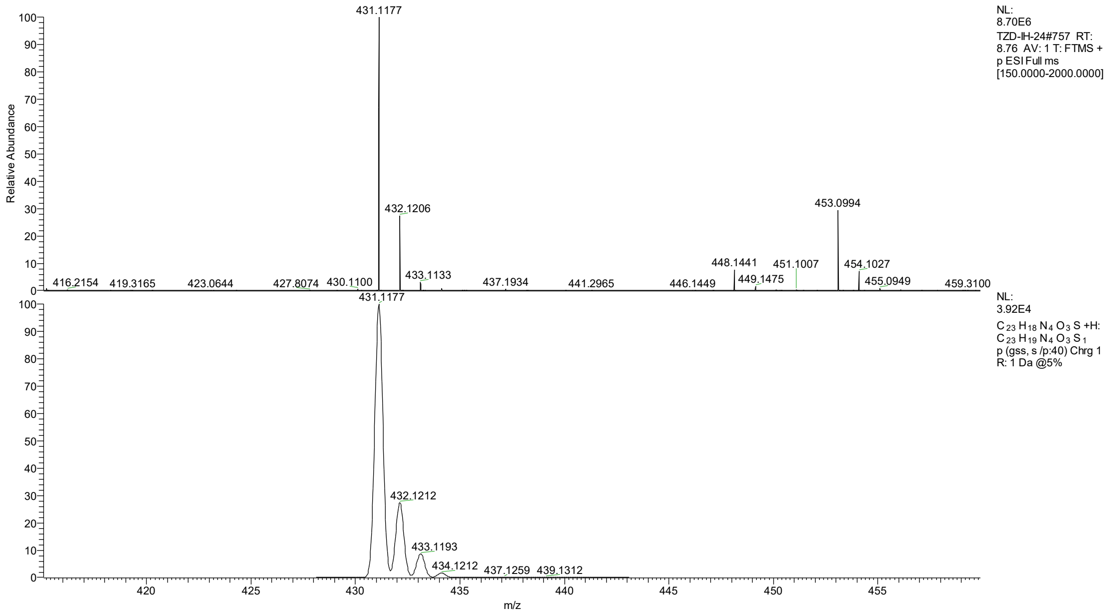

HRMS of compounds 5x

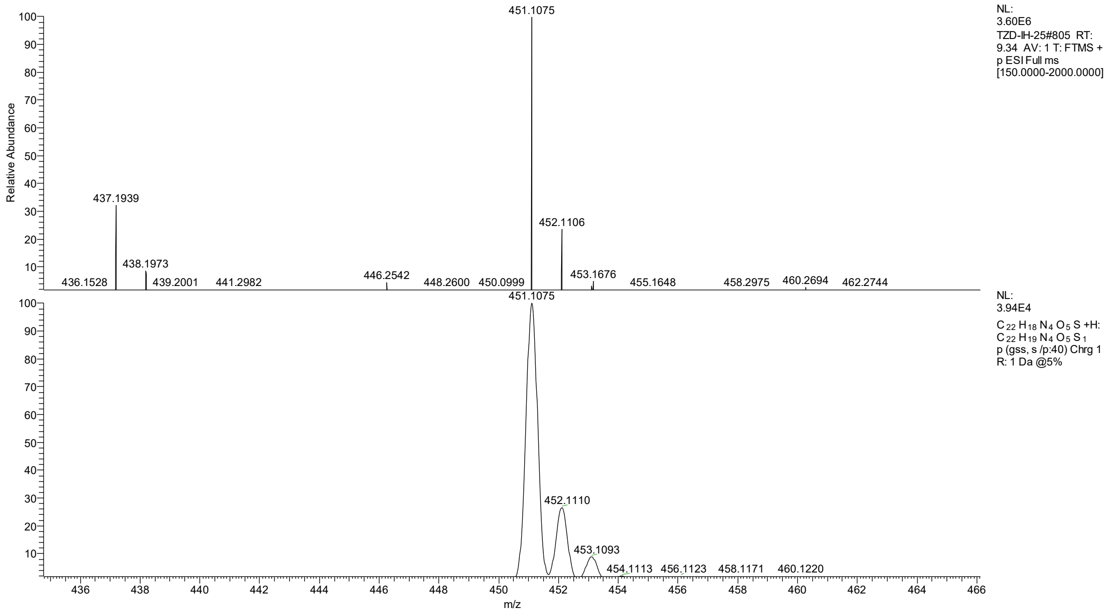

HRMS of compounds 5y

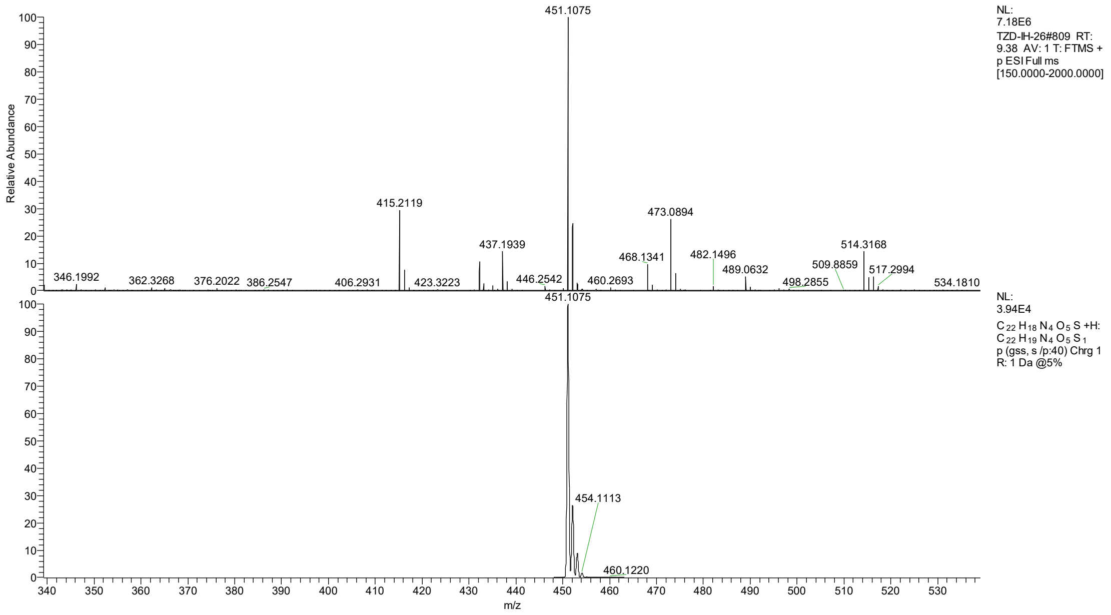

HRMS of compounds 5z

4. Cytotoxicity of 5w on B16F10 cells and zebrafish model

4. 1 Cytotoxicity of 5w and kojic acid on B16F10 cells

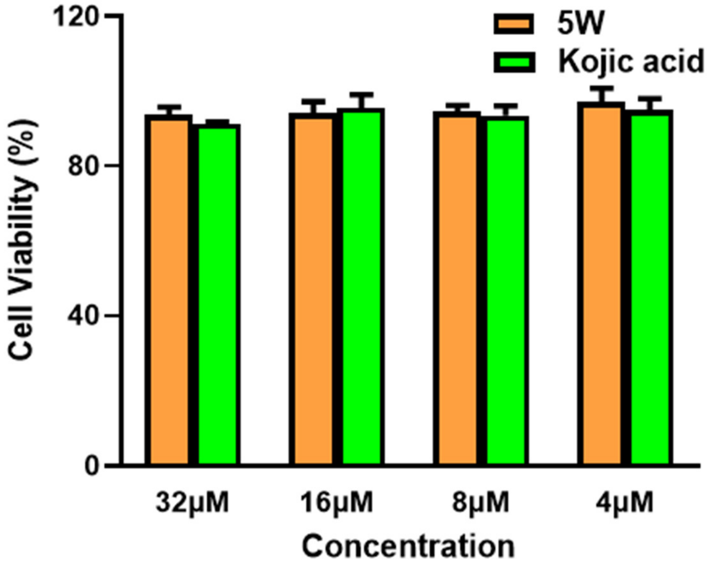

4. 2 Cytotoxicity of 5w and kojic acid on zebrafish

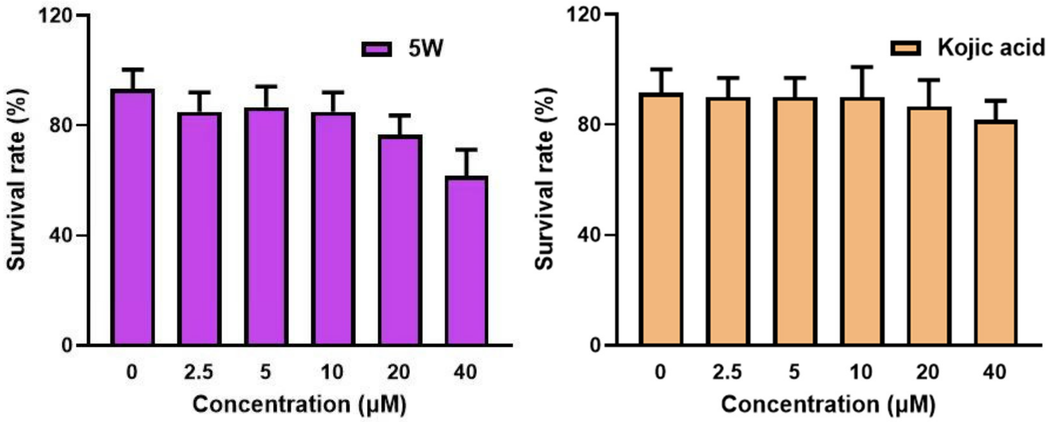

5. References

1. Pakhare, D.; Kusurkar, Radhika. Application of Horner-Wadsworth-Emmons olefination for the synthesis of granulatamide A, its E isomer and other amides of tryptamine. *New J. Chem.* **2016**, *40*, 5428-5431
2. Touaibia, M.; St-Coeur, P.; Duff, P.; Faye, D.C.; Pichaud, N. 5-Benzylidene, 5-benzyl, and 3-benzylthiazolidine-2,4-diones as potential inhibitors of the mitochondrial pyruvate carrier: Effects on mitochondrial functions and survival in Drosophila melanogaster. *Eur. J. Pharmacol.* **2021**, *913*, 174627.
3. Shaikh, F.M.; Patel, N.B.; Sanna, G.; Busonera, B.; Colla, P.L.; Rajani, D.P. Synthesis of some new 2-amino-6-thiocyanato benzothiazole derivatives bearing 2,4-thiazolidinediones and screening of their in vitro antimicrobial, antitubercular and antiviral activities. Med. Chem. Res. 2015, 24, 3129-3142.
